# Photo-induced copper-catalyzed alkynylation and amination of remote unactivated C(sp^3^)-H bonds†

**DOI:** 10.1039/d0sc05883a

**Published:** 2021-02-16

**Authors:** Zhusong Cao, Jianye Li, Youwen Sun, Hanwen Zhang, Xueling Mo, Xin Cao, Guozhu Zhang

**Affiliations:** Key Laboratory of Organometallic Chemistry, Shanghai Institute of Organic Chemistry, Center for Excellence in Molecular Synthesis, University of Chinese Academy of Sciences, Chinese Academy of Sciences 345 Lingling Road Shanghai 200032 P. R. China guozhuzhang@sioc.ac.cn; College of Chemistry, Central China Normal University (CCNU) 152 Luoyu Road Wuhan Hubei 430079 P. R. China; Zhongshan Hospital, Fudan University 180 Fenglin Road Shanghai 200032 P. R. China caox@fudan.edu.cn

## Abstract

A method for remote radical C–H alkynylation and amination of diverse aliphatic alcohols has been developed. The reaction features a copper nucleophile complex formed *in situ* as a photocatalyst, which reduces the silicon-tethered aliphatic iodide to an alkyl radical to initiate 1,*n*-hydrogen atom transfer. Unactivated secondary and tertiary C–H bonds at β, γ, and δ positions can be functionalized in a predictable manner.

## Abstract 2

Remote C−H alkynylation and amination of aliphatic alcohols.

## Graphical abstract

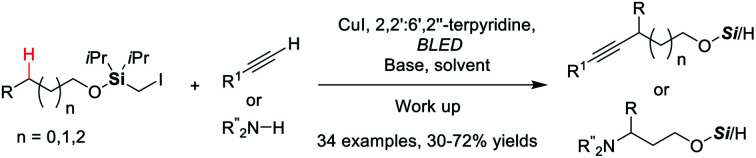

## Introduction

Direct functionalization of inert C(sp^3^)-H bonds provides an atom- and step-economic strategy for the rapid construction of valuable chemical frameworks.^1^ Along with transition-metal-mediated processes,^2^ radical-mediated hydrogen atom transfer (HAT) has emerged as an appealing and efficient strategy to activate and functionalize C(sp^3^)-H in a selective manner, and significant progress has been made in this field.^3^

**Scheme 1:**
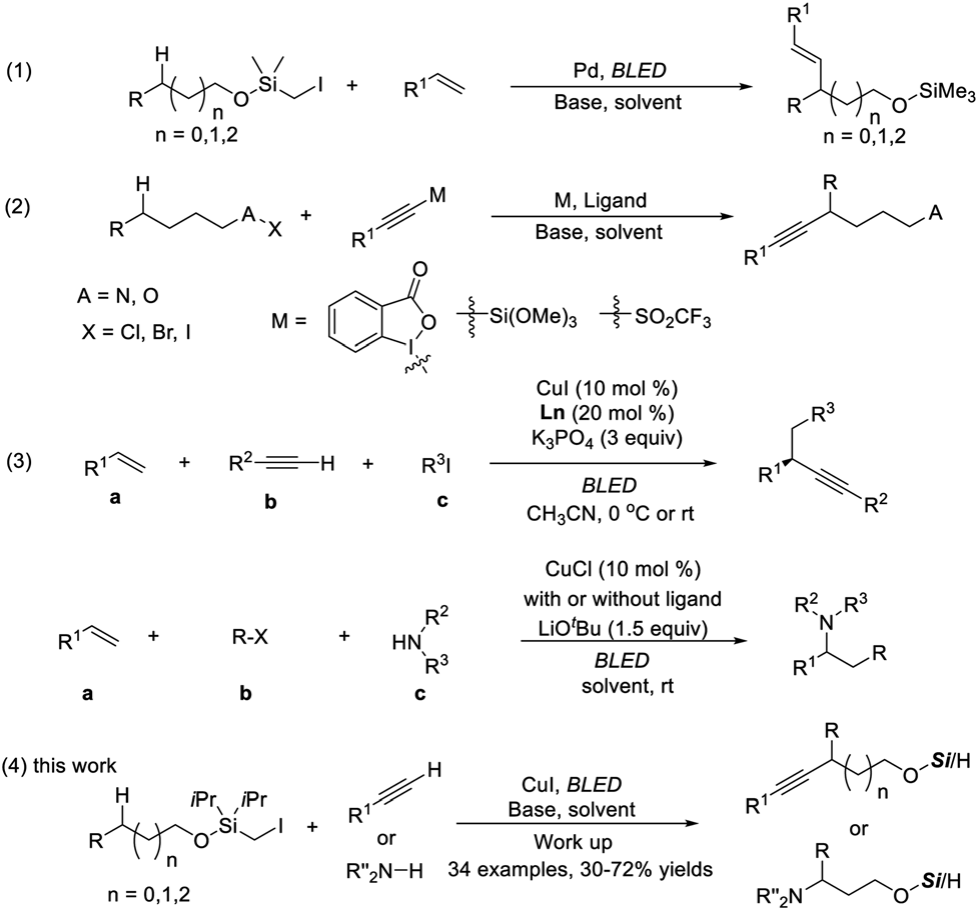
C–H alkynylation and amination of aliphatic alcohols.

An easily installed and removable hydroxyl protecting group, the (halomethyl)silyl group, has been established as an excellent carbon radical precursor and widely applied in radical-type reactions.^4^ Gevorgyan pioneered remote aliphatic C–H functionalization through radical transposition processes initiated with silicon-tethered carbon radicals.^5^ Particularly inspiring is the photoinduced palladium-catalyzed remote C–H Heck reaction, allowing ready access to alkenyl alcohols (Scheme 1, eqn (1)).^5*a*^

Benefitting from their electronic properties and various methods for further transformations, alkynes have been widely used as pivotal intermediates for the synthesis of complex biologically active or functional molecules.^6^ To our knowledge, remote C–H alkynylation at unactivated C–H bonds proceeding *via* intramolecular HAT has been explored only very recently, dominated by 1,5 transposition. Among the established methods, a polarized alkyne (X–Y) (*e.g.*, X≡R) is required and generally needs multistep synthesis (eqn (2)).^7^ Approaches allowing the use of a simple terminal alkyne in remote C–H bond alkynylation are limited and therefore highly desired.^8^

Photocatalysis using inexpensive and readily available copper complexes experienced a significant growth, exhibiting highly tunable redox properties and diverse reactivity.^9^ Fu pioneered photo-induced copper-catalyzed C–N bond formation using carbazole as the reactant and photocatalyst,^10^ while Hwang and Lalic demonstrated that copper could catalyze the coupling of acetylene with aryl^11^ and alkyl halides^12^ under light irradiation, with copper-acetylide acting as the photo-excitable intermediate.

## Results and discussion

Our group recently discovered a photo-catalytic system of copper(i) and realized the three-component carbon functionalization of alkenes (eqn (3)).^13*a*,*b*^ The significance of this work lies in the following: (1) the identification of in situ-formed photoactive Cu(i) species with significantly enhanced reducing capability,^13*c*,*d*^ and (2) the use of simple terminal alkynes and amines as coupling partners. Encouraged by the potential of C–H functionalization in synthesis, it was reckoned that carbon radical formation occurred from (halomethyl)silyl ether through Cu-acetylide photoreduction, followed by the selective 1,*n*-HAT. The newly formed radical could be recaptured by a Nu-copper species, which then furnished the remotely functionalized products.

Herein, the successful implementation of this hypothesis is described. In the presence of a single Cu complex, various mono-substituted alkynes and carbazoles undergo a site-selective radical relay alkynylation and amination reaction of aliphatic alcohols (eqn (4)). The reaction proceeds under mild visible-light-induced conditions at room temperature, producing β-, δ-, and γ-functionalized products selectively without the use of exogenous photosensitizers or external oxidants.

Our attempt began by allowing a model substrate Si-tethered iodide **1a** to react with 1-ethynyl-4-methylbenzene **2a** in the presence of the 2,2′:6′,2′′-terpyridine copper catalyst under blue-light irradiation. After comprehensive investigation of the reaction conditions, it was pleasing to find that the translocated Sonogashira product **3a** did form. Under the optimized conditions, **1a** provided the desired product **3a** in 60% yield after 32 h using CuI as the catalyst and 2,2′:6′,2′′-terpyridine as the ligand under blue-LED (BLED) irradiation (Table 1, entry 1). Under these conditions, the premature alkynylation at the Si-auxiliary site is suppressed to approximately 15%.

**Table 1:** Evaluation of ligands and other reaction parameters^a^

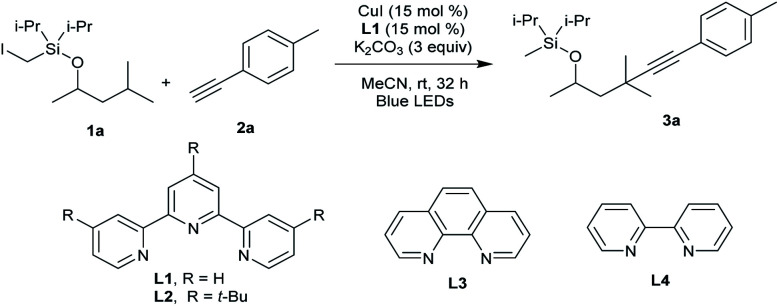		
Entry	Variation from the “standard conditions”	Yield^b^%
1	None	62(60^c^)
2	Without CuI, base or light	0
3	**L2** instead of **L1**	41
4	**L3** or **L4** instead of **L1**	0
5	K_3_PO_4_	39
6	Cs_2_CO_3_ or Na_2_CO_3_ instead of K_2_CO_3_	0
7	CuBr instead of CuI	35
8	CuTc instead of CuI	52
9	Cu(OTf)_2_ instead of CuI	32
10	DMF instead of MeCN	35
11	DCM instead of MeCN	38
12	Et_2_O or THF instead of MeCN	Trace
13	Reaction was performed at 10 °C	20
14^d^	Pd(OAc)_2_, xantphos and Cs_2_CO_3_ in PhH	0

a **1a** (0.1 mmol), **2a** (0.15 mmol), CuI (15 mol%), **L1** (15 mol%) and K_2_CO_3_ (3 equiv.) in MeCN, under N_2_, rt, blue LEDs, 32 h.

b Determined by ^1^H NMR analysis with internal standard (diethyl phthalate).

c Isolated yield.

d Pd(OAc)_2_ (10 mol%), xantphos (20 mol%) and Cs_2_CO_3_ (2 equiv.) in PhH, under N_2_, rt, blue LEDs, 32 h.

Control experiments determined that product **3a** was not produced in the absence of a copper salt, ligand, or base (entry 2). It was found that a structurally similar ligand with *t*Bu substitution on the terpyridine **L2** led to decreased yield (entry 3). Bidentate ligands **L3** and **L4** proved to be inefficient in this transformation (entry 4). Switching the base from K_2_CO_3_ to Cs_2_CO_3_, Na_2_CO_3_, or K_3_PO_4_ was detrimental to the reaction, and traces to small amounts of product were observed (entries 5 and 6). The performance of other copper salts such as CuBr and CuTc was also briefly examined, but they were less efficient for this reaction (entries 7–9). Further reaction optimization experiments identified MeCN as the best solvent (entries 10–12). The reaction running at lower temperature decreased the yield (entry 13). The use of the Pd catalyst gave no **3a**, highlighting the unique role of Cu in this tandem HAT and coupling process (entry 14).

After the optimal reaction conditions were established, the substrate scope with respect to alkynes was first investigated, keeping iodide **1a** as the substrate (Table 2). For aryl-substituted alkynes, various electronically different para-substituted phenylalkynes reacted to provide the corresponding C–H alkynylation products **3b–3g** in 39%–72% yields. Similar yields were obtained for alkynes bearing *ortho*- and *meta*-substituted aryl groups (**3h–3i**). 3-Ethynylpyridine was a competent substrate as well (**3j**). Notably, aliphatic and electron-deficient alkynes did not provide the desired product under current reaction conditions.

**Table 2:** Substrate scope studies with alkynes^a^

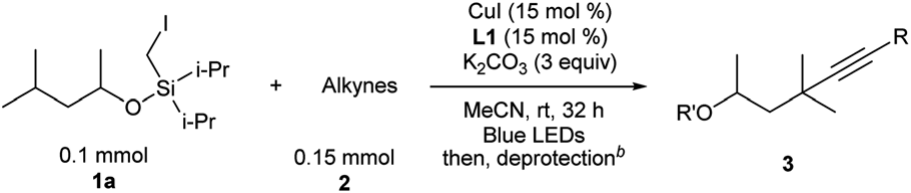
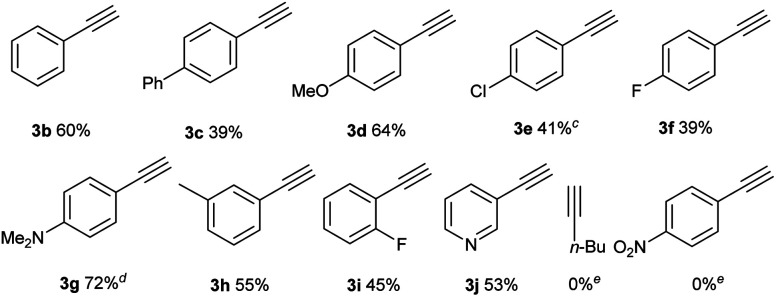

a **1a** (0.1 mmol), **2** (0.15 mmol), CuI (15 mol%), **L1** (15 mol%), and K_2_CO_3_ (3 equiv.) in MeCN (0.05 M), under N_2_, rt, blue LEDs, 32 h.

b Different deprotection procedures were applied depending on the products: TBAF in THF; or AcCl and montmorillonite K10 in CH_2_Cl_2_, See the ESI for details.

c 64 h.

d R′ = Ac.

e **1a** remained.

For Si-tethered alcohols (Table 3), although 1,5-HAT is kinetically less favorable than 1,6-HAT,^4*d*,14^ product **4a** was isolated in 30% yield. Next, the possibility of achieving a δ-Sonogashira reaction was examined; remarkably, selective δ-alkynylation of alcohols proceeded well (**4b**). Substrates containing competitive tertiary C–H sites (β- *vs.* γ-, and γ- *vs.* δ) were tested, and, as expected, γ-functionalized alkenols were obtained as the sole regioisomers (**4c** and **4d**).

**Table 3:** Substrate scope studies with alcohols^a^

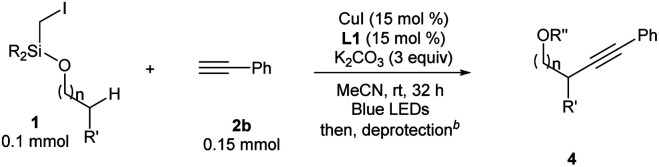
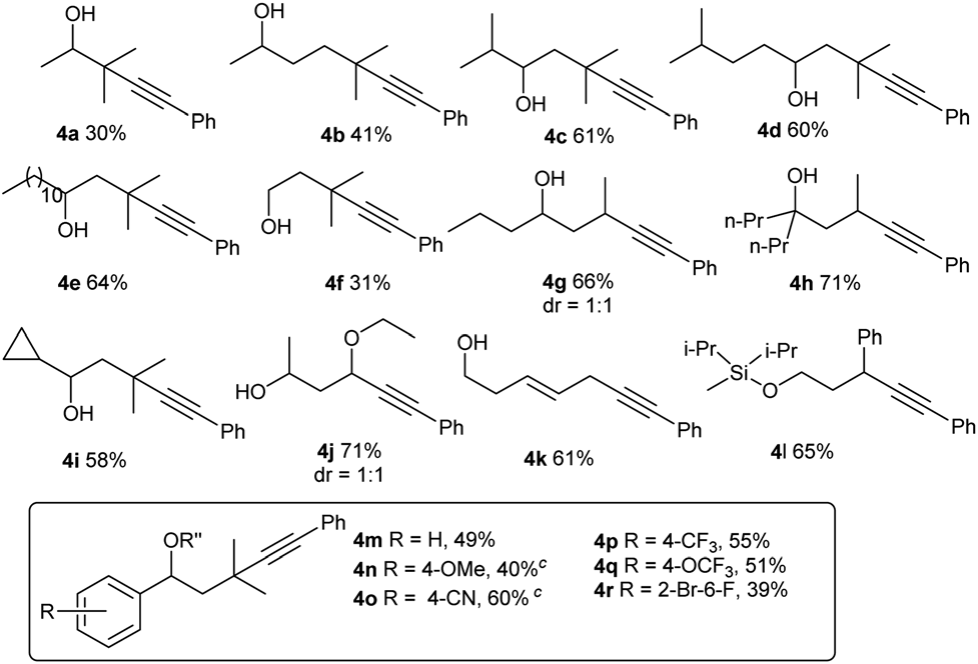

a **1a** (0.1 mmol), **2b** (0.15 mmol), CuI (15 mol%), **L1** (15 mol%), and K_2_CO_3_ (3 equiv.) in MeCN (0.05 M), under N_2_, rt, blue LEDs, 32 h. R = iPr for **4a–4g**, **4i–4r**; R = Me for **4h**.

b Different deprotection procedures were applied depending on the products: TBAF in THF; or AcCl and montmorillonite K10 in CH_2_Cl_2_. See the ESI for details.

c R′′ = Ac.

In Gevorgyan's remote Heck reaction, the γ-benzylic C–H alkenylation under Pd catalysis leads to the de-saturated by-product exclusively; in contrast, Cu-catalyzed γ-benzylic C–H alkynylation worked well (**4l**). Benzylic alcohols are capable substrates, and a range of functional groups were tolerated on the phenyl ring (**4m–4r**).

This photo-induced Cu-catalyzed remote C–H functionalization strategy could be expanded to C–H amination. The site-selective amination could readily take place at the tertiary and secondary C–H sites using 9*H*-carbazole as the reactant (**5a** and **5b**).^10,13*b*^ A benzylic position could be aminated as well (**5c**). The functional-group compatibility was investigated briefly, and Cl, MeO, and *t*Bu are readily allowed on the carbazole ring (**5d–5f**) (Table 4). The carbazole motif frequently occurs in natural products, drug molecules and chiral ligands, and the alkylated carbazoles obtained by this method are not easily accessed through other approaches.^15^

**Table 4:** Remote amination of aliphatic C–H bonds under photo-induced copper-catalyzed conditions^a^

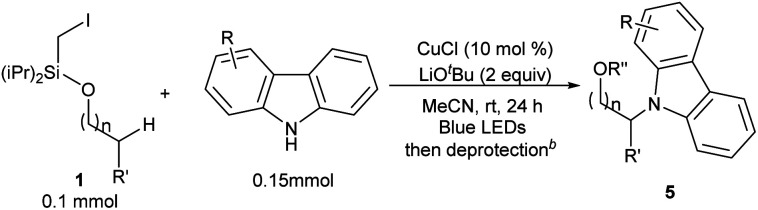
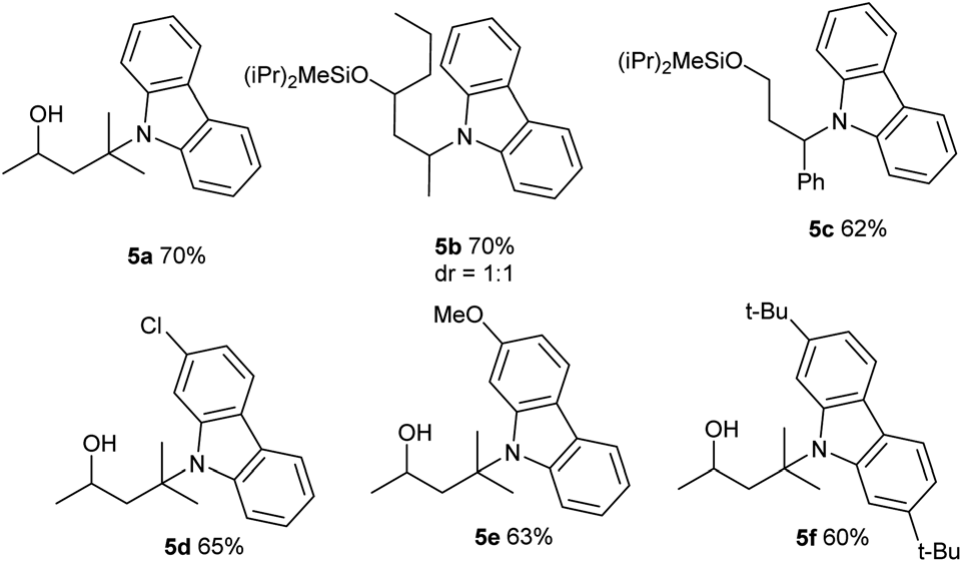

a **1a** (0.1 mmol), carbazole (0.15 mmol), CuCl (10 mol%), and LiO^*t*^Bu (2 equiv.) in MeCN (0.1 M), under N_2_, rt, blue LEDs, 24 h.

b deprotection by TBAF in THF. See the ESI for details.

**Scheme 2:**
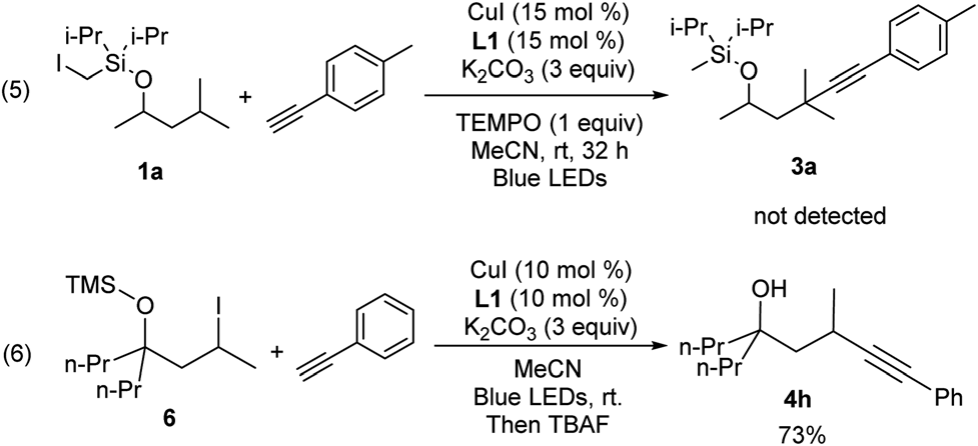
Mechanistic investigation.

In an effort to understand the mechanism of this transformation, some preliminary control experiments were conducted. With addition of TEMPO, the reactions were inhibited and no desired product **3a** was observed (Scheme 2, eqn (5)). Furthermore, to identify the possible intermediate, iodide **6** was independently synthesized and smoothly converted into **4h** under standard conditions (eqn (6)).

The UV-vis spectra of individual reagents or complexes were recorded at the reaction concentration in MeCN. **L1**-Cu-alkyne (should form *in situ* in the reaction) shows absorption in the range 380–500 nm.^13*a*^ In addition, the Stern–Volmer experiment indicated that the excited state of the **L1**-Cu-Nu complex formed *in situ* could be quenched by Si-iodide. These results suggest that a complex of nucleophile, copper, and base accounts for the photoactive species under BLED irradiation. Moreover, the quantum yield (*Φ* = 0.75%) suggested that a radical-chain process might not be involved (see the ESI† for details).

**Fig. 1:**
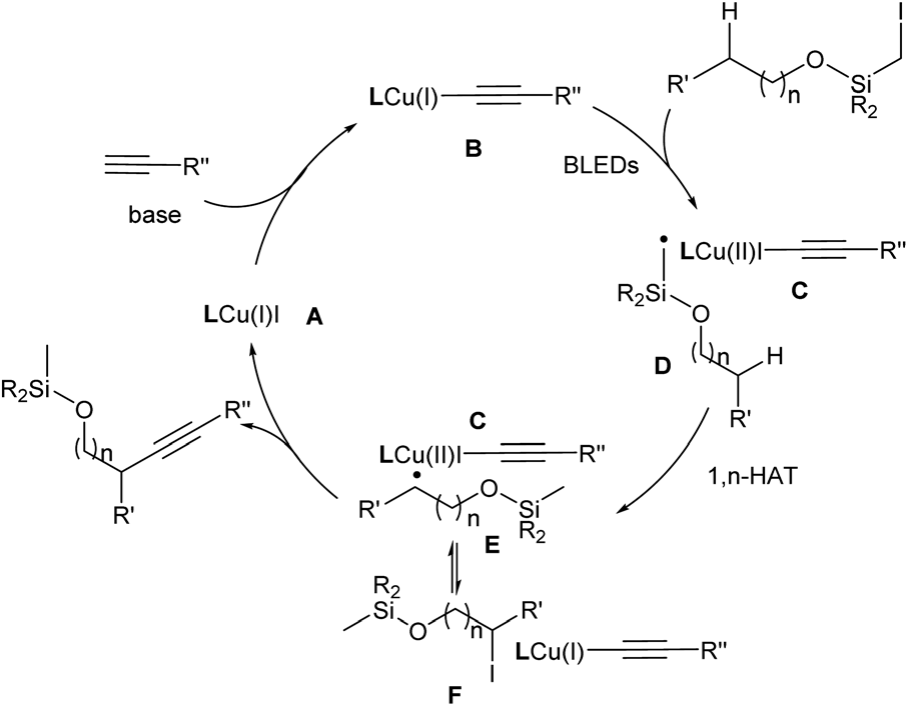
Proposed reaction mechanism.

Based on the literature^5*c*,12,13*a*^ and these findings, a reaction mechanism was proposed (Fig. 1). In the presence of a base, the [**L1**Cu(i)(C≡CR′′)] formed *in situ* serves as the photoactive species to undergo photoexcitation to generate [**L1**Cu(i)(C≡CR′′)]* (**B**). This intermediate delivers an electron to the alkyl halide, leading to [**L1**Cu(ii)(C≡CR′′)] (**C**) and an alkyl radical (R˙) (**D**). Subsequently, the latter undergoes a 1,*n*-HAT process, generating the translocated radical species (**E**). The radical species **E** could reversibly form the intermediate **F** either by a direct atom transfer from the CuI species or by recombination with the Cu complex followed by reductive elimination. Alternatively, instead of iodide, an alkyne could be delivered to afford the alkynylated product and regenerate the Cu catalyst. Related examples of this so-called radical relay strategy have been extensively studied by Liu,^3*g*^ Xiao,^16^ and Liu.^17^

**Scheme 3:**
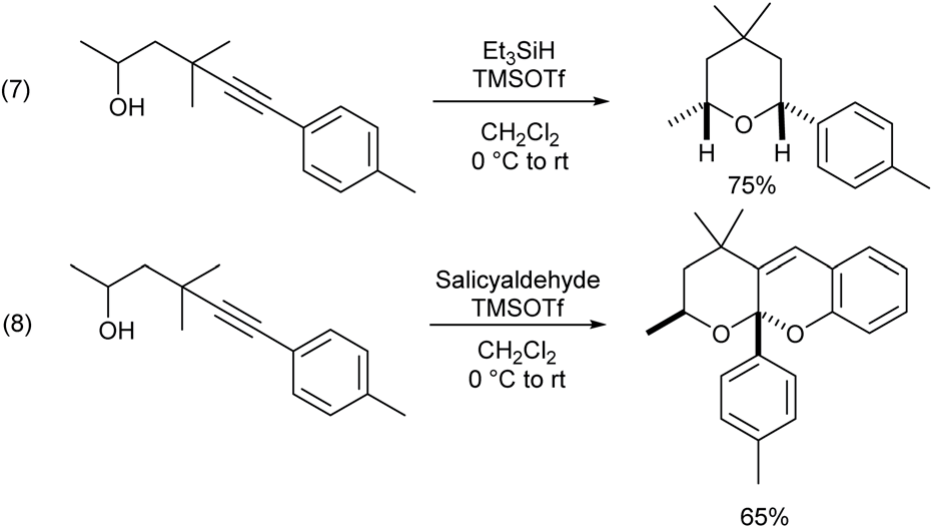
Transformations of γ-alkynyl alcohols.

γ-Alkynyl alcohols are valuable intermediates in organic synthesis, and they can participate in a wide range of transformations at triple bonds or hydroxyl groups. Thus, it can be shown that the γ-alkynylated product can be readily transformed into *cis*-tetrahydropyran through hydro hydroalkoxylation mediated by a Lewis acid (Scheme 3, eqn (7)). Only one more salicylaldehyde was added under the same conditions, and a cascade hydroalkoxylation–formal [4 + 2] cycloaddition reaction of the alkyne took place to construct tetrahydrofurano/pyrano chromene (eqn (8)).^18^

## Conclusions

In summary, in this work, a mild approach for visible-light-promoted, copper-catalyzed remote functionalization of C–H bonds of aliphatic alcohols at β, γ, and δ *via* a radical 1,*n*-HAT process is described. These reactions proceed at low temperature and are compatible with a range of functional groups. This system introduces the idea of merging visible-light photoredox catalysis with copper-catalyzed C–H functionalization. A single copper nucleophile complex serves as the photo-coupling catalyst and reactant as well. It is anticipated that this new methodology will find application in the C–H functionalization reactions directly using Nu–H.

## Conflicts of interest

There are no conflicts to declare.

## Supplementary Material

SC-012-D0SC05883A-s001
