# Supplementary material for: Photo-induced copper-catalyzed alkynylation and amination of remote unactivated C(sp3)-H bonds
Source: Chem Sci. 2021 Feb 16;12(13):4836–40. doi: 10.1039/d0sc05883a (PMC8179574; doi:10.1039/d0sc05883a)
Supplement: SC-012-D0SC05883A-s001 [file SC-012-D0SC05883A-s001.pdf]

Electronic Supplementary Material (ESI) for Chemical Science.
This journal is © The Royal Society of Chemistry 2021

Photo-Induced Copper-Catalyzed Alkynylation and Amination of Remote Unactivated C(sp3)−H bond

Zhusong Cao,[a] Jianye Li,[b] Youwen Sun,[a] Hanwen Zhang,[a] Xueling Mo,[a] Xin Cao[c,]* and Guozhu Zhang[a, b,]*

[a] State Key Laboratory of Organometallic Chemistry, Shanghai Institute of Organic Chemistry, Center for Excellence in Molecular Synthesis, University of Chinese Academy of Sciences, Chinese Academy of Sciences, 345 Lingling Road, Shanghai 200032, P. R. China

[b] College of Chemistry, Central China Normal University (CCNU), 152 Luoyu Road, Wuhan, Hubei 430079, P. R. China.

[c] Zhongshan Hospital, Fudan University, Shanghai, China. 180 Fenglin Road, Shanghai 200032, P. R. China.

Content

1. General information 2
2. Synthesis and characterizations of substrates 2
   1.1 General procedure for the synthesis of tethered alcohols 2
   2.2 Characterizations of new substrates 2
3. Experimental Details 7
   3.1 Optimization of the reaction conditions 7
   3.2 Genernal procedures and characterizations of products 9
   3.3 Characterization data of products 10
4. Synthetic applications 23
   4.1 Stereoselective Synthesis of tetrahydropyran derivative 23
   4.2 Stereoselective synthesis of pyrano-chromene derivative 23
5. Mechanistic investigations 24
   5.1 Radical scavengers studies 24
   5.2 Observation of intermediate 24
   5.3 Photophysical study 25
   5.4 Quantum yield measurement 27
6. References 28
7. Spectral Data 29

1. General information

NMR spectra were recorded on Varian Inova (400 MHz), Bruker (400 MHz) or Aglient (400 MHz) spectrometer. The ^{1}H NMR were calibrated against the peak of tetramethylsilane at 0 ppm). The ^{13}C NMR were calibrated against the peak of the $CDCl_3$ at 77.16 ppm. The multiplicities ^{1}H NMR spectra are abbreviated as follows: s (singlet) d (doublet) t (triplet) q (quartet) and m (multiplet). GC/MS analysis was performed on GCMS-QP2010 Plus. HRMS analyses were performed on Bruker maXis, Thermo Scientific Q Exactive HF Orbitrap-FTMS and Waters premier GC-TOF MS. All IR spectra was processed on Nicolet 380 and the method is denoted in brackets. UV/Vis absorption spectra were recorded on Varian Cary 100. Fluorescence spectrum was o performed on HITACHI F-2700. Materials: Unless stated otherwise, acetonitrile and the substrates were purchased from commercial sources without further purification. Blue LED lamps (40W, Kessil A160WE tuna blue) were used for reactions. Reactions requiring inert conditions were carried out in glove box.

2. Synthesis and characterization of substrates

All the tethered alcohols were prepared by conventional methods.[1] Alcohol precursors not commercially available were synthesized from the corresponding aldehydes. Chloro(iodomethyl)diisopropylsilane was prepared according to literature procedure.[2]

1.1 General procedure for the synthesis of tethered alcohols

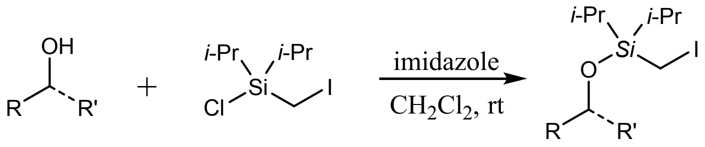

Under nitrogen atmosphere, to a 25 mL round bottom flask was added imidazole (272 mg, 4 mmol, 2 equiv) in 5 mL CH_2Cl_2, Chloro(iodomethyl)diisopropylsilane (640 mg, 2.2 mmol, 1.1 equiv) and corresponding alcohol. The mixture was stirred until completion of the reaction as judged by GC/MS analysis. The mixture was then concentrated under reduced pressure. The residue was purified by column chromatography in hexanes.

2.2 Characterizations of new substrates

((2,7-dimethyloctan-4-yl)oxy)(iodomethyl)diisopropylsilane

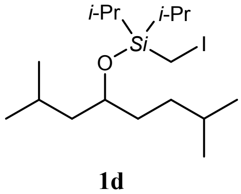

1d

1d was prepared according to general procedure in 75% isolated yield. Colorless oil. ^{1}H NMR (400 MHz, $CDCl_3$): δ 3.96-3.88 (m, 1H), 2.08 (s, 2H), 1.75-1.63(m, 1H), 1.58-1.42 (m, 3H), 1.39-1.30 (m, 2H), 1.27-1.15 (m, 4H), 1.13-1.05 (m, 12H), 0.94-0.83 (m, 12H). ^{13}C NMR ($CDCl_3$, 101 MHz): δ 71.63, 46.25, 35.11, 33.79, 28.34, 24.61, 23.104, 23.096, 22.79, 22.64, 17.92, 17.89, 17.61, 17.58, 12.79, 12.75. IR (neat) cm^{-1} ṽ: 2953, 2867, 1464, 1367, 1139, 1052, 1003, 882, 722, 679. HRMS (EI, 70eV) m/z calcd. for $C_{17}H_{37}IOSi$ [M]$^+$: 412.1653; found: 412.1657.

(1-cyclopropyl-3-methylbutoxy)(iodomethyl)diisopropylsilane

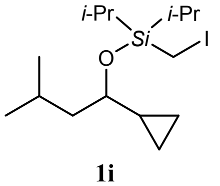

1i

1i was prepared according to general procedure in 89% isolated yield. Colorless oil. ^{1}H NMR (400 MHz, $CDCl_3$): δ 3.36-3.29 (m, 1H), 2.10 (s, 2H), 1.90-1.79 (m, 1H), 1.58-1.48 (m, 1H), 1.44-1.36 (m, 1H), 1.28-1.16 (m, 2H), 1.12-1.05 (m, 12H), 0.90 (dd, J = 6.6, 5.0 Hz, 5H), 0.50- 0.45(m, 2H), 0.36-0.30 (m, 1H), 0.22-0.16 (m, 1H). ^{13}C NMR (101 MHz, $CDCl_3$): δ 75.76, 48.04, 24.50, 23.46, 23.02, 18.16, 18.11, 17.99, 17.91, 17.80, 13.24, 13.08, 4.05, 2.79. IR (neat) cm^{-1} ṽ: 2952, 2866, 1463, 1382, 1367, 1087, 1060, 1014, 919, 882, 721, 681, 520. HRMS (EI, 70eV) m/z calcd. for $C_{15}H_{31}IOSi$ [M]$^+$: 382.1183; found: 382.1192.

((4-ethoxybutan-2-yl)oxy)(iodomethyl)diisopropylsilane

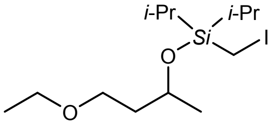

1j

1j was prepared according to general procedure in 78% isolated yield. Colorless oil. ^{1}H NMR (400 MHz, $CDCl_3$): δ 4.19-4.10 (m, 1H), 3.54-3.41m, 4H), 2.07 (d, J = 0.9 Hz, 2H), 1.75-1.67 (m, 2H), 1.25-1.15 (m, 8H), 1.10-1.04 (m, 12H). ^{13}C NMR (101 MHz, $CDCl_3$) δ 67.26, 66.65, 66.27, 39.78, 24.34, 17.95, 17.86, 17.61, 17.59, 15.37, 12.71, 12.67. ^{13}C NMR (101 MHz, $CDCl_3$): δ 157.88, 136.74, 126.355, 112.59, 72.94, 54.30, 49.42, 23.58, 22.03, 17.08, 16.72, 16.63, 16.47, 11.69, 11.52, -20.66. IR (neat) cm^{-1} ṽ: 2941, 2865, 1462, 1377, 1117, 1051, 1027, 953, 882, 719, 680. HRMS (EI, 70eV) m/z calcd. for $C_{13}H_{29}IO_2Si$ [M]$^+$: 372.0976; found: 372.0978.

(iodomethyl)diisopropyl(pent-4-en-1-yloxy)silane

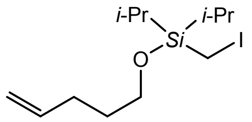

1k

1k was prepared according to general procedure in 85% isolated yield. Colorless oil. 1H NMR (400 MHz, $CDCl_3$): δ 5.88-5.77 (m, 1H), 5.05-4.93 (m, 2H), 3.75 (t, J = 6.4 Hz, 2H), 2.17-2.10 (m, 2H), 2.07 (s, 2H), 1.70-1.59 (m, 2H), 1.27-1.16 (m, 2H), 1.10-1.04 (m, 12H). ^{13}C NMR (101 MHz, $CDCl_3$): δ 138.38, 114.60, 63.14, 31.99, 29.96, 17.68, 17.44, 12.27. IR (neat) cm^{-1} ṽ: 2940, 2866, 1641, 1462, 1099, 992, 911, 881, 804, 723, 684. HRMS (EI, 70eV) m/z calcd. for $C_{12}H_{25}IOSi$ $[M]^+$: 340.0714; found: 340.0716.

(iodomethyl)diisopropyl(3-phenylpropoxy)silane

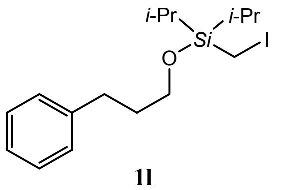

1l

1l was prepared according to general procedure in 80% isolated yield. Colorless oil. ^1H NMR (400 MHz, $CDCl_3$): δ 7.33-7.27 (m, 2H), 7.25-7.17 (m, 3H), 3.80(t, J = 6.3 Hz, 2H), 2.73 (t, J = 7.7 Hz 2H), 2.10 (s, 2H), 1.95-1.85 (m, 2H), 1.32-1.20 (m, 2H), 1.13-1.07 (m, 12H). ^{13}C NMR (101 MHz, $CDCl_3$): δ 142.24, 128.60, 128.42, 125.84, 63.15, 34.59, 32.17, 17.83, 17.61, 12.43. IR (neat) cm^{-1} ṽ: 2941, 2864, 1458, 1381, 1102, 964, 881, 786, 723, 697. HRMS (EI, 70eV) m/z calcd. for $C_{16}H_{27}IOSi$ $[M]^+$: 390.0870; found: 390.0878.

(iodomethyl)diisopropyl(3-methyl-1-phenylbutoxy)silane

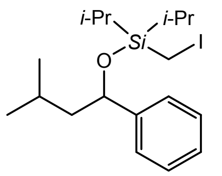

1m

1m was prepared according to general procedure in 79% isolated yield. Colorless oil. ^1H NMR (400 MHz, $CDCl_3$): δ 7.33-7.29 (m, 4H), 7.27-7.21 (m, 1H), 4.86 (t, J = 6.3 Hz, 1H), 1.89 (d, J = 1.6 Hz, 2H), 1.78-1.69 (m, 1H), 1.63-1.54 (m, 1H), 1.52-1.42 (m, 1H), 1.26-1.02 (m, 8H), 1.01-0.86 (m, 1H). ^{13}C NMR (101 MHz, $CDCl_3$): δ 144.56, 127.28, 126.40, 125.24, 73.41, 49.44, 23.56, 22.13, 22.01, 17.07, 16.71, 16.62, 16.46, 11.69, 11.52, -20.81. IR (neat) cm^{-1} ṽ: 2952, 2866, 1462, 1366, 1060, 1006, 915, 882,

856, 804, 761, 727, 698, 552. HRMS (ESI) m/z calcd. for $C_{18}H_{31}INaOSi$ $[M+Na]^+$: 413.0768; found: 413.0788.

(iodomethyl)diisopropyl(1-(4-methoxyphenyl)-3-methylbutoxy)silane

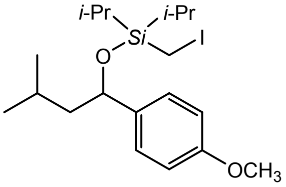

1n

1n was prepared according to general procedure in 85% isolated yield. Colorless oil. ^{1}H NMR (400 MHz, $CDCl_3$): δ 7.23 (d, J = 8.6 Hz, 2H), 6.87 (d, J = 8.5 Hz, 2H), 4.82 (t, J = 6.7 Hz, 1H), 3.81 (s, 3H), 1.88 (d, J =3.8Hz, 2H), 1.77-1.67 (m, 1H), 1.60-1.51 (m, 1H), 1.50-1.41 (m, 1H), 1.25-1.04 (m, 8H), 1.02-0.86 (m, 12H). ^{13}C NMR (101 MHz, $CDCl_3$): δ 157.88, 136.74, 126.35, 112.59, 72.94, 54.30, 49.42, 23.58, 22.03, 17.08, 16.72, 16.63, 16.47, 11.69, 11.52, -20.66. IR (neat) cm^{-1} ṽ: 2951, 2866, 1612, 1511, 1463, 1366, 1299, 1246, 1172, 1059, 1003, 882, 859, 831, 725, 683, 555, 520. HRMS (EI, 70eV) m/z calcd. for $C_{19}H_{33}IO2Si$ $[M]^+$: 448.1289; found: 448.1295.

(iodomethyl)diisopropyl(3-methyl-1-(4-(trifluoromethyl)phenyl)butoxy)silane

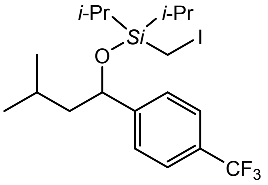

1p

1p was prepared according to general procedure in 80% isolated yield. Colorless oil. ^{1}H NMR (400 MHz, $CDCl_3$): δ 7.58 (d, J = 8.0 Hz, 2H), 7.44 (d, J = 8.0 Hz, 2H), 4.95 (t, J = 6.3 Hz, 1H), 1.92 (s, 2H), 1.78-1.69 (m, 1H), 1.60 -1.43(m, 2H), 1.30-1.04 (m, 8H), 1.02– 0.87 (m, 12H). ^{13}C NMR (101 MHz, $CDCl_3$): δ 148.58, 128.62 (q, J = 32.3 Hz), 125.46, 125.39, 124.32 (q, J = 4.0 Hz), 123.75 (q, J = 272.7 Hz), 109.17, 72.84, 49.41, 23.48, 22.07, 22.04, 16.95, 16.70, 16.57, 16.45, 11.67, 11.53. ^{19}F NMR (376 MHz, $CDCl_3$): δ -62.37 (s, 1F). IR (neat) cm^{-1} ṽ: 2954, 2868, 1620, 1464, 1323, 1164, 1125, 1063, 1013, 882, 861, 839, 727, 606. HRMS (EI, 70eV) m/z calcd. for $C_{19}H_{30}F_3IOSi$ $[M]^+$: 486.1057; found: 486.1061.

4-(1-(((iodomethyl)diisopropylsilyl)oxy)-3-methylbutyl)benzonitrile

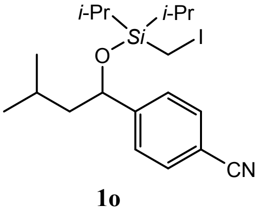

1o

1o was prepared according to general procedure in 88% isolated yield. Colorless oil. ^{1}H NMR (400 MHz, CDCl3): δ 7.61 (d, J = 8.2 Hz, 2H), 7.43 (d, J = 8.1 Hz, 2H), 4.93 (t, J = 6.3 Hz, 1H), 1.90 (s, 2H), 1.75-1.64 (m, 1H), 1.57-1.40 (m, 2H), 1.28-1.01(m, 8H), 1.00-0.84 (m, 12H). ^{13}C NMR (101 MHz, CDCl3): δ 150.91, 132.24, 126.86, 118.98, 111.18, 73.71, 50.19, 24.41, 23.05, 23.00, 17.86, 17.65, 17.52, 17.41, 12.60, 12.49. IR (neat) cm^{-1} ṽ: 2952, 2866, 2228, 1609, 1503, 1463, 1384, 1367, 1081, 1005, 901, 881, 861, 837, 816, 727, 864, 570. HRMS (EI, 70eV) m/z calcd. for $C_{19}H_{30}INOSi$ [M]$^+$: 443.1136; found: 443.1138.

(iodomethyl)diisopropyl(3-methyl-1-(4-(trifluoromethoxy)phenyl)butoxy)silane

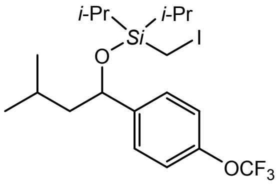

1q

1q was prepared according to general procedure in 87% isolated yield. Colorless oil. ^{1}H NMR (400 MHz, $CDCl_3$): δ 7.35 (d, *J* = 8.6 Hz, 2H), 7.16 (d, *J* = 8.1 Hz, 2H), 4.90 (t, *J* = 6.6 Hz, 1H), 1.92 (s, 2H), 1.78-1.69 (m, 1H), 1.60-1.52 (m, 1H), 1.51-1.42 (m, 1H), 1.25-1.03 (m, 8H), 1.01-0.87 (m, 12H). ^{13}C NMR (101 MHz, $CDCl_3$): δ 147.48, 147.46, 126.54, 119.79, 119.65 (q, *J* = 272.7 Hz), 72.67, 49.44, 23.52, 22.05, 22.02, 16.97, 16.65, 16.57, 16.43, 11.70, 11.56, -21.32. ^{19}F NMR (376 MHz, $CDCl_3$): δ -57.94 (s, 1F). IR (neat) cm^{-1} ṽ: 2954, 2868, 1508, 1464, 1368, 1255, 1219, 1163, 1081, 1007, 881, 861, 726, 680, 559. HRMS (ESI) m/z calc. for $C_{19}H_{30}F_3INaO_2Si$ [M+Na]$^+$: 525.0904; found: 525.0920.

(1-(2-bromo-6-fluorophenyl)-3-methylbutoxy)(iodomethyl)diisopropylsilane

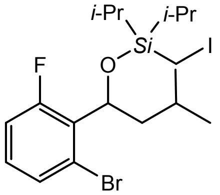

1r

1r was prepared according to general procedure in 65% isolated yield. Colorless oil. ^{1}H NMR (400 MHz, $CDCl_3$): δ 7.42 (dd, *J* = 8.7, 5.3 Hz, 1H), 7.28 (d, *J* = 3.1 Hz, 1H), 6.84 (td, *J* = 8.4, 3.1 Hz, 1H), 5.20 (dd, *J* = 8.3, 3.3 Hz, 1H), 1.95 (s, 2H), 1.84-1.73 (m, 1H), 1.61-1.54 (m, 1H), 1.44-1.35 (m, 1H), 1.30-1.06 (m, 8H), 1.03-0.90 (m, 12H). ^{13}C NMR (101 MHz, $CDCl_3$): δ 161.43 (d, *J* = 247.89 Hz), 146.49 (d, *J* = 7.0 Hz), 132.70 (d, *J* = 8.0 Hz), 115.02 (d, *J* = 22.9 Hz), 114.19 (d, *J* = 23.7 Hz), 109.16, 71.59, 48.18, 23.60, 23.03, 21.26, 16.99, 16.77, 16.55, 16.38, 11.51, 11.43, -21.62. ^{19}F NMR (376 MHz, $CDCl_3$): δ -114.02 (dd, *J* = 12.7, 7.7 Hz). IR (neat) cm^{-1} ṽ: 2953, 2867, 1580, 1463, 1407, 1366, 1256, 1154, 1084, 1006, 900, 882, 808, 765, 729, 629,

585, 482. HRMS (EI, 70eV) m/z calcd. for $C_{18}H_{29}BrFIOSi$ $[M]^+$: 514.0194; found:514.0201.

3. Experimental Details

3.1 Optimization of the reaction conditions

Table S1 Ligand Screening[a]

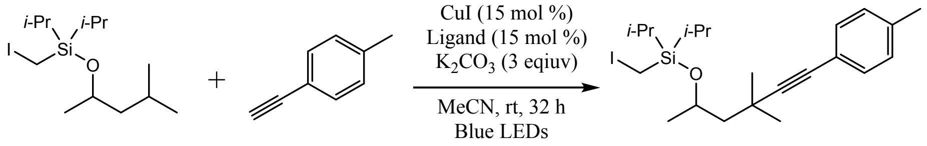

Entry	Ligand	yield%[b]
1	**L1**	62/60[c]
2	**L2**	0
3	**L3**	41
4	**L4**	0

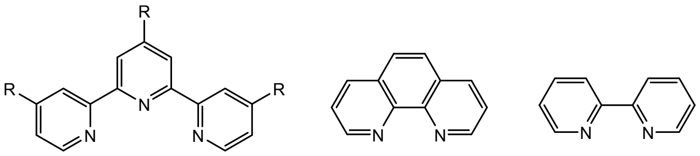

R = H, **L1**
R = *t*-Bu, **L2**
L3
L4

[a] **1a** (0.1 mmol), **2a** (0.15 mmol), CuI (15 mol %), Ligand (15 mol %) and K_2CO_3 (3 equiv.) in MeCN, under N_2, rt, blue LEDs, 32 h. [b] Determined by ^1H NMR analysis with internal standard (diethyl phthalate). [c] Isolated yield.

Table S2 Catalyst Screening[a]

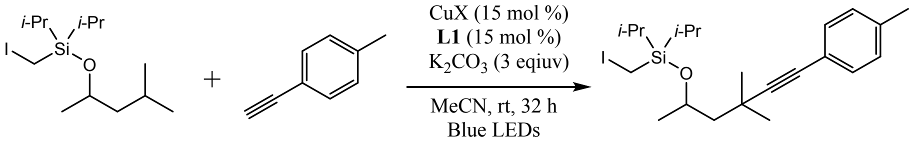

Entry	CuX	yield%[b]
1	CuCl	50
2	CuBr	38
3	$Cu(MeCN)_4PF_6$	36
4	CuTc	52
5	$Cu(OTf)_2$	32
6	CuCN	30

7[c]	$Pd(OAc)_2$	0

[a] **1a** (0.1 mmol), **2a** (0.15 mmol), CuX (15 mol %), **L1** (15 mol %) and K_2CO_3 (3 equiv.) in MeCN, under N_2, rt, blue LEDs, 32 h. [b] Determined by ^1H NMR analysis with internal standard (diethyl phthalate). [c] $Pd(OAc)_2$ (10 mol %), Xanphos (20 mol %) and Cs_2CO_3 (2 equiv.) in PhH, under N_2, rt, blue LEDs, 32 h.

Table S3 Solvent Screening[a]

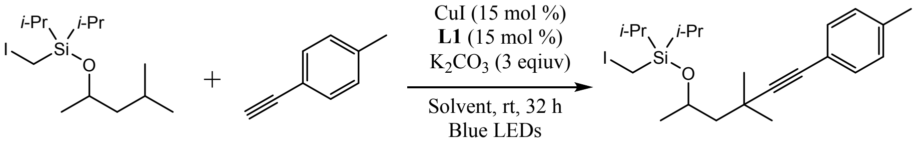

Entry	Solvent	yield%[b]
1	DMF	35
2	THF	Trace
3	Et_2O	Trace
4	PhH	0
5	DCM	38
6	DCE	0

[a] **1a** (0.1 mmol), **2a** (0.15 mmol), CuI (15 mol %), **L1** (15 mol %) and K_2CO_3 (3 equiv.) in solvent, under N_2, rt, blue LEDs, 32 h. [b] Determined by ^1H NMR analysis with internal standard (diethyl phthalate).

Table S4 Base Screening[a]

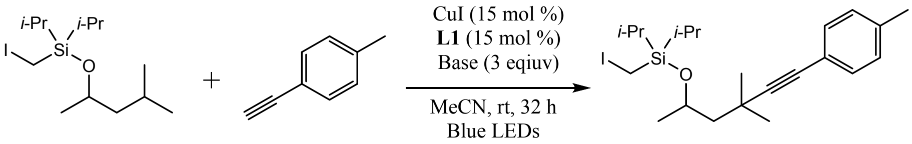

Entry	Base	yield%[b]
1	Na_2CO_3	0
2	Cs_2CO_3	0
3	K_3PO_4	39
4	KO*t*Bu	0

[a] **1a** (0.1 mmol), **2a** (0.15 mmol), CuI (15 mol %), **L1** (15 mol %) and base (3 equiv.) in solvent, under N_2, rt, blue LEDs, 32 h. [b] Determined by ^1H NMR analysis with internal standard (diethyl phthalate).

Table S4 Temperature Screening[a]

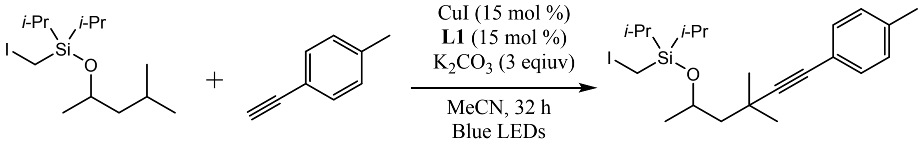

Entry	Temperature	yield%[b]
1	10 ℃	20

[a] **1a** (0.1 mmol), **2a** (0.15 mmol), CuI (15 mol %), L1 (15 mol %) and K_2CO_3 (3 equiv.) in solvent, under N_2, 10°C, blue LEDs, 32 h. [b] Determined by ^1H NMR analysis with internal standard (diethyl phthalate).

3.2 Genernal procedures and characterizations of products

Genernal procedure A

In a dried sealed vial, **L1** (0.015 mmol, 15 mol %), CuI (0.015 mmol, 15 mol %), K_2CO_3 (0.3 mmol, 3.0 equiv.) and terminal alkyne (0.15 mmol, 1.5 equiv.) were dissolved in anhydrous CH_3CN (2.0 mL) under a N_2 atmosphere, and the mixture was stirred for 30 min. Then tethered alcohols (0.1 mmol, 1.0 equiv.) were added. The tube was sealed with Teflon septum, the reaction mixture was stirred under nitrogen at room temperature under Blue LED for 32 h. The vial distance from the lamp was about 2-3 cm. The resulting mixture was passed through a pad of silica gel, and concentrated under a reduced pressure. The residual oil was purified by column chromatography in hexanes/EtOAc to afford protected products.

Genernal procedure B

In a dried sealed vial, **L1** (0.015 mmol, 15 mol %), CuI (0.015 mmol, 15 mol %), K_2CO_3 (0.3 mmol, 3.0 equiv.) and terminal alkyne (0.15 mmol, 1.5 equiv.) were dissolved in anhydrous CH_3CN (2.0 mL) under a N_2 atmosphere, and the mixture was stirred for 30 min. Then tethered alcohols (0.1 mmol, 1.0 equiv.) were added. The tube was sealed with Teflon septum, the reaction mixture was stirred under nitrogen at room temperature under Blue LED for 32 h. The vial distance from the lamp was about 2-3 cm. The resulting mixture was passed through a pad of silica gel, and concentrated under a reduced pressure. The residue oil was dissolved in THF (1.0 mL), and TBAF (2 mL, 1.0 M THF,) was added. After completion (monitored by TLC), the resulting mixture was poured into water with EtOAc. The aqueous layer was extracted with two portions of EtOAc. The combined extract was washed with brine, dried over Na_2SO_4 and concentrated. The residue oil was purified by column chromatography in hexanes/EtOAc to afford deprotected products.

Genernal procedure C

In a dried sealed vial, **L1** (0.015 mmol, 15 mol %), CuI (0.015 mmol, 15 mol %), K_2CO_3 (0.3 mmol, 3.0 equiv.) and terminal alkyne (0.15 mmol, 1.5 equiv.) were dissolved in anhydrous CH_3CN (2.0 mL) under a N_2 atmosphere, and the mixture was stirred for 30 min. Then tethered alcohols (0.1 mmol, 1.0 equiv.) were added. The tube was sealed with Teflon septum, the reaction mixture was stirred under nitrogen at room temperature under Blue LED for 32 h. The vial distance from the lamp was about 2-3 cm. The resulting mixture was passed through a pad of Celite, and concentrated under a reduced pressure. The residue was dissolved in CH_2Cl_2 (1.0 mL), and montmorillnotite K10 (30 mg) and acetyl chloride (71 μL 1.0 mmol) were added

sequentially. After completion (monitored by TLC), the resulting mixture was passed through a pad of silica gel, and concentrated under reduced pressure. The residue oil was purified by column chromatography in hexanes/EtOAc to afford *O*-acetyl alcohols.

Genernal procedure D

In a dried sealed vial, CuCl (0.015 mmol, 15 mol %), LiO^{t}Bu (0.3 mmol, 3.0 equiv.) and 9*H*-carbazole (0.15 mmol, 1.5 equiv.) were dissolved in anhydrous CH_3CN (2.0 mL) under a N_2 atmosphere, and the mixture was stirred for 30 min. Then tethered alcohols (0.1 mmol, 1.0 equiv.) were added. The tube was sealed with Teflon septum, the reaction mixture was stirred under nitrogen at room temperature under Blue LED for 24h. The vial distance from the lamp was about 2-3 cm. The resulting mixture was passed through a pad of silica gel, and concentrated under a reduced pressure. The residual oil was purified by column chromatography in hexanes/EtOAc to afford protected products.

Genernal procedure E

In a dried sealed vial, CuCl (0.015 mmol, 15 mol %), LiO^{t}Bu (0.3 mmol, 3.0 equiv.) and 9*H*-carbazole (0.15 mmol, 1.5 equiv.) were dissolved in anhydrous CH_3CN (2.0 mL) under a N_2 atmosphere, and the mixture was stirred for 30 min. Then tethered alcohols (0.1 mmol, 1.0 equiv.) were added. The tube was sealed with Teflon septum, the reaction mixture was stirred under nitrogen at room temperature under Blue LED for 24h. The vial distance from the lamp was about 2-3 cm. The residue oil was dissolved in THF (1.0 mL), and TBAF (2 mL, 1.0 M THF,) was added. After completion (monitored by TLC), the resulting mixture was poured into water with EtOAc. The aqueous layer was extracted with two portions of EtOAc. The combined extract was washed with brine, dried over Na_2SO_4 and concentrated. The residue oil was purified by column chromatography in hexanes/EtOAc to afford deprotected products.

3.3 Characterization data of products

((4,4-dimethyl-6-(p-tolyl)hex-5-yn-2-yl)oxy)diisopropyl(methyl)silane

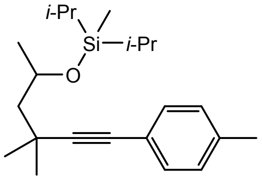

3a

3a was prepared according to general procedure **A** in 60% isolated yield. Colorless oil ^{1}H NMR (400 MHz, $CDCl_3$): δ 7.26 (d, *J* = 7.8 Hz, 2H), 7.08 (d, *J* = 7.8 Hz, 2H), 4.23-4.14 (m, 1H), 2.69(br, 1H), 2.33 (s, 1H), 1.76-1.62 (m, 2H), 1.35-1.28 (m, 9H), 1.06-0.85 (m, 14H), 0.05 (s, 3H). ^{13}C NMR (101 MHz, cdcl$_3$): δ 136.47, 130.44,

128.01, 120.13, 95.56, 80.06, 66.45, 51.99, 30.63, 29.62, 28.71, 24.61, 20.54, 16.74, 16.71, 16.66, 12.58, 12.55, -8.34. IR (neat) cm^{-1} ṽ: 2962, 2939, 2865, 1510, 1462, 1377, 1362, 1251, 1151, 1131, 1060, 1017, 995, 937, 882, 815, 779, 735, 641. HRMS (EI, 70eV) m/z calcd. for $C_{22}H_{36}OSi$ $[M]^+$: 344.2530; found:344.2531.

4,4-dimethyl-6-phenylhex-5-yn-2-ol

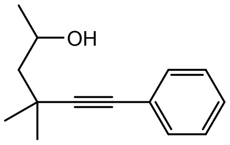

3b

3b was prepared according to general procedure **B** in 60% isolated yield. Colorless oil ^{1}H NMR (400 MHz, $CDCl_3$): δ 7.41-7.34 (m, 2H), 7.29-7.24 (m, 2H), 4.24-4.15 (m, 1H), 2.86 (br. s, 1H), 1.77 (dd, J = 14.2, 9.1 Hz, 1H), 1.53 (dd, J = 14.2, 1.4 Hz, 1H), 1.35 (d, J = 4.1 Hz, 6H), 1.23 (d, J = 6.2 Hz, 3H). ^{13}C NMR (101 MHz, $CDCl_3$): δ 131.64, 128.38, 128.09, 123.17, 96.64, 82.42, 65.96, 52.02, 31.23, 30.27, 29.12, 24.40. IR (neat) cm^{-1} ṽ: 3385, 2966, 2926, 1598, 1363, 1293, 1165, 1152, 1119, 1048, 910, 754, 690, 553, 466. HRMS (ESI) m/z calcd. for $C_{14}H_{19}O$ $[M+H]^+$: 203.1430; found: 203.1432.

6-([1,1'-biphenyl]-4-yl)-4,4-dimethylhex-5-yn-2-ol

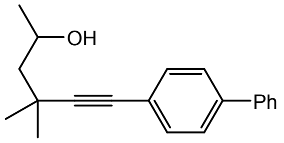

3c

3c was prepared according to general procedure **B** in 39% isolated yield. White solid. ^{1}H NMR (400 MHz, $CDCl_3$): δ 7.61-7.55 (m, 2H), 7.54-7.49 (m, 2H), 7.47-7.41 (m, 4H), 7.37-7.31 (m, 1H), 4.26-4.17 (m, 1H), 1.78 (dd, J = 14.3, 9.1 Hz, 1H), 1.55 (dd, J = 14.2, 2.1 Hz, 1H), 1.37 (d, J = 4.3 Hz, 6H), 1.25 (d, J = 6.3 Hz, 3H). ^{13}C NMR (101 MHz, $CDCl_3$): δ 140.85, 140.54, 132.07, 128.96, 127.70, 127.14, 127.07, 122.15, 97.37, 82.27, 66.02, 52.11, 31.23, 30.40, 29.21, 24.47. IR (neat) cm^{-1} ṽ: 3240, 2963, 2922, 1485, 1449, 1292, 1148, 1116, 1075, 916, 841, 760, 719, 689. HRMS (ESI) m/z calcd. for $C_{20}H_{23}O$ $[M+H]^+$: 279.1743; found: 279.1743.

6-(4-methoxyphenyl)-4,4-dimethylhex-5-yn-2-ol

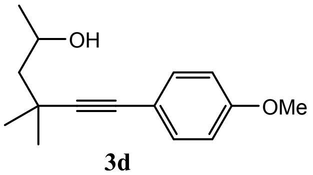

3d

3d was prepared according to general procedure **B** in 64% isolated yield. Colorless oil. ^{1}H NMR (400 MHz, $CDCl_3$): δ 7.31 (d, *J* = 8.6 Hz, 2H), 6.80 (d, *J* = 8.6 Hz, 2H), 4.23-4.14 (m, 1H), 3.79 (s, 3H), 3.02 (br. s, 1H), 1.75 (dd, *J* = 14.2, 9.2 Hz, 1H), 1.51(dd, *J* = 14.1, 1.7 Hz, 1H), 1.33 (d, *J* = 3.0 Hz, 6H), 1.22 (d, *J* = 6.2 Hz, 3H). ^{13}C NMR (101 MHz, $CDCl_3$): δ 158.46, 132.01, 114.26, 112.98, 94.07, 81.26, 64.93, 54.40, 51.07, 30.40, 29.20, 28.10, 23.34. IR (neat) cm^{-1} ṽ: 3406, 2965, 2927, 1606, 1508, 1463, 1284, 1245, 1167, 1107, 1031, 945, 911, 831, 764, 618, 531. HRMS (ESI) m/z calcd. for $C_{15}H_{21}O_2$ [M+H]$^+$: 233.1536; found: 233.1537.

6-(4-chlorophenyl)-4,4-dimethylhex-5-yn-2-ol

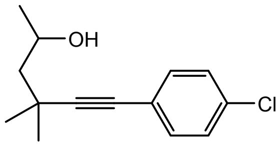

3e

3e was prepared according to general procedure **B** in 41% isolated yield. Colorless oil ^{1}H NMR (400 MHz, $CDCl_3$): δ 7.30 (d, *J* = 8.3 Hz, 2H), 7.25 (d, *J* = 8.3 Hz, 2H), 4.23-4.14 (m, 1H), 2.69 (br. s, 1H), 1.76 (dd, *J* = 14.4, 8.8 Hz, 1H), 1.53 (dd, *J* = 14.4, 2.0 Hz, 1H), 1.35(d, *J* = 4.8 Hz, 6H), 1.23 (d, *J* = 6.0 Hz, 3H). ^{13}C NMR (101 MHz, $CDCl_3$): δ 134.06, 132.88, 128.71, 121.72, 97.73, 81.28, 66.00, 51.95, 31.06, 30.36, 29.11), 24.47. IR (neat) cm^{-1} ṽ: 3405, 2967, 2927, 1490, 1466, 1261, 1089, 1048, 1015, 827, 799, 517. HRMS (EI, 70eV) m/z calc. for $C_{14}H_{17}ClO$ [M]$^+$: 236.0968; found: 236.0960.

6-(4-fluorophenyl)-4,4-dimethylhex-5-yn-2-ol

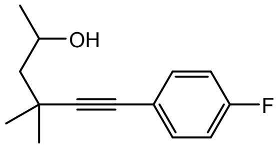

3f

3f was prepared according to general procedure **B** in 39% isolated yield. Orange oil. ^{1}H NMR (400 MHz, $CDCl_3$): δ 7.35 (dd, *J* = 8.5, 5.5Hz, 2H), 6.97 (t, *J* = 8.7 Hz, 2H), 4.23-4.14 (m, 1H), 2.79 (br. s, 1H), 1.75 (dd, *J* = 14.2, 9.1 Hz, 1H), 1.53 (dd, *J* = 14.2, 2.1Hz, 1H), 1.34 (d, *J* = 4.4 Hz, 6H), 1.23 (d, *J* = 6.2 Hz, 3H). ^{13}C NMR (101 MHz, $CDCl_3$): δ 161.42 (d, *J* = 249.9 Hz), 132.47 (d, *J* = 8.4 Hz), 118.25 (d, *J* = 3.5 Hz), 114.62 (d, *J* = 22.1 Hz), 95.33, 80.33, 64.99, 50.98, 30.16, 29.27, 28.11, 23.44. ^{19}F NMR (376 MHz, $CDCl_3$): δ -111.63--111.70(m, 1F). IR (neat) cm^{-1} ṽ: 3418, 2968, 2928, 1601, 1506, 1467, 1230, 1156, 1093, 1049, 835, 525. HRMS (ESI) m/z calcd. for $C_{14}H_{18}FO$ [M+H]$^+$: 221.1336; found: 221.1338.

6-(4-(dimethylamino)phenyl)-4,4-dimethylhex-5-yn-2-yl acetate

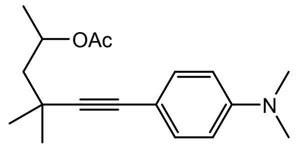

3g

3g was prepared according to general procedure **C** in 72% isolated yield. Orange oil. ^{1}H NMR (400 MHz, $CDCl_3$): δ 7.25 (d, *J* = 8.2 Hz, 2H), 6.60 (d, *J* = 8.2 Hz, 2H), 5.31-5.21 (s, 1H), 2.94 (s, 6H), 1.98 (s, 3H), 1.86 (dd, *J* = 14.2, 8.0 Hz, 1H), 1.63 (dd, *J* = 14.0, 2.8 Hz, 1H), 1.30 (s, 9H). ^{13}C NMR (101 MHz, $CDCl_3$): δ 170.81, 149.80, 132.63, 112.08, 111.22, 93.84, 81.47, 69.33, 48.89, 40.50, 30.69, 30.59, 29.86, 21.78, 21.75. IR (neat) cm^{-1} ṽ: 2969, 2926, 1734, 1609, 1520, 1445, 1359, 1243, 1163, 1130, 1045, 1017, 946, 817. HRMS (ESI) m/z calcd. for $C_{16}H_{24}NO$ $[M+H]^+$: 288.1958; found: 288.1957.

4,4-dimethyl-6-(m-tolyl)hex-5-yn-2-ol

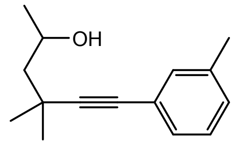

3h

3h was prepared according to general procedure **B** in 55% isolated yield. Orange oil. ^{1}H NMR (400 MHz, $CDCl_3$): δ 7.24-7.13 (m, 3H), 7.12-7.06 (m, 1H), 4.27-4.11 (m, 1H), 2.96 (s, 3H), 2.30 (s, 1H), 1.76 (dd, *J* = 14.2, 9.2 Hz, 1H), 1.52 (dd, *J* = 14.2, 1.8 Hz, 1H), 1.35 (d, *J* = 3.7 Hz, 6H), 1.23 (d, *J* = 6.2 Hz, 3H). ^{13}C NMR (101 MHz, $CDCl_3$): δ 138.07, 132.23, 128.98, 128.67, 128.28, 122.92, 96.21, 82.59, 65.94, 52.02, 31.28, 30.22, 29.08, 24.37, 21.29. IR (neat) cm^{-1} ṽ: 3395, 2966, 2925, 1601, 1453, 1365, 1301, 1153, 1119, 1047, 782, 691. HRMS (ESI) m/z calcd. for $C_{15}H_{21}O$ $[M+H]^+$: 217.1587; found: 217.1586.

6-(2-fluorophenyl)-4,4-dimethylhex-5-yn-2-ol

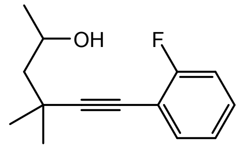

3i

3i was prepared according to general procedure **B** in 45% isolated yield. Colorless oil. ^{1}H NMR (400 MHz, $CDCl_3$): δ 7.40-7.33 (m, 1H), 7.28-7.21 (m, 1H), 7.09-6.99 (m, 2H), 4.27-4.14 (m, 1H), 1.78 (dd, *J* = 14.2, 9.0 Hz, 1H), 1.54 (dd, *J* = 14.4, 2.1 Hz, 1H), 1.36 (d, *J* = 5.4 Hz, 6H), 1.23 (d, *J* = 6.3 Hz, 3H). ^{13}C NMR (101 MHz, $CDCl_3$): δ 162.87 (d, *J* = 251.4 Hz), 133.42 (d, *J* = 1.1 Hz), 129.69 (d, *J* = 8.0 Hz), 123.97 (d, *J* = 3.7 Hz), 115.49 (d, *J* = 21.2 Hz), 111.84 (d, *J* = 15.7 Hz), 102.18 (d, *J* = 3.4 Hz), 75.59, 65.87, 52.05, 31.06, 30.56, 29.10, 24.46. ^{19}F NMR (376 MHz, $CDCl_3$): δ -108.57--112.73 (m, 1F). IR (neat) cm^{-1} ṽ: 3391, 2968, 2928, 1492, 1451, 1264, 1216,

1152, 1102, 1048, 913, 826, 754. HRMS (ESI) m/z calcd. for $C_{14}H_{18}FO$ $[M+H]^+$: 221.1336; found: 221.1336.

4,4-dimethyl-6-(pyridin-3-yl)hex-5-yn-2-ol

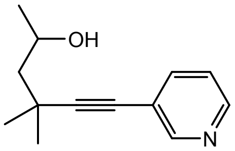

3j

3j was prepared according to general procedure **B** in 53% isolated yield. Colorless oil. ^{1}H NMR (400 MHz, $CDCl_3$): δ ^{1}H NMR (400 MHz, $CDCl_3$) δ 8.60 (s, 1H), 8.47 (s, 1H), 7.64 (d, *J* = 7.7 Hz, 1H), 7.23-7.16 (m, 1H), 4.23-4.13 (m, 1H), 2.59 (s, 1H), 1.75 (dd, *J* = 14.0, 8.9 Hz, 1H), 1.56 (d, *J* = 14.2 Hz, 1H), 1.35 (d, *J* = 7.7 Hz, 6H), 1.23 (d, *J* = 6.1 Hz, 3H). ^{13}C NMR (101 MHz, $CDCl_3$): δ 152.28, 148.37, 138.56, 123.04, 120.48, 100.42, 78.84, 65.98, 51.85, 30.72, 30.56, 29.31, 24.65. IR (neat) cm^{-1} ṽ: 3344.68, 2965.60, 2926.05, 2870.57, 2227.26, 1563.85, 1470.78, 1406.76, 1365.42, 1294.31, 1186.77, 1150.45, 1120.76, 1073.82, 1047.31, 1024.32, 947.14, 910.86, 804.59, 704.02, 628.93, 467.85. HRMS (ESI) m/z calcd. for $C_{13}H_{18}NO$ $[M]^+$: 204.1383; found: 204.1384.

3,3-dimethyl-5-phenylpent-4-yn-2-ol

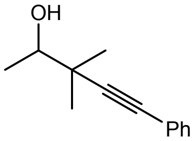

4a

4a was prepared according to general procedure **B** in 30% isolated yield. Colorless oil. ^{1}H NMR (400 MHz, $CDCl_3$): δ 7.43-7.37 (m, 2H), 7.31-7.27 (m, 3H), 3.67-3.60 (m, 1H), 1.82 (s, 1H), 1.32 (s, 3H), 1.28 (d, *J* = 6.3 Hz, 3H), 1.26 (s, 3H). ^{13}C NMR (101 MHz, $CDCl_3$): δ 131.80, 128.36, 128.00, 123.50, 94.36, 82.69, 74.41, 38.30, 25.70, 24.77, 18.31. IR (neat) cm^{-1} ṽ: 3393, 2972, 2925, 2855, 1670, 1599, 1490, 1444, 1375, 1274, 1076, 1025, 912, 755, 691, 544. HRMS (ESI) m/z calcd. for $C_{13}H_{16}O$ $[M]^+$: 189.1274; found: 189.1274.

5,5-dimethyl-7-phenylhept-6-yn-2-ol

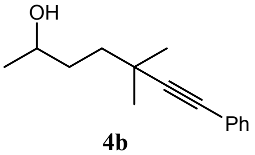

4b

4b was prepared according to general procedure B in 41% isolated yield. Colorless oil. 1H NMR (400 MHz, $CDCl_3$): δ 7.40-7.35 (m, 2H), 7.30-7.24 (m, 3H), 3.89-3.80 (m, 1H), 1.74-1.59 (m, 3H), 1.54-1.45 (m, 1H), 1.29 (d, J = 1.6 Hz, 6H), 1.24 (d, J = 6.2 Hz, 3H). 13C NMR (101 MHz, $CDCl_3$): δ 130.69, 127.27, 126.61, 123.07, 96.15,

79.71, 67.65, 38.56, 34.35, 30.58, 28.56, 28.28, 22.75. IR (neat) cm^{-1} ṽ: 3366, 2966, 2930, 2867, 1598, 1490, 1447, 1367, 1261, 1115, 1068, 799, 755, 691. HRMS (ESI) m/z calcd. for $C_{15}H_{21}O$ [M+H]$^+$: 217.1587; found: 217.1588.

2,5,5-trimethyl-7-phenylhept-6-yn-3-ol

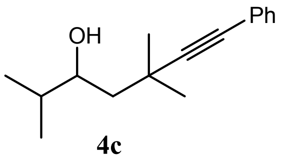

4c

4c was prepared according to general procedure **B** in 61% isolated yield. Colorless oil. ^{1}H NMR (400 MHz, $CDCl_3$): δ 7.40-7.35 (m, 2H), 7.31-7.25 (m, 3H), 3.78 (ddd, *J* = 8.9, 4.8, 1.6 Hz, 1H), 2.07 (s, 1H), 1.74-1.63 (m, 3H), 1.57 (dd, *J* = 14.2, 1.7 Hz, 1H), 1.36 (d, *J* = 13.9 Hz, 6H), 0.94 (dd, *J* = 6.8, 2.6 Hz, 6H). ^{13}C NMR (101 MHz, $CDCl_3$): δ 131.67, 128.36, 128.04, 123.26, 96.93, 82.20, 74.16, 47.38, 34.35, 31.19, 30.39, 29.07, 18.52, 17.47. IR (neat) cm^{-1} ṽ: 3454, 2963, 1598, 1490, 1466, 1364, 1143, 1031, 998, 914, 754, 690. HRMS (ESI) m/z calcd. for $C_{16}H_{23}O$ [M+H]$^+$: 231.1743; found: 231.1744.

3,3,8-trimethyl-1-phenylnon-1-yn-5-ol

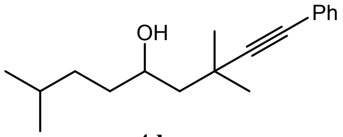

4d

4d was prepared according to general procedure **B** in 60% isolated yield. Colorless oil. ^{1}H NMR (400 MHz, $CDCl_3$): δ 7.41-7.34 (m, 1H), 7.30-23 (m, 2H), 4.01-3.92 (m, 1H), 1.73 (dd, *J* = 14.2, 9.2 Hz, 1H), 1.64-1.11 (m, 12H), 0.90 (dd, *J* = 6.5, 1.3 Hz, 6H). ^{13}C NMR (101 MHz, $CDCl_3$): δ 131.65, 128.36, 128.07, 123.18, 96.81, 82.35, 70.07, 50.50, 36.19, 34.79, 31.27, 30.30, 29.07, 28.27, 22.81, 22.71. IR (neat) cm^{-1} ṽ: 3425, 2956, 2929, 2869, 1491, 1467, 1384, 1364, 1296, 1164, 1068, 1029, 1010, 913, 755, 691. HRMS (ESI) m/z calcd. for $C_{18}H_{27}O$ [M+H]$^+$: 259.2056; found: 259.2056.

5,5-dimethyl-7-phenylhept-6-yn-3-ol

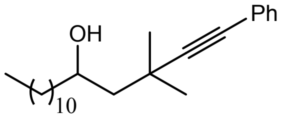

4e

4e was prepared according to general procedure **B** in 64% isolated yield. Colorless oil. ^{1}H NMR (400 MHz, $CDCl_3$): δ 7.39-7.35 (m, 2H), 7.30-7.24 (m, 3H), 4.02-3.95 (m, 1H), 2.83 (s, 1H), 1.72 (dd, *J* = 14.2, 9.1 Hz, 1H), 1.55 (d, *J* = 14.4 Hz, 1H), 1.48-1.39 (m, 2H), 1.39-1.18 (m, 20H), 0.88 (t, *J* = 6.7 Hz, 3H). ^{13}C NMR (101 MHz, $CDCl_3$): δ 131.66, 128.36, 128.06, 123.21, 96.83, 82.33, 69.77, 50.52, 38.40, 32.07, 31.26, 30.33, 29.85, 29.82, 29.80, 29.78, 29.50, 29.11, 25.69, 22.84, 14.27. IR (neat) cm^{-1} ṽ: 3410,

2923, 2853, 1465, 1362, 1295, 754, 690. HRMS (ESI) m/z calcd. for $C_{24}H_{39}O$ $[M+H]^+$: 343.2995; found: 343.2994.

3,3-dimethyl-5-phenylpent-4-yn-1-ol

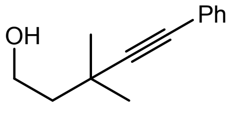

4f

4f was prepared according to general procedure **B** in 31% isolated yield. Orange oil. ^1H NMR (400 MHz, $CDCl_3$): δ 7.39-7.35 (m, 2H), 7.30-7.25 (m, 3H), 3.92 (t, *J* = 6.7 Hz, 2H), 1.81 (t, *J* = 6.7 Hz, 2H), 1.71 (s, 1H), 1.34 (s, 6H). ^{13}C NMR (101 MHz, $CDCl_3$): δ 131.68, 128.35, 127.90, 123.59, 96.65, 81.43, 60.80, 60.80, 45.72, 30.21, 29.90. IR (neat) cm^{-1} ṽ: 3330, 2967, 2927, 1598, 1490, 1444, 1363, 1312, 1063, 1026, 989, 754, 691, 552. HRMS (ESI) m/z calcd. for $C_{13}H_{17}O$ $[M+H]^+$: 189.1274; found: 189.1273.

6-methyl-8-phenyloct-7-yn-4-ol

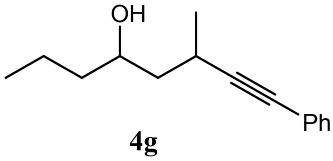

4g

4g was prepared according to general procedure **B** in 66% isolated yield. Colorless oil. ^1H NMR (400 MHz, $CDCl_3$): δ 7.43-7.33 (m, 3.8H), 7.29-7.24 (m, 5.7H), 4.03-3.89 (m, 1H), 3.88-3.80(m, 0.9H), 3.00-2.87 (m, 1H), 2.85-2.75 (m, 0.9H), 1.92 (s, 1.8H), 1.77-1.34 (m, 11.4H), 1.29 (d, *J* = 6.9 Hz, 5.7H), 0.94 (t, *J* = 6.9 Hz, 5.7H). ^{13}C NMR (101 MHz, $CDCl_3$): δ 131.68, 131.64, 128.32, 128.30, 127.87, 127.75, 123.85, 123.59, 94.33, 94.02, 81.76, 81.60, 70.69, 69.95, 44.59, 44.57, 40.18, 39.75, 24.17, 23.46, 21.69, 21.41, 19.00, 18.79, 14.22. IR (neat) cm^{-1} ṽ: 3345, 2959, 2931, 2871, 1598, 1489, 1454, 1089, 1022, 946, 843, 754, 690. HRMS (ESI) m/z calcd. for $C_{15}H_{21}O$ $[M+H]^+$: 217.1587; found: 217.1587.

6-methyl-8-phenyl-4-propyloct-7-yn-4-ol

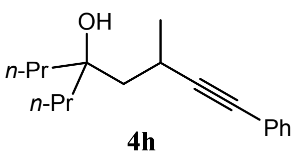

4h

4h was prepared according to general procedure **B** in 71% isolated yield. Colorless oil. ^1H NMR (400 MHz, $CDCl_3$): δ 7.40-7.34 (m, 2H), 7.30-7.22 (m, 3H), 2.89-2.74 (m, 1H), 1.79 (dd, *J* = 14.2, 10.8 Hz, 1H), 1.67-1.15 (m, 12H), 0.92 (dd, *J* = 15.6, 7.2 Hz, 6H). ^{13}C NMR (101 MHz, $CDCl_3$): δ 131.48, 128.24, 127.85, 123.35, 94.53, 82.28, 74.59, 45.96, 42.02, 41.57, 22.94, 21.62, 17.29, 16.74, 14.79, 14.74. IR (neat) cm^{-1} ṽ: 3452, 2958, 2931, 2871, 1598, 1490, 1454, 1376, 1137, 1069, 985, 911, 860, 754, 691, 545. HRMS (ESI) m/z calcd. for $C_{18}H_{27}O$ $[M+H]^+$: 259.2056; found: 259.2056.

1-cyclopropyl-3,3-dimethyl-5-phenylpent-4-yn-1-ol

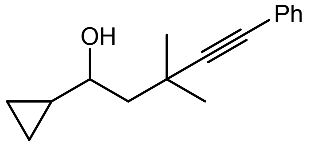

4i

4i was prepared according to general procedure **B** in 58% isolated yield. Colorless oil. ^{1}H NMR (400 MHz, $CDCl_3$): δ 7.39-7.32 (m, 2H), 7.30-7.23 (m, 3H), 3.25 (t, *J* = 8.5 Hz, 1H), 2.92-2.52 (br.s, 1H), 1.92-197 (m, 1H), 1.35 (d, *J* = 16.7 Hz, 3H), 1.03-0.91 (m, 1H), 0.60 – 0.36 (m, 3H), 0.26-0.16 (m, 1H). ^{13}C NMR (101 MHz, $CDCl_3$): δ 131.61, 128.34, 127.98, 123.29, 96.87, 82.08, 74.80, 50.31, 30.82, 30.41, 29.28, 18.68, 3.54, 2.45. IR (neat) cm^{-1} ṽ: 3418, 2967, 2927, 1598, 1490, 1443, 1362, 1290, 1069, 1030, 912, 754, 690, 529. HRMS (ESI) m/z calcd. for $C_{16}H_{21}O$ $[M+H]^+$: 229.1587; found: 229.1586.

4-ethoxy-6-phenylhex-5-yn-2-ol

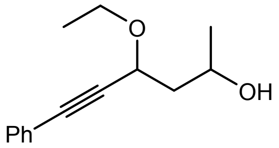

4j

4j was prepared according to general procedure **B** in 71% isolated yield. Colorless oil. ^{1}H NMR (400 MHz, $CDCl_3$): δ 7.48-7.41 (m, 4H), 7.36-7.25 (m, 6H), 4.60-4.55 (m, 1H), 4.55-4.45 (m, 1H), 4.37-4.26 (m, 1H), 4.15-4.05 (m, 1H), 4.0-3.87 (m, 1H), 3.59-3.46 (m, 1H), 2.11-1.86 (m, 4H), 1.38-1.12 (m, 12H). ^{13}C NMR (101 MHz, $CDCl_3$): δ 131.83, 131.82, 128.58, 128.55, 128.40, 122.62, 122.56, 87.62, 86.32, 86.00, 69.90, 68.52, 67.25, 65.20, 64.95, 64.80, 44.62, 43.82, 23.57, 23.35, 15.26, 15.24. IR (neat) cm^{-1} ṽ: 3405, 2971, 2927, 2871, 1489, 1443, 1371, 1329, 1080, 952, 755, 690, 594, 527. HRMS (ESI) m/z calcd. for $C_{14}H_{18}NaO_2$ $[M+Na]^+$: 241.1199; found: 241.1199.

(E)-7-phenylhept-3-en-6-yn-1-ol

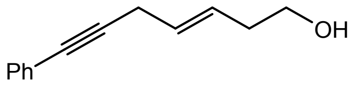

4k

4k was prepared according to general procedure **B** in 61% isolated yield. Colorless oil. ^{1}H NMR (400 MHz, $CDCl_3$): δ 7.47-7.37 (m, 2H), 7.34-7.27 (m, 3H), 5.82-5.72 (m, 1H), 5.68-5.59 (m, 1H), 3.69 (t, *J* = 6.3 Hz, 2H), 3.17 (d, *J* = 5.1 Hz, 2H), 2.38-2.30 (m, 2H).^{13}C NMR (101 MHz, $CDCl_3$): δ 131.54, 128.18, 128.02, 127.73, 127.33, 123.61, 87.05, 82.49, 61.87), 35.67, 22.68. IR (neat) cm^{-1} ṽ: 3341, 2924, 2881, 1490, 1442, 1419, 1260, 1043, 968, 798, 756, 691. HRMS (ESI) m/z calcd. for $C_{13}H_{14}NaO$ $[M+Na]^+$: 209.0937; found: 209.0939.

((3,5-diphenylpent-4-yn-1-yl)oxy)diisopropyl(methyl)silane

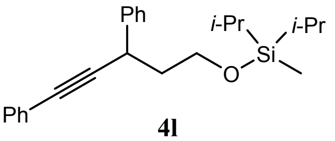

4l

4l was prepared according to general procedure **B** in 65% isolated yield. Colorless oil. ^{1}H NMR (400 MHz, $CDCl_3$): δ 7.47-7.4 0(m, 4H), 7.36-7.21 (m, 6H), 4.09 (t, J = 7.5 Hz, 1H), 3.94-3.86 (m, 1H), 3.78-3.70 (m, 1H), 2.05-1.98 (m, 2H), 1.09-0.89 (m, 14H), 0.04 (s, 3H). ^{13}C NMR (101 MHz, $CDCl_3$): δ 142.01, 131.65, 128.54, 128.23, 127.76, 127.58, 123.82, 91.30, 83.31, 60.82, 41.70, 34.55, 17.50, 17.49, 17.45, 13.06, 13.04, -8.61. IR (neat) cm^{-1} ṽ: 2941, 2864, 1599, 1490, 1458, 1384, 1251, 1002, 994, 941, 881, 781, 751, 692, 639, 544. HRMS (EI) m/z calcd. for $C_{24}H_{32}OSi$ $[M]^+$: 364.2217; found: 364.2223.

3,3-dimethyl-1,5-diphenylpent-4-yn-1-ol

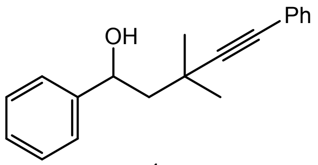

4m

4m was prepared according to general procedure **B** in 49% isolated yield. Colorless oil. ^{1}H NMR (400 MHz, $CDCl_3$): δ 7.46-7.37 (m, 4H), 7.34 (t, J = 7.5 Hz, 2H), 7.31-7.21 (m, 4H), 5.11 (dd, J = 9.4, 1.7 Hz, 1H), 2.06 (dd, J = 14.3, 9.4 Hz, 1H), 1.79 (dd, J = 14.3, 2.3 Hz, 1H), 1.40 (d, J = 4.3 Hz, 6H). ^{13}C NMR (101 MHz, $CDCl_3$): δ 145.26 (s), 131.70, 128.57, 128.40, 128.11, 127.46, 125.86, 123.23, 96.56, 82.59, 72.58, 52.76, 31.01, 30.81, 29.30. IR (neat) cm^{-1} ṽ: 3235, 2966, 2927, 1598, 1491, 1452, 1283, 1053, 1023, 911, 754, 694, 539. HRMS (ESI) m/z calcd. for $C_{19}H_{20}O$ $[M+H]^+$: 364.2217; found: 364.2223.

1-(4-methoxyphenyl)-3,3-dimethyl-5-phenylpent-4-yn-1-yl acetate

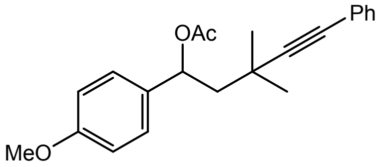

4n

4n was prepared according to general procedure **C** in 40% isolated yield. Colorless oil. ^{1}H NMR (400 MHz, $CDCl_3$): δ 7.39-7.35 (m, 2H), 7.31 (d, J = 8.7 Hz, 2H), 7.28-7.25 (m, 3H), 6.10 (dd, J = 8.5, 4.1 Hz, 1H), 3.77 (s, 3H), 2.21 (dd, J = 14.3, 8.7 Hz, 1H), 1.98 (s, 3H), 1.90 (dd, J = 14.4, 4.0 Hz, 1H), 1.36 (s, 3H), 1.28 (s, 3H). ^{13}C NMR (101 MHz, $CDCl_3$): δ 170.36, 159.34, 134.02, 131.74, 128.27, 128.13, 127.69, 123.94, 113.97, 96.11, 81.47, 73.97, 55.38, 48.99, 30.93, 30.24, 30.10, 21.62. IR (neat) cm^{-1} ṽ:

2966, 2933, 1735, 1612, 1513, 1369, 1234, 1176, 1028, 831, 757, 693, 608, 544. HRMS (ESI) m/z calcd. for $C_{22}H_{24}NaO_3$ $[M+Na]^+$: 359.1618; found: 359.1620.

1-(4-cyanophenyl)-3,3-dimethyl-5-phenylpent-4-yn-1-yl acetate

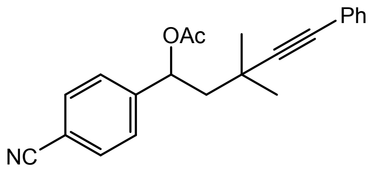

4o

4o was prepared according to general procedure **C** in 60% isolated yield. Colorless oil. ^{1}H NMR (400 MHz, $CDCl_3$): δ 7.62 (d, J = 8.3 Hz, 2H), 7.46 (d, J = 8.3 Hz, 2H), 7.38-7.32 (m, 1H), 7.32-7.27 (m, 3H), 6.13 (dd, J = 8.8, 3.5 Hz, 1H), 2.17 (dd, J = 14.5, 8.8 Hz, 1H), 2.03 (s, 1H), 1.88 (dd, J = 14.4, 3.6 Hz, 1H), 1.38 (s, 3H), 1.32 (s, 3H). ^{13}C NMR (101 MHz, $CDCl_3$): δ 170.15, 147.14, 132.56, 131.66, 128.39, 127.97, 127.25, 123.55, 118.75, 111.80, 95.45, 81.97, 73.69, 49.06, 31.12, 30.47, 29.83, 21.36. IR (neat) cm^{-1} ṽ: 2969, 2923, 2229, 1739, 1371, 1233, 1031, 1019, 835, 758, 693, 566. HRMS (ESI) m/z calcd. for $C_{22}H_{21}NNaO_2$ $[M+Na]^+$: 354.1465; found: 354.1468.

3,3-dimethyl-5-phenyl-1-(4-(trifluoromethyl)phenyl)pent-4-yn-1-ol

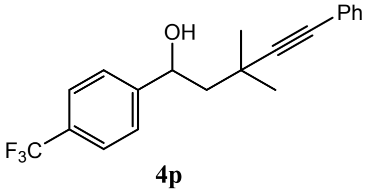

4p

4p was prepared according to general procedure **B** in 55% isolated yield. White solid. ^{1}H NMR (400 MHz, $CDCl_3$): δ 7.60 (d, J = 6.6 Hz, 2H), 7.51 (d, J = 6.6 Hz, 2H), 7.45-7.36 (m, 1H), 7.35-7.23 (m, 3H), 5.17 (d, J = 8.8 Hz, 1H), 3.30 (s, 1H), 2.08-1.96(m, 1H), 1.80-1.73 (m, 1H), 1.43 (dd, J = 6.4, 2.4 Hz, 3H). ^{13}C NMR (101 MHz, $CDCl_3$): δ 149.15, 131.69, 129.62 (q, J = 30.6 Hz), 128.47, 128.30, 126.16, 125.52 (q, J = 3.6 Hz), 122.99, 96.16, 82.98, 72.04, 52.81, 31.04, 30.76, 29.23. ^{19}F NMR (376 MHz, $CDCl_3$): δ -62.46 (s, 3F). IR (neat) cm^{-1} ṽ: 3297, 2970, 1926, 1619, 1444, 1420, 1321, 1160, 1131,1105, 1068, 1048, 1015, 842, 756, 692, 605. HRMS (ESI) m/z calcd. for $C_{20}H_{20}FO$ $[M+H]^+$: 333.1461; found: 333.1460.

3,3-dimethyl-5-phenyl-1-(4-(trifluoromethoxy)phenyl)pent-4-yn-1-ol

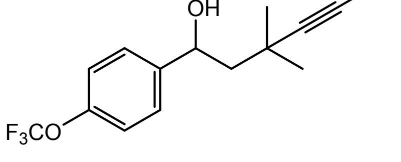

4q

4q was prepared according to general procedure **B** in 51% isolated yield. Colorless oil. ^{1}H NMR (400 MHz, $CDCl_3$): δ 7.47-7.38 (m, 4H), 7.35-7.28 (m, 3H), 7.24-7.16 (m,

2H), 5.12 (d, J = 9.2 Hz, 1H), 3.24 (br. s, 1H), 2.04 (dd, J = 13.6, 9.0 Hz, 1H), 1.75 (d, J = 14.3 Hz, 1H). 1.42 (s, 6H). ^{13}C NMR (101 MHz, $CDCl_3$): δ 148.47 (d, J = 1.7 Hz), 143.95, 131.69, 128.45, 128.24, 127.26, 123.08, 121.08, 120.64 (q, J = 258.1 Hz), 96.28, 82.88, 71.88 (d, J = 5.9 Hz), 52.83, 31.06, 30.77, 29.25. ^{19}F NMR (377 MHz, $CDCl_3$): δ -57.87 (s, 3F). IR (neat) cm^{-1} ṽ: 3436, 2969, 1598, 1508, 1255, 1219, 1169, 1016, 854, 755, 690, 544. HRMS (ESI) m/z calcd. for $C_{20}H_{20}F_3O$ [M+H]$^+$: 333.1461; found: 333.1460.

1-(2-bromo-6-fluorophenyl)-3,3-dimethyl-5-phenylpent-4-yn-1-ol

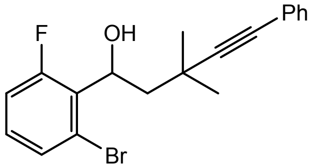

4r

4r was prepared according to general procedure **B** in 39% isolated yield. Colorless oil. ^{1}H NMR (400 MHz, $CDCl_3$): δ 7.48-7.39 (m, 4H), 7.34-7.28 (m, 3H), 6.90-6.79 (m, 1H), 5.39 (m, 1H), 3.63 (s, 1H), 1.84-1.80 (m, 2H), 1.53 (s, 3H), 1.41 (s, 3H). ^{13}C NMR (101 MHz, $CDCl_3$): δ 162.63 (d, J = 247.8 Hz), 146.19 (d, J = 8.0 Hz), 133.84 (d, J = 7.8 Hz), 131.72, 128.41 (d, J = 13.9 Hz), 122.89, 115.92 (d, J = 22.7 Hz), 115.07 (d, J = 24.2 Hz), 110.13, 96.34, 83.25, 71.20, 50.71, 31.44, 30.78, 28.70. ^{19}F NMR (376 MHz, $CDCl_3$): δ -113.98--114.04 (m, 1F). IR (neat) cm^{-1} ṽ: 3535, 2968, 2926, 2868, 1601, 1580, 1463, 1411, 1259, 1161, 1103, 1067, 1026, 882, 809, 755, 691, 631, 593, 563. HRMS (ESI) m/z calcd. for $C_{19}H_{19}BrFO$ [M+H]$^+$: 361.0598; found: 361.0597.

4-(9*H*-carbazol-9-yl)-4-methylpentan-2-ol

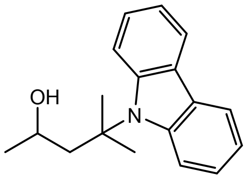

5a

5a was prepared according to general procedure **E** in 70% isolated yield. White solid. ^{1}H NMR (400 MHz, $CDCl_3$): δ 8.09 (dd, J = 7.7, 0.7 Hz, 2H), 7.85 (d, J = 8.6Hz, 2H), 7.37 (ddd, J = 8.6, 7.1, 1.4 Hz, 2H), 7.21 (t, J = 7.4 Hz, 2H), 3.80-3.70 (m, 1H), 2.51 (dd, J = 15.0, 2.9 Hz, 1H), 2.33 (dd, J = 15.0, 8.3 Hz, 1H), 2.19 (s, 3H), 2.01 (s, 3H), 1.01 (d, J = 6.2 Hz, 3H). ^{13}C NMR (101 MHz, $CDCl_3$): δ 140.87, 125.43, 124.61, 120.03, 118.92, 114.00, 65.73, 61.53, 49.24, 31.45, 30.81, 25.19. IR (neat) cm^{-1} ṽ: 3276, 2967, 2920, 2850, 1646, 1591, 1469, 1443, 1370, 1317, 1289, 1203, 1173, 1155, 1071, 1033, 747, 724, 627. HRMS (ESI) m/z calcd. for $C_{18}H_{21}NNaO$ [M+Na]$^+$: 290.1515; found: 290.1515.

9-(4-((diisopropyl(methyl)silyl)oxy)heptan-2-yl)-9*H*-carbazole

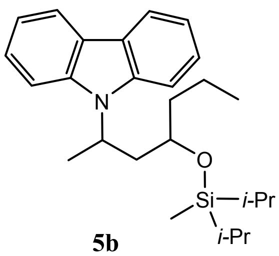

5b

5b was prepared according to general procedure **D** in 70% isolated yield. Colorless oil. ^{1}H NMR (400 MHz, $CDCl_3$): δ 8.11 (d, J = 7.7 Hz, 4H), 7.58-7.38 (m, 8H), 7.22 (t, J = 7.3 Hz, 4H), 5.05-4.93 (m, 2H), 3.67-3.56 (m, 2H), 2.60 -2.50 (m, 1H), 2.46-2.36 (m, 1H), 2.24-2.15 (m, 1H), 2.14-2.04 (m, 1H), 1.71-1.68 (d, J = 7.0 Hz, 3H), 1.68-1.63 (d, J = 7.0 Hz, 3H), 1.45-1.35 (m, 4H), 1.32-1.22 (m, 2H), 1.21-1.08 (m, 2H), 1.01-0.88 (m, 12H), 0.88-0.78 (m, 4H), 0.79- 0.70 (m, 6H), -0.07 (s, 3H), -0.13 (s, 3H). ^{13}C NMR (101 MHz, $CDCl_3$): δ 125.39, 120.41, 118.69, 118.66, 70.63, 69.81, 48.31, 47.55, 42.13, 41.76, 39.84, 39.24, 20.19, 19.57, 18.17, 18.14, 17.78, 17.76, 17.75, 17.69, 17.67, 17.63, 14.27, 13.81, 13.72, 13.67, 13.58, -6.93, -7.45. IR (neat) cm^{-1} ṽ: 2936, 2864, 1596, 1484, 1453, 1331, 1315, 1222, 1126, 1072, 1040, 995, 908, 882, 782, 746, 721, 641. HRMS (ESI) m/z calcd. for $C_{26}H_{40}NOSi$ $[M+H]^+$: 410.2874; found: 410.2873.

9-(3-((diisopropyl(methyl)silyl)oxy)-1-phenylpropyl)-9*H*-carbazole

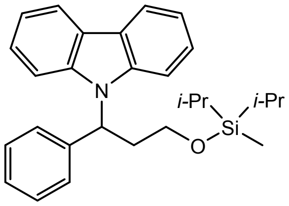

5c

5c was prepared according to general procedure **D** in 62% isolated yield. Colorless oil. ^{1}H NMR (400 MHz, $CDCl_3$): δ 8.12 (d, J = 7.8 Hz, 2H), 7.45-7.13 (m, 11H), 6.25 (dd, J = 9.6, 5.4 Hz, 1H), 3.62-3.54 (m, 1H), 3.25-3.20 (m, 1H), 2.83-2.72 (m, 2H), 0.97-0.75 (m, 14H), -0.16 (s, 3H). ^{13}C NMR (101 MHz, $CDCl_3$): δ 140.46, 140.37, 128.71, 127.37, 126.89, 125.51, 123.38, 120.27, 119.04, 110.77, 59.51, 53.24, 34.41, 17.53, 17.47, 12.99, 12.97, -8.65. IR (neat) cm^{-1} ṽ: 3059, 2940, 2843, 1596, 1483, 1451, 1331, 1251, 1121, 1098, 996, 954, 881, 789, 745, 721, 696, 644. HRMS (ESI) m/z calcd. for $C_{28}H_{36}NOSi$ $[M+H]^+$: 430.2561; found: 430.2559.

4-(2-chloro-9*H*-carbazol-9-yl)-4-methylpentan-2-ol

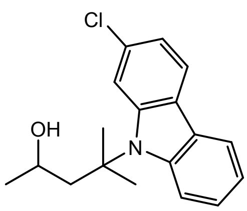

5d

5d was prepared according to general procedure **E** in 65% isolated yield. White solid. ^{1}H NMR (400 MHz, $CDCl_3$): δ 8.04 (d, J = 7.8 Hz, 1H), 7.97 (d, J = 8.3 Hz, 1H), 7.85 (s, 1H), 7.82 (d, J = 9.0 Hz, 1H), 7.37 (t, J = 7.9 Hz, 1H), 7.21 (t, J = 7.4 Hz, 1H), 7.18 (dd, J = 8.3, 0.9 Hz, 1H), 3.81- 3.64 (m, 1H), 2.48 (dd, J = 15.1, 2.8 Hz, 1H), 2.29 (dd, J = 15.1, 8.4 Hz, 1H), 2.18 (s, 3H), 1.99 (s, 3H), 1.02 (d, J = 6.2 Hz, 3H). ^{13}C NMR (101 MHz, $CDCl_3$): δ 141.30, 141.10, 131.01, 125.68, 124.04, 123.10, 120.61, 119.99, 119.44, 119.36, 114.27, 113.85, 65.64, 61.81, 49.01, 31.38, 30.70, 25.27. IR (neat) cm^{-1} ṽ: 3250, 2967, 2920, 1589, 1458, 1423, 1318, 1296, 1274, 1216, 1157, 1124, 1081, 999, 931, 808, 762, 744, 722. HRMS (ESI) m/z calcd. for $C_{18}H_{20}ClNNaO$ $[M+Na]^+$: 324.1126; found: 324.1124.

4-(2-methoxy-9*H*-carbazol-9-yl)-4-methylpentan-2-ol

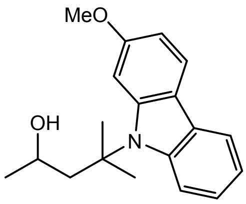

5e

5e was prepared according to general procedure **E** in 63% isolated yield. Colorless oil. ^{1}H NMR (400 MHz, $CDCl_3$): δ 7.98 (d, J = 7.7 Hz, 1H), 7.95 (d, J = 8.5 Hz, 1H), 7.79 (d, J = 8.5 Hz, 1H), 7.36 (d, J = 1.9 Hz, 1H), 7.29 (t, J = 7.8 Hz, 1H), 7.18 (t, J = 7.4 Hz, 1H), 6.85 (dd, J = 8.5, 1.9 Hz, 1H), 3.91 (s, 3H), 3.81-3.72 (m, 1H), 2.48 (dd, J = 15.0, 2.8 Hz, 2H), 2.30 (dd, J = 15.0, 8.3 Hz, 1H), 2.17 (s, 3H), 1.99 (s, 3H), 1.02 (d, J = 6.2 Hz, 4H). ^{13}C NMR (101 MHz, $CDCl_3$): δ 158.33, 142.02, 141.02, 124.76, 124.24, 120.36, 119.21, 119.06, 118.82, 113.86, 106.44, 99.65, 65.70, 61.44, 55.91, 49.07, 31.34, 30.64, 25.16. IR (neat) cm^{-1} ṽ: 3385, 2964, 2927, 1627, 1596, 1498, 1458, 1348, 1292, 1197, 1156, 1122, 1042, 819, 743, 724. HRMS (ESI) m/z calcd. for $C_{19}H_{23}NNaO_2$ $[M+Na]^+$: 320.1621; found: 320.1620.

4-(2,7-di-tert-butyl-9*H*-carbazol-9-yl)-4-methylpentan-2-ol

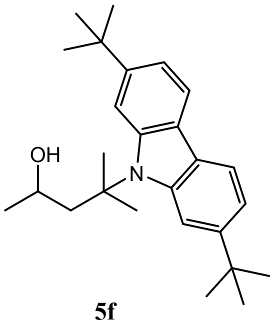

5f

5f was prepared according to general procedure **E** in 60% isolated yield. White solid. ^{1}H NMR (400 MHz, $CDCl_3$): δ 8.06 (d, J = 1.7 Hz, 2H), 7.73 (d, J = 8.9 Hz, 2H), 7.39 (dd, J = 8.9, 1.8 Hz, 2H), 3.85-3.76 (m, 1H), 2.45-2.31 (m, 2H), 2.11 (s, 3H), 1.99 (s, 3H), 1.44 (s, 18H), 1.04 (d, J = 6.2 Hz, 3H). ^{13}C NMR (101 MHz, $CDCl_3$): δ 141.53,

139.24, 124.51, 123.18, 115.85, 113.41, 65.75, 61.11, 49.31, 34.54, 32.02, 31.19, 30.77, 25.11. IR (neat) cm^{-1} ṽ: 3392, 2957, 2920, 2851, 1645, 1467, 1391, 1364, 1296, 1261, 1223, 1175, 1113, 1036, 878, 801, 653, 619. HRMS (ESI) m/z calcd. for $C_{26}H_{38}NO$ $[M+H]^+$: 380.2948; found: 380.2947.

4. Synthetic applications

4.1 Stereoselective Synthesis of tetrahydropyran derivative[3]

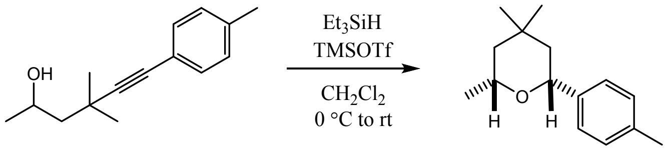

7
75%

Experimental procedure：To a magnetically stirred solution of alkynol **3a** (21.6 mg 0.1 mmol) in dry CH_2Cl_2 (2 mL) was added dropwise Et_3SiH (16 μL, 0.1 mmol) followed by TMSOTf (36 μL, 0.2 mmol) at 0 ℃. After completion, the mixture was then concentrated under reduced pressure. The residue was purified by column chromatography using ethyl acetate-petroleum ether (2:98) as eluent to give the product.

***cis*-2,4,4-trimethyl-6-(*p*-tolyl)tetrahydro-2*H*-pyran** 75% yield. Colorless oil. *cis*-single diastereomer (>19 : 1 dr). ^{1}H NMR (400 MHz, $CDCl_3$): δ 7.26 (d, *J* = 8.0 Hz, 2H), 7.14 (d, *J* = 8.0 Hz, 2H), 4.52 (dd, *J* = 11.6, 2.4 Hz, 1H), 3.81 (dqd, *J* = 12.2, 6.1, 2.2 Hz, 1H), 2.33 (s, 3H), 1.49 (dt, *J* = 13.4, 2.3 Hz, 1H), 1.43-1.34 (m, 2H), 1.24 (d, *J* = 6.1 Hz, 3H), 1.22-1.16 (m, 1H), 1.12 (s, 3H), 0.97 (s, 3H). ^{13}C NMR (101 MHz, $CDCl_3$): δ 140.69, 136.82, 129.05, 126.05, 75.62, 75.59, 69.90, 46.91, 46.51, 33.28, 30.14, 24.90, 22.39, 21.23. IR (neat) cm^{-1} ṽ: 2922, 2863, 1515, 1452, 1366, 1324, 1304, 1187, 1172, 1148, 1101, 1067, 1050, 1020, 992, 912, 810, 784, 584, 536. HRMS (ESI) m/z calcd. for $C_{15}H_{23}O$ $[M+H]^+$: 219.1743; found: 219.1744.

4.2 Stereoselective synthesis of pyrano-chromene derivative[4]

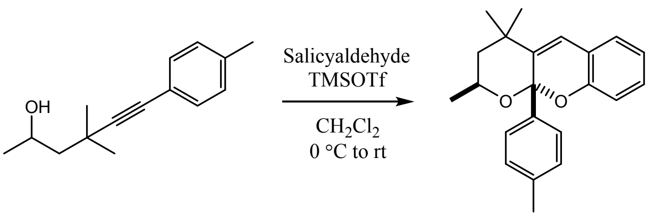

8
65%

Experimental procedure：To a magnetically stirred solution of alkynol **3a** (21.6 mg 0.1 mmol) , salicylaldehyde (11μL 0.1 mmol) in dry CH_2Cl_2 (2 mL) was added dropwise Et_3SiH (16 μL, 0.1 mmol) followed by TMSOTf (36 μL, 0.2 mmol) at 0 ℃. After completion, the mixture was then concentrated under reduced pressure. The residue was purified by column chromatography.

***trans*-2,4,4-trimethyl-10a-(*p*-tolyl)-3,4-dihydro-2*H*,10a*H*-pyrano[2,3-*b*]chromene**
White solid. *cis*-single diastereomer (>19 : 1 dr). ^{1}H NMR (400 MHz, $CDCl_3$): δ 7.42 (d, *J* = 8.2 Hz, 2H), 7.09 (dd, *J* = 7.4, 1.3 Hz, 1H), 7.03 (d, *J* = 8.1 Hz, 2H), 6.99 (dd, *J* = 7.7, 1.3 Hz, 1H), 6.83 (td, *J* = 7.4 Hz, 0.9 Hz, 1H), 6.70 (d, *J* = 7.9 Hz, 1H), 6.65 (s, 1H), 4.42-4.32 (m, 1H), 2.26 (s, 3H), 1.65-1.48(m, 2H), 1.37 (d, *J* = 6.2 Hz, 3H), 1.33 (s, 3H), 0.92 (s, 3H). ^{13}C NMR (101 MHz, $CDCl_3$): δ 152.13, 139.15, 138.16, 137.81, 128.95, 128.75, 126.26, 125.54, 122.91, 121.07, 121.03, 120.98, 116.51, 103.72, 67.68, 47.02, 33.88, 31.66, 29.34, 21.77, 21.26. IR (neat) cm^{-1} ṽ: 2956, 2922, 2866, 1646, 1484, 1456, 1366, 1275, 1232, 1170, 1136, 1275, 1232, 1170, 1136, 1108, 1069, 1045, 974, 925, 903, 857, 818, 796, 738, 721. HRMS (ESI) m/z calcd. for $C_{22}H_{25}O_2$ $[M+H]^+$: 219.1743; found: 219.1744.

5. Mechanistic investigations

5.1 Radical scavengers study

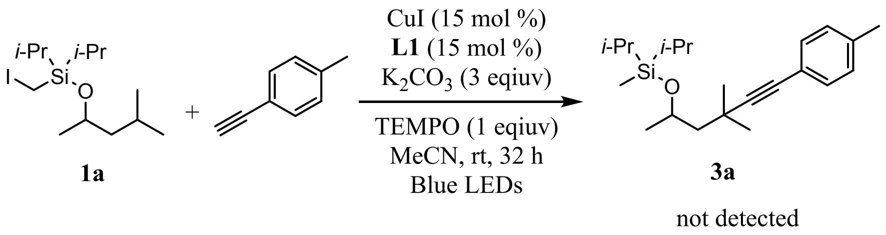

The radical scavenger experiments showed that TEMPO greatly hampered the reaction. This results evidently support radical-involved mechanism.

5.2 Observation of intermediate

The iodine atom transfer intermediate **1a** was observed by GC/MS during the reaction**.** The intermediate remained at a low concentration until it vanished at last. So the intermediate **1a** was most probably gradually converted to product.

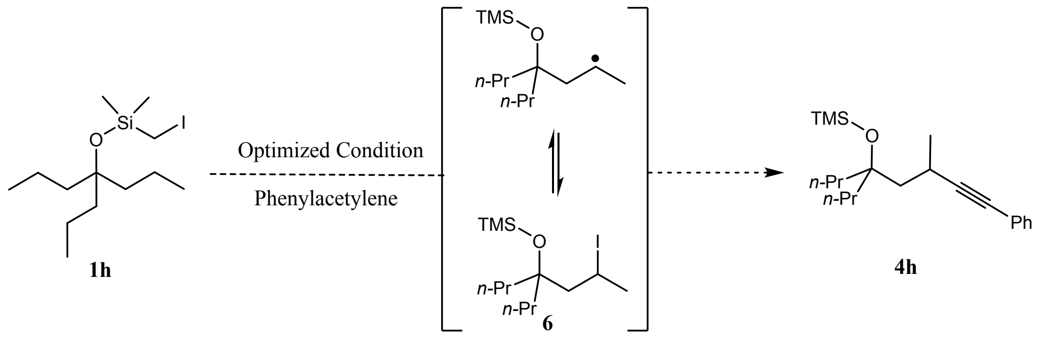

To verify the possibility of this process, we explored the reaction with phenylacetylene and intermediate **1a**. The intermediate **1a** was finally converted to wanted product with 70% isolated yield. So this process was proved to be reasonable.

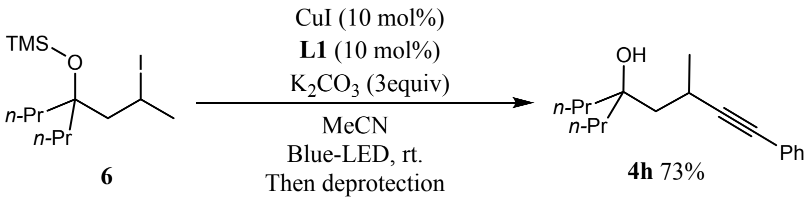

In a dried sealed vial, **L1** (0.015 mmol, 15 mol %), CuI (0.015 mmol, 15 mol %), K_2CO_3 (0.3 mmol, 3.0 equiv.) and phenylacetylene (0.2 mmol, 2 equiv.) were dissolved in anhydrous CH_3CN (2.0 mL) under a N_2 atmosphere, and the mixture was stirred for 30 min. Then iodide **6** (0.1 mmol, 1.0 equiv.) were added. The tube was sealed with Teflon septum, the reaction mixture was stirred under nitrogen at room temperature under Blue LED for 32 h. The vial distance from the lamp was about 2-3 cm. The resulting mixture was passed through a pad of silica gel, and concentrated under a reduced pressure. The residue oil was dissolved in THF (1.0 mL), and TBAF (2 mL, 1.0 M THF,) was added. After completion (monitored by TLC), the resulting mixture was poured into water with EtOAc. The aqueous layer was extracted with two portions of EtOAc. The combined extract was washed with brine, dried over Na_2SO_4 and concentrated. The residue oil was purified by column chromatography in hexanes/EtOAc to afford deprotected products with 73% isolated yield.

5.3 Photophysical studies

UV-visible Spectroscopic Study of substrates and complexes

Experimental details

The UV-Vis spectra of a single substrate was measured directly in MeCN. A mixture of two or more substrates was stirred for 30 min before its spectra was measured.[5]

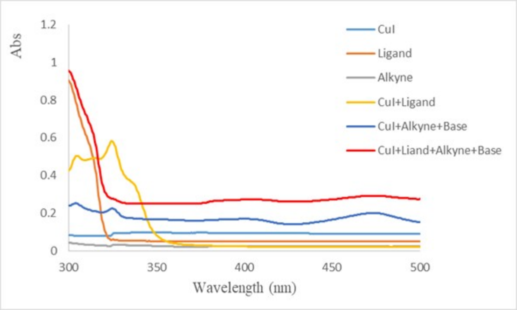

Figure S1. Absorption Spectra of reaction components: CuI (1×10^{-4} M), Ligand (1×10^{-4} M), Phenylacetylene (1×10^{-4} M), CuI (1×10^{-4} M) + Ligand (1×10^{-4} M), CuI (1×10^{-4} M) + Phenylacetylene (1×10^{-3} M) + Base, CuI (1×10^{-4} M) + Ligand (1×10^{-4} M) + Alkyne (1×10^{-3} M) + Base.

In the range of 380-500 nm, copper acetylide and copper acetylide-ligand showed obvious absorption.

Stern-Volmer Experiment

In order to figure out how the excited state of copper acetylide and copper acetylide-

ligand interacts with the tethered alcohols, luminescence quenching experiments were performed.[5]

Experimental procedure

In situ generated copper acetylide: phenylacetylene (0.1 mmol, 1.0 equiv), CuI (0.01 mmol, 10 mol %), K_2CO_3 (0.3 mmol, 3.0 equiv) were added in a dried reaction vessel with 2 mL MeCN and the reaction mixture was stirred at room temperature. After turned yellow, 100μl of the solution diluted with 2mL MeCN was added tethered alcohol **1a** (0.15-2.0 mM). The solution was transferred to the cuvette with 1 cm optical path utilizing an oven-dried glass Pasteur pipette. Fluorescence spectra of in situ generated copper acetylide (0.25 mM) is given in **Figure S2**.

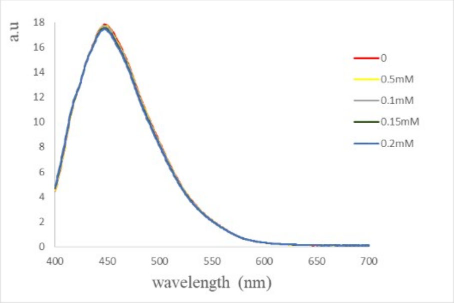

Figure S2. Fluorescence spectra of in situ generated copper acetylide

In situ generated copper acetylide-ligand phenylacetylene: CuI, **L1** and K_2CO_3 in MeCN: phenylacetylene (0.1 mmol, 1.0 equiv), CuI (0.01 mmol, 10 mol %), $\mathbf{L_1}$ (0.01 mmol, 10 mol %), K_2CO_3 (0.3 mmol, 3.0 equiv) were added in a dried reaction vessel with 2 mL MeCN and the reaction mixture was stirred at room temperature. After turned yellow, 100μl of the solution diluted with 2 mL MeCN was added tethered alcohol **1a** (0.15-2.0 mM). Sterm-Volmer plot for the emission quenching of in situ generated copper acetylide-ligand (0.25 mM) is given in Figure S3.

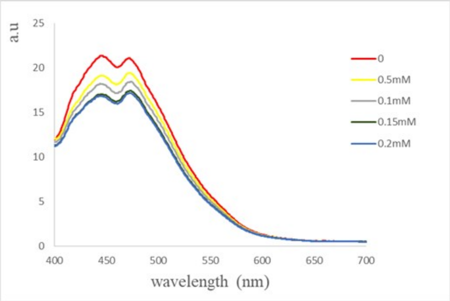

Figure S3. Fluorescence spectra of in situ generated copper acetylide-ligand

Stern-Volmer plot for in situ generated copper-ligand and copper acetylide-ligand quenched by tethered alcohol **1a** were showed in Figure S4. Based on Stern-Volmer constants, the excited-state copper acetylide cannot be quenched by the quencher. But the excited-state copper acetylide-ligand can be quenched by the tethered alcohol **1a**.

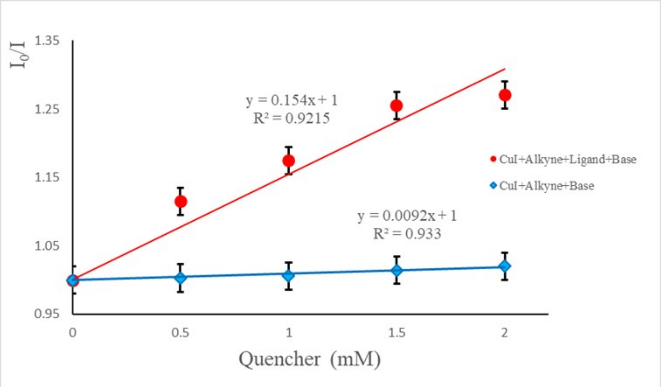

Figure S4. Stern-Volmer plots for 0.25 mM in situ generated copper-ligand and copper acetylide-ligand acetonitrile solution using 0-2.0 mM quencher.

5.4 Quantum yield measurement

We conducted the quantum yield measurement of the photoinduced reaction between tethered alcohols and terminal alkyne by an optical power meter. The result was calculated based on the product yield and the photon flux of the light source.

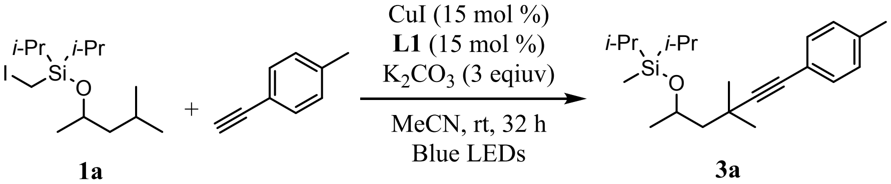

1a **3a**

4% ^{1}H NMR yield

Experimental procedure

In a dried sealed vial, **L1** (0.015 mmol, 15 mol %), CuI (0.015 mmol, 15 mol %), K_2CO_3 (0.3 mmol, 3.0 equiv.) and terminal alkyne (0.15 mmol, 1.5 equiv.) were dissolved in anhydrous CH_3CN (2.0 mL) under a N_2 atmosphere, and the mixture was stirred for 30 min. Then tethered alcohols (0.1 mmol, 1.0 equiv.) were added. The tube was sealed with Teflon septum, the reaction mixture was stirred under nitrogen at room temperature under Blue LED for 30min. The vial distance from the lamp was about 3-4 cm. The resulting mixture was passed through a pad of silica gel, and concentrated under a reduced pressure. Diethyl phthalate was internal standard for 1H NMR. The photon flux of the light source was determined by an optical power meter to be 82.2 mW.

$$\text{Photon flux} = \frac{P}{N_A \cdot hc/\lambda} = \frac{88.2 \times 10^{-3}}{6.02 \times 10^{23} \times 6.63 \times 10^{-34} \times 3 \times 10^{8}/400 \times 10^{-9}} = 2.95\times10^{-7}$$

(1)

Thus, the quantum yield can be calculated by the equation (2). f is the fraction of light (f>0.999), and assuming that the light was absorbed by the copper acetylide-ligand, the production of 4 × 10^{-6} mol product (4 % yield) in 30 min (1800 s) corresponds to a

quantum yield Φ = 0.75%.

$$\Phi = \frac{\text{mol product}}{\text{photon flux} \cdot t \cdot f} = \frac{4 \times 10^{-6}}{2.95 \times 10^{-7} \times 1800 \times 1} = 0.75\% \quad (2)$$

6. References

1 (a) P. Chuentragool, D. Yadagiri, T. Morita, S. Sarkar, M. Parasram, Y. Wang, V. Gevorgyan, *Angew. Chem. Int. Ed.* 2019, **58**, 1794-1798; (b) D. Kurandina, D. Yadagiri, M. Rivas, A. Kavun, P. Chuentragool, K. Hayama, V. Gevorgyan, *J. Am. Chem. Soc.* 2019, **141**, 8104-8109.

2 M. Parasram, V. O. Iaroshenko, V. Gevorgyan, *J. Am. Chem. Soc.* 2014, **136**, 17926-17929.

3 S. J. Gharpure, D. S. Vishwakarma, S. K. Nanda, *Org. Lett*. 2017, **19**, 6534-6537.

4 S. J. Gharpure, S. K. Nanda, Padmaja, Y. G. Shelke, *Chem. Eur. J.* 2017, **23**, 10007-10012.

5 Z. Li, J. Lei, *J. Am. Chem.Soc.* 2019*,* **141**, 9124-9128.

7. Spectral Data

^{1}H NMR (400 MHz, $CDCl_3$) of **1d**

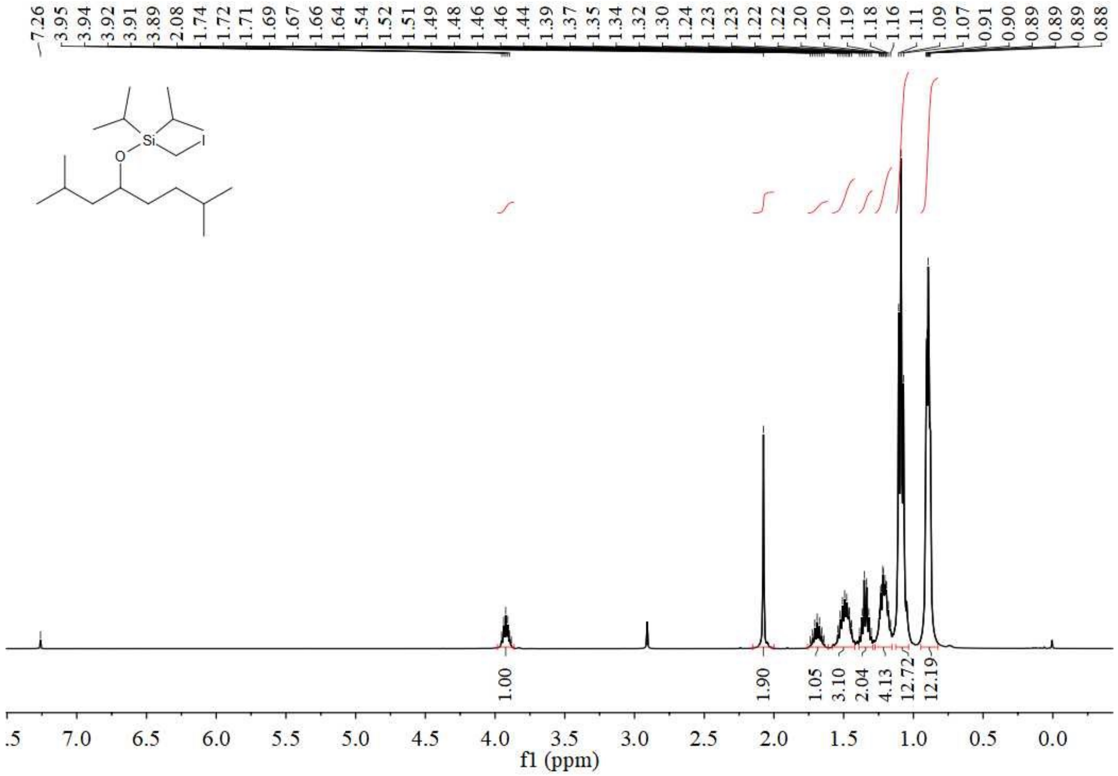

^{13}C NMR (101 MHz, $CDCl_3$) of **1d**

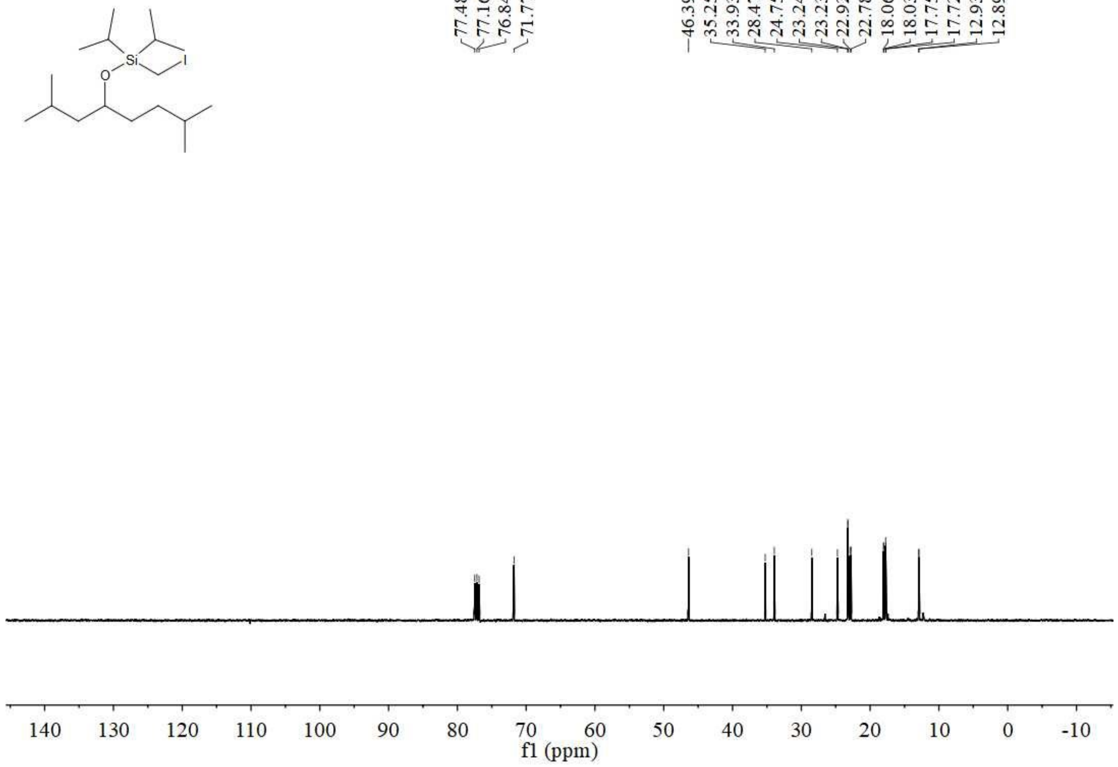

^{1}H NMR (400 MHz, $CDCl_3$) of **1i**

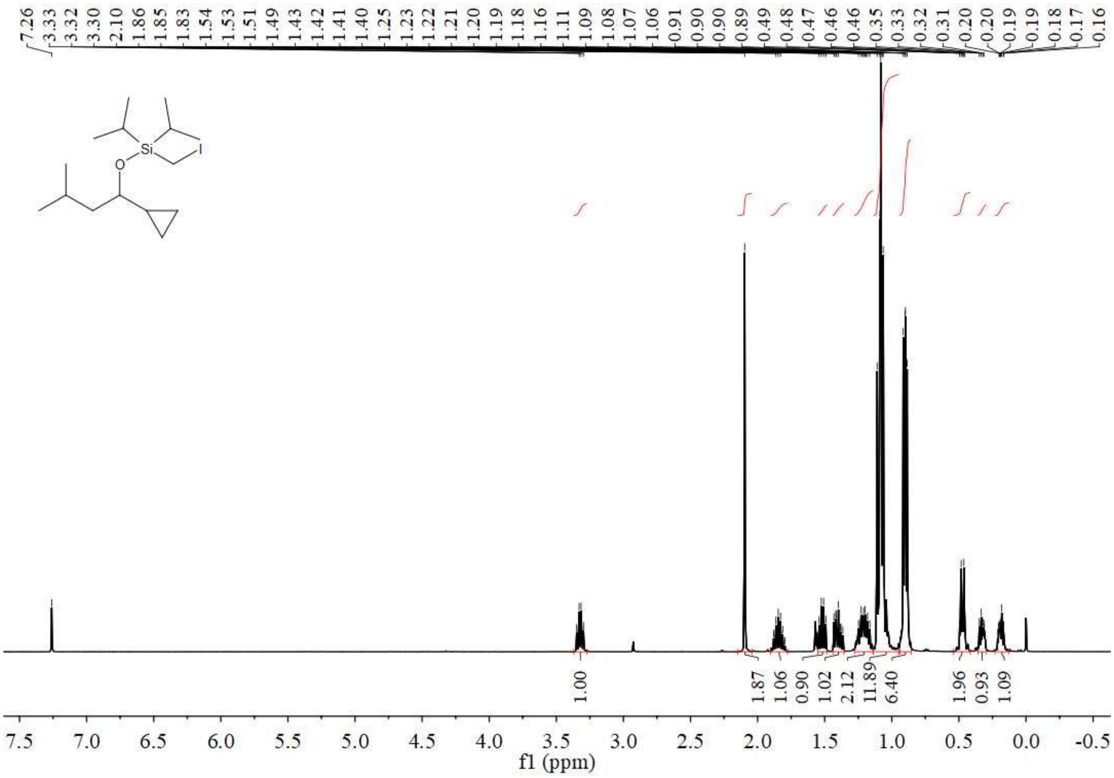

^{13}C NMR (101 MHz, $CDCl_3$) of **1i**

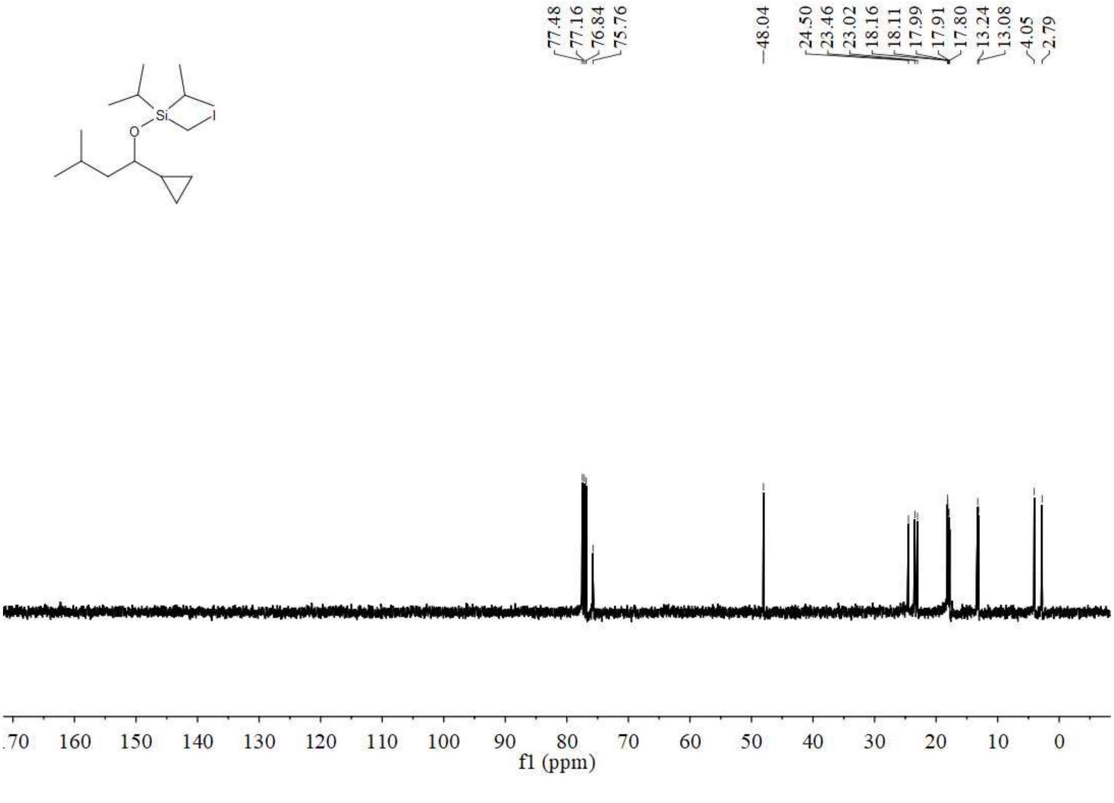

^{1}H NMR (400 MHz, $CDCl_3$) of **1j**

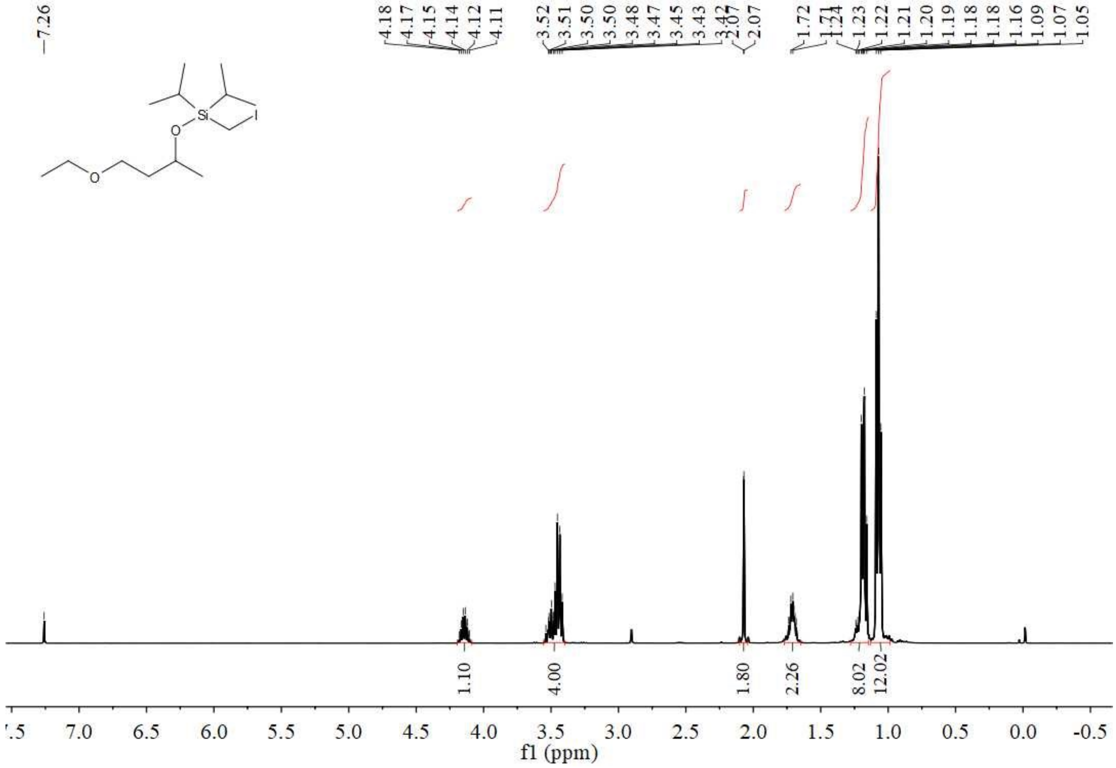

^{13}C NMR (101 MHz, $CDCl_3$) of **1j**

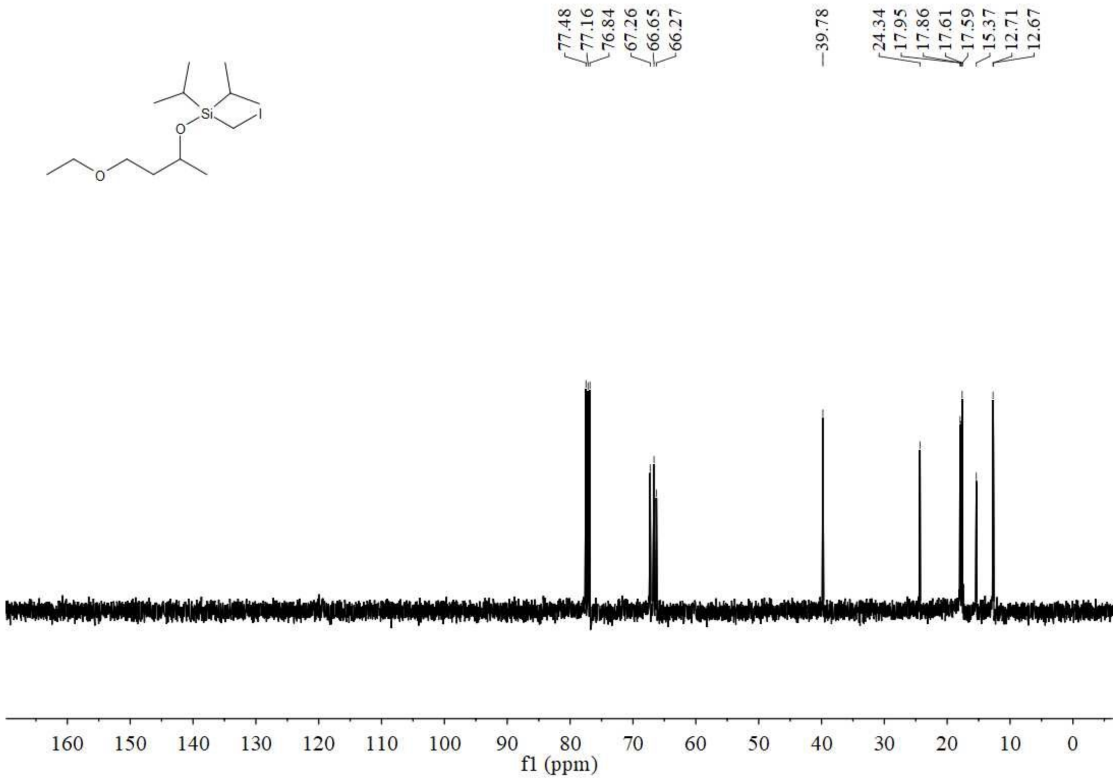

^{1}H NMR (400 MHz, $CDCl_3$) of **1k**

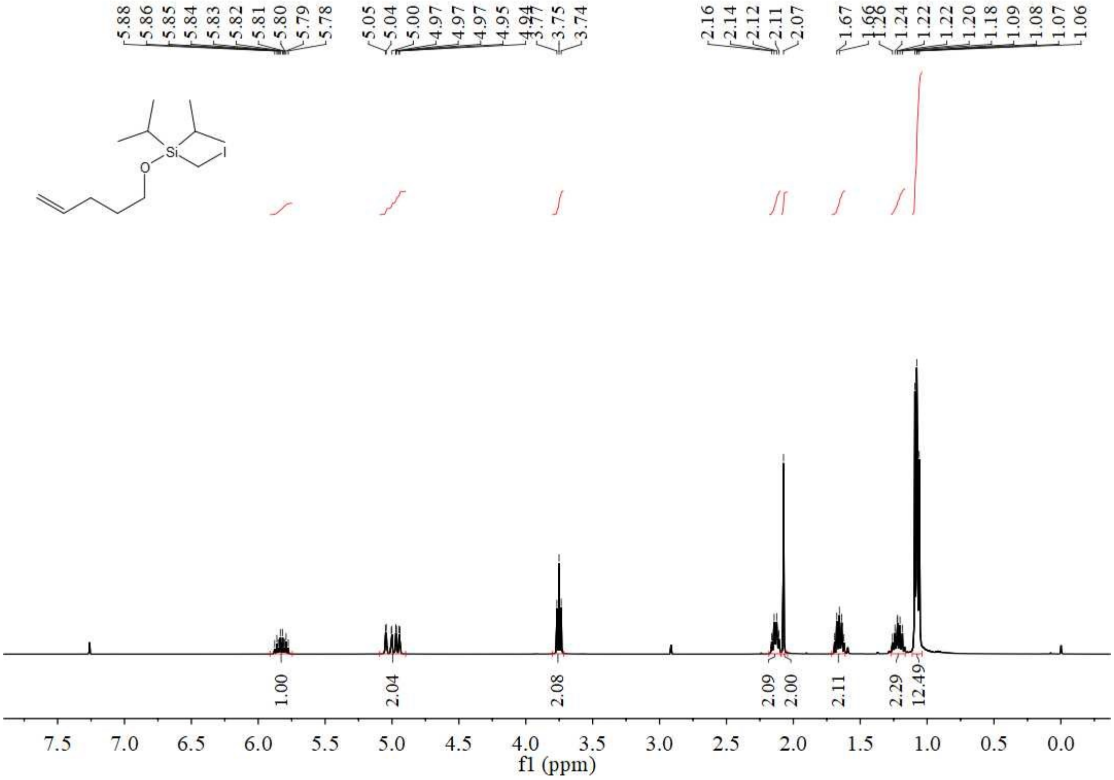

^{13}C NMR (101 MHz, $CDCl_3$) of **1k**

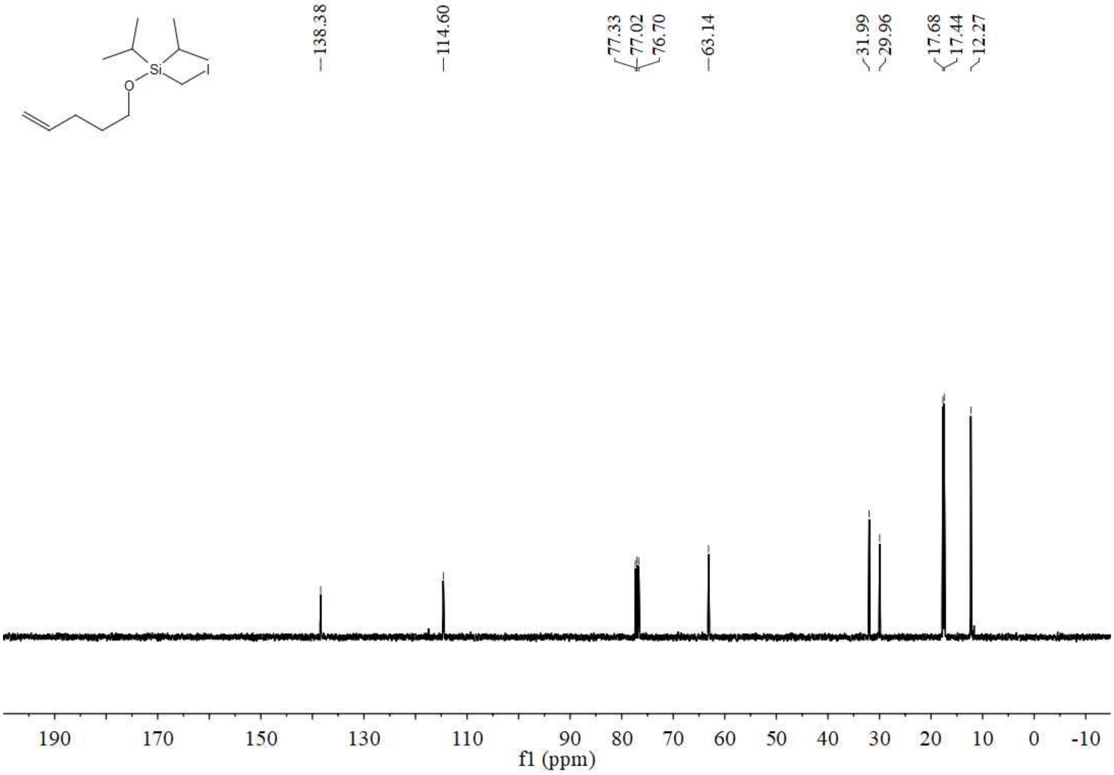

^{1}H NMR (400 MHz, $CDCl_3$) of **1l**

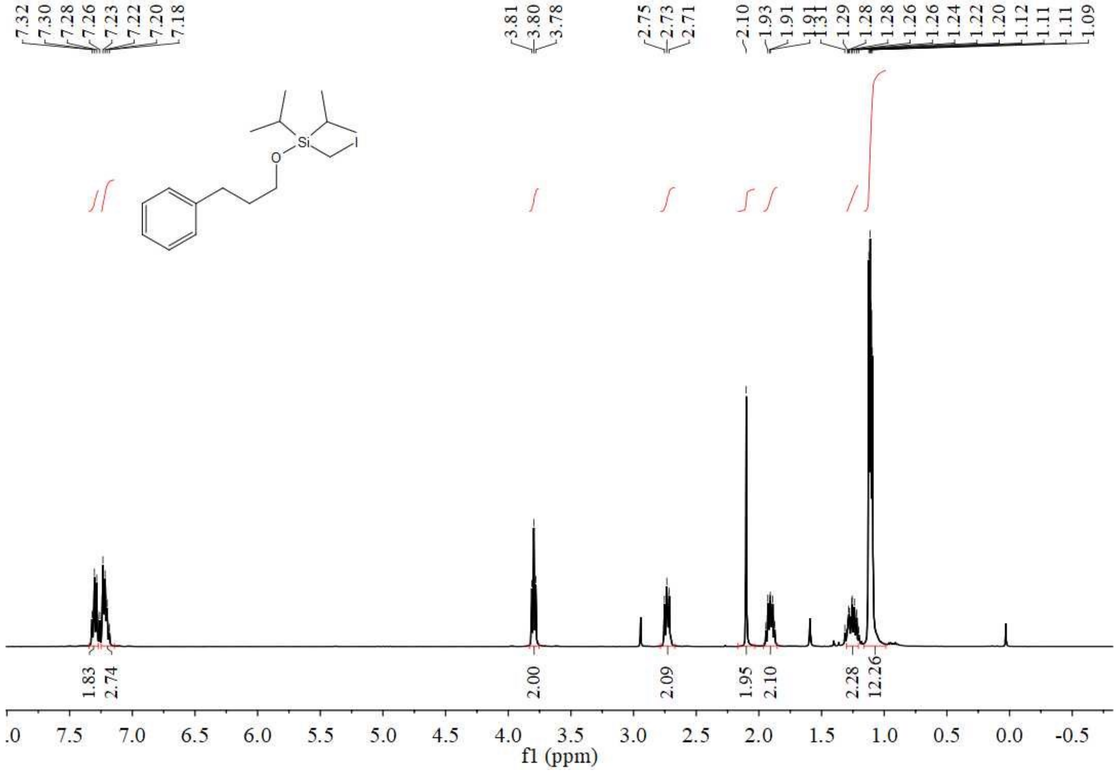

^{13}C NMR (101 MHz, $CDCl_3$) of **1l**

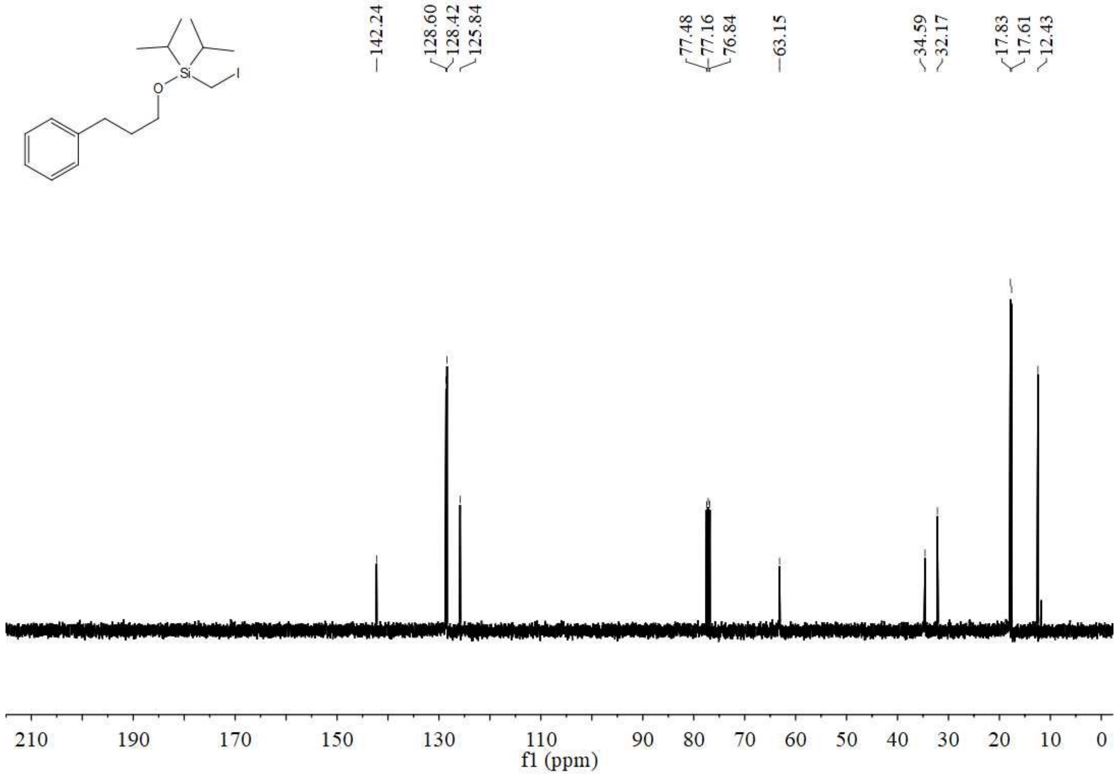

^{1}H NMR (400 MHz, $CDCl_3$) of **1m**

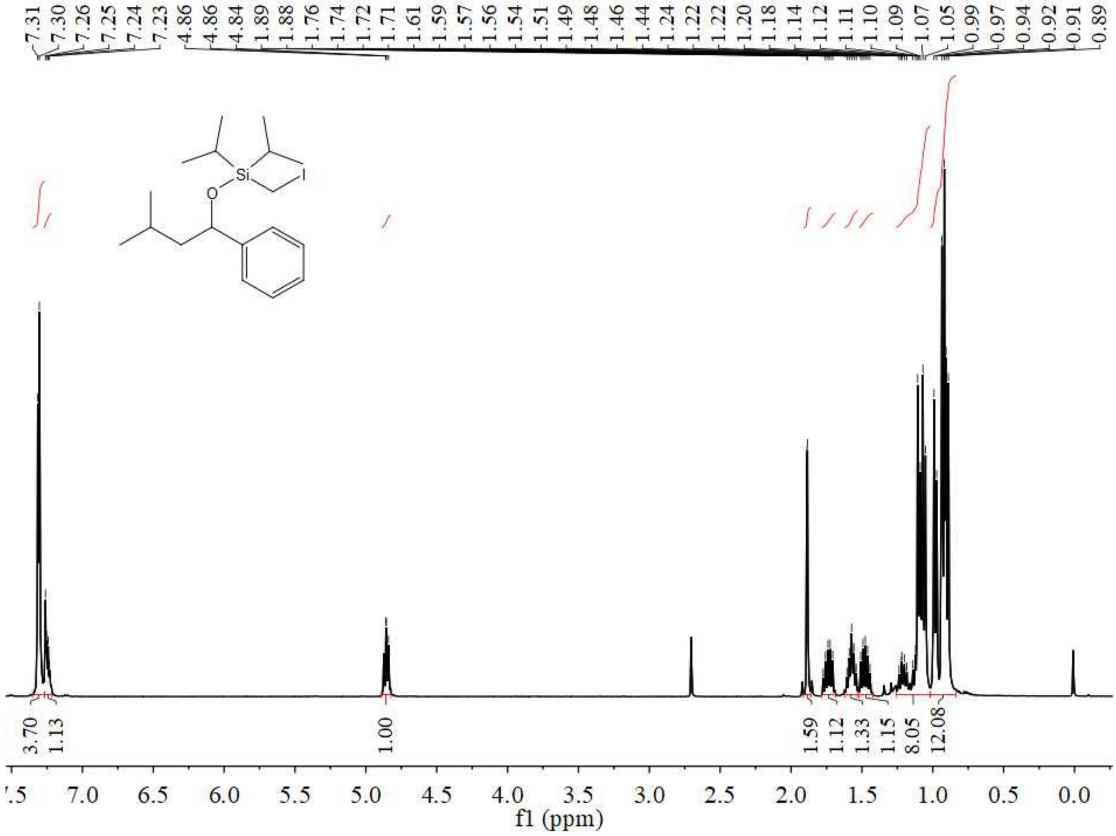

^{13}C NMR (101 MHz, $CDCl_3$) of **1m**

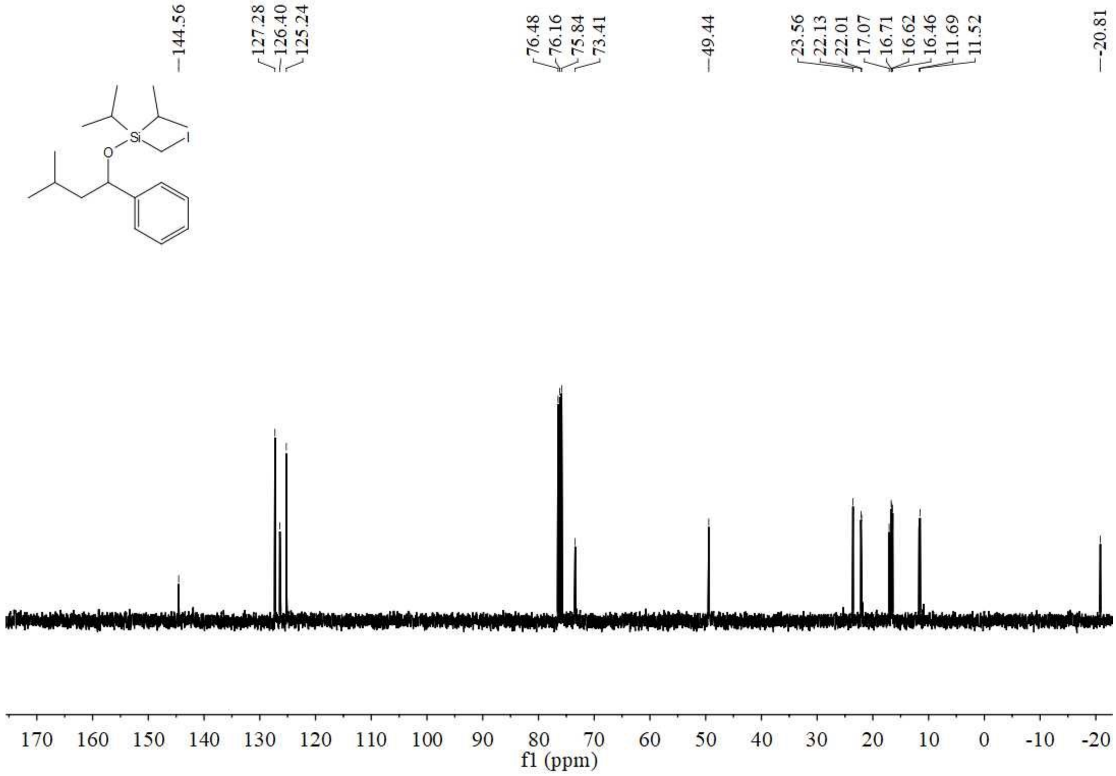

^{1}H NMR (400 MHz, $CDCl_3$) of **1n**

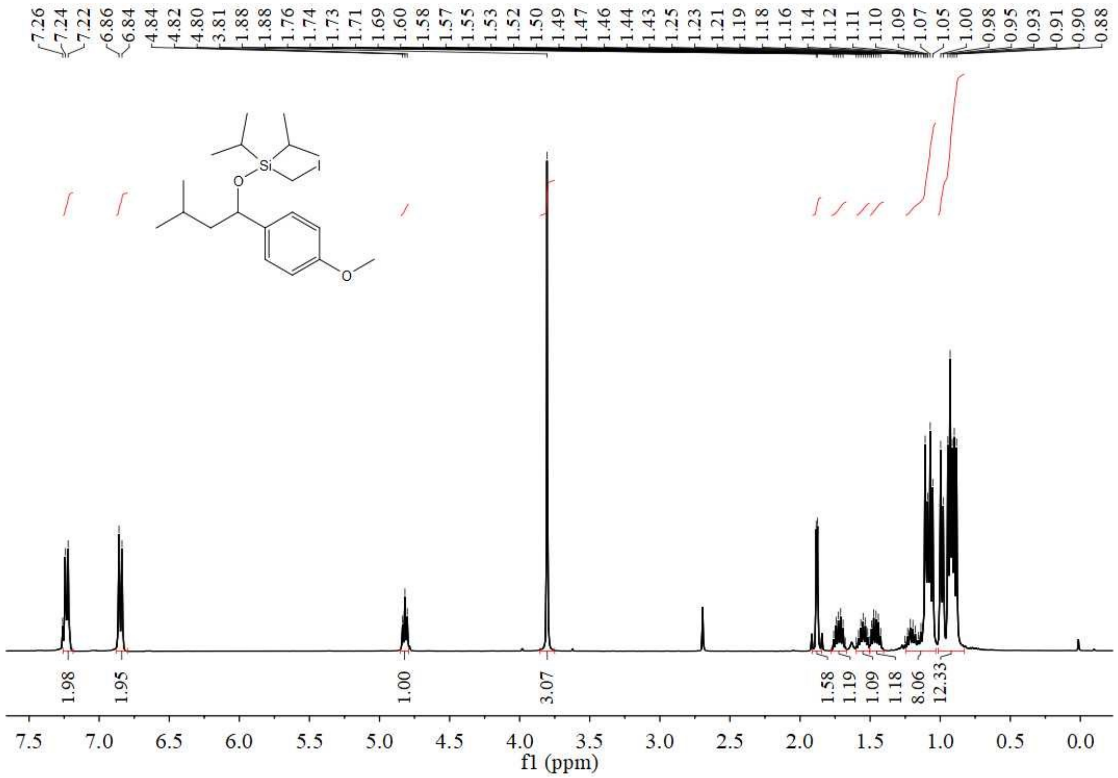

^{13}C NMR (101 MHz, $CDCl_3$) of **1n**

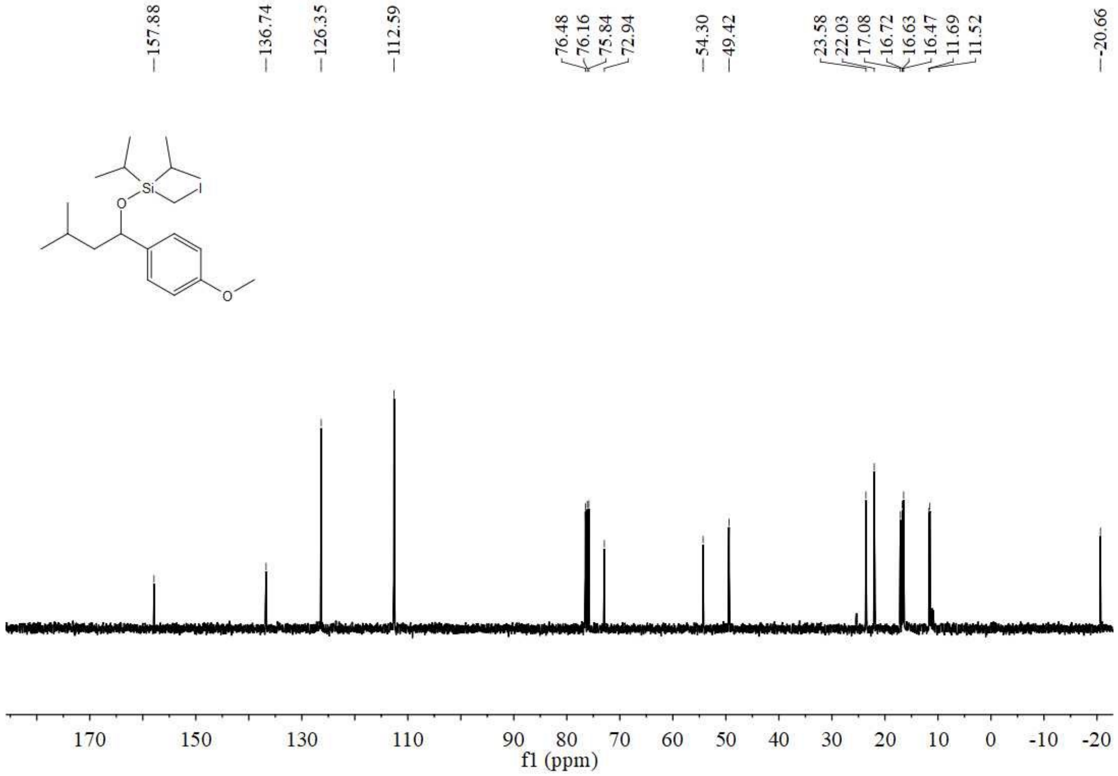

^{1}H NMR (400 MHz, $CDCl_3$) of **1o**

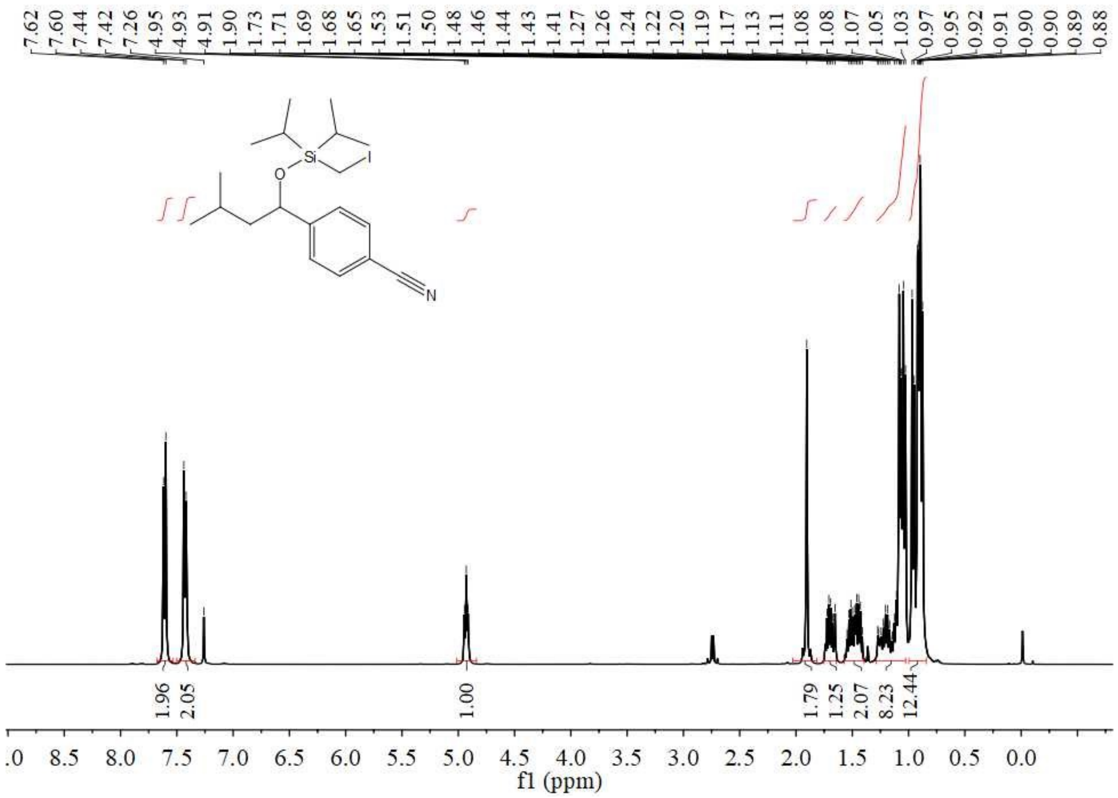

^{13}C NMR (101 MHz, $CDCl_3$) of **1o**

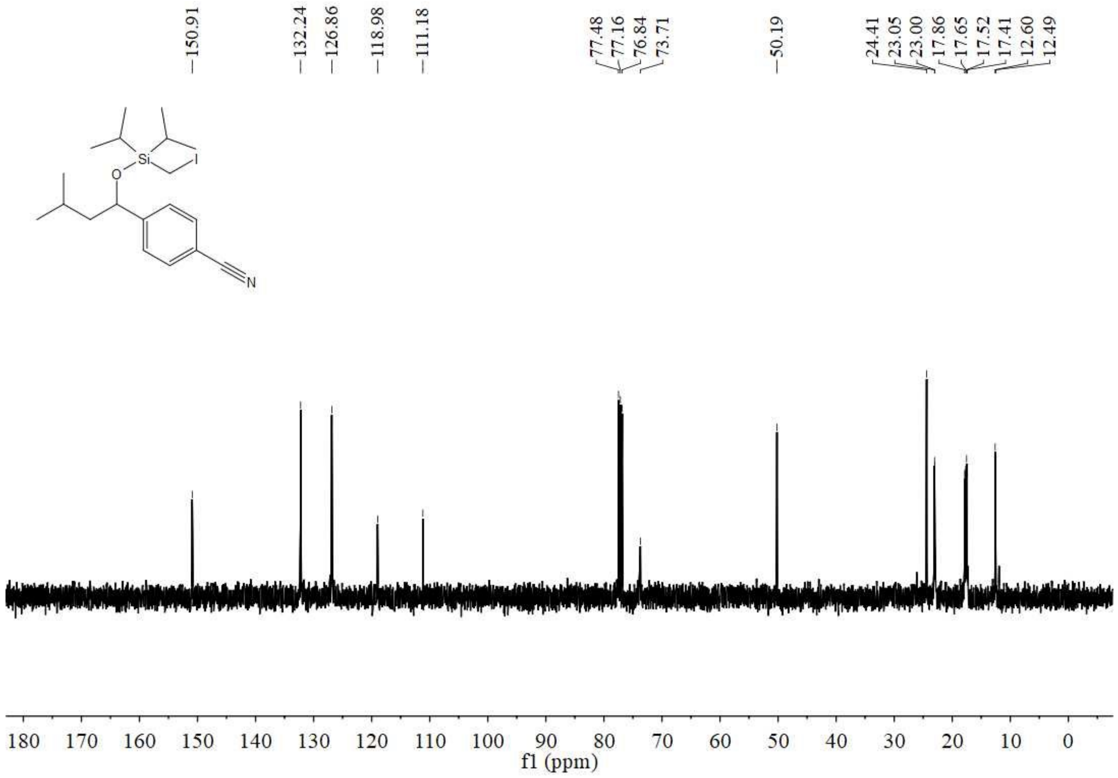

^{1}H NMR (400 MHz, $CDCl_3$) of **1p**

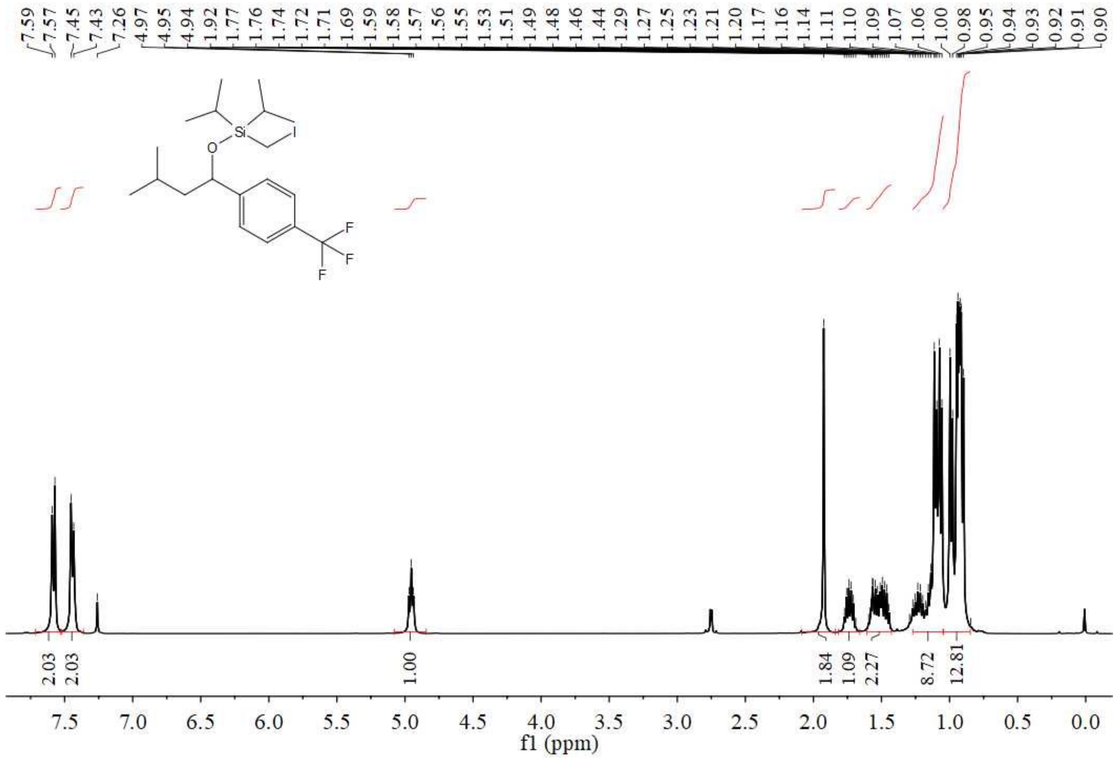

^{13}C NMR (101 MHz, $CDCl_3$) of **1p**

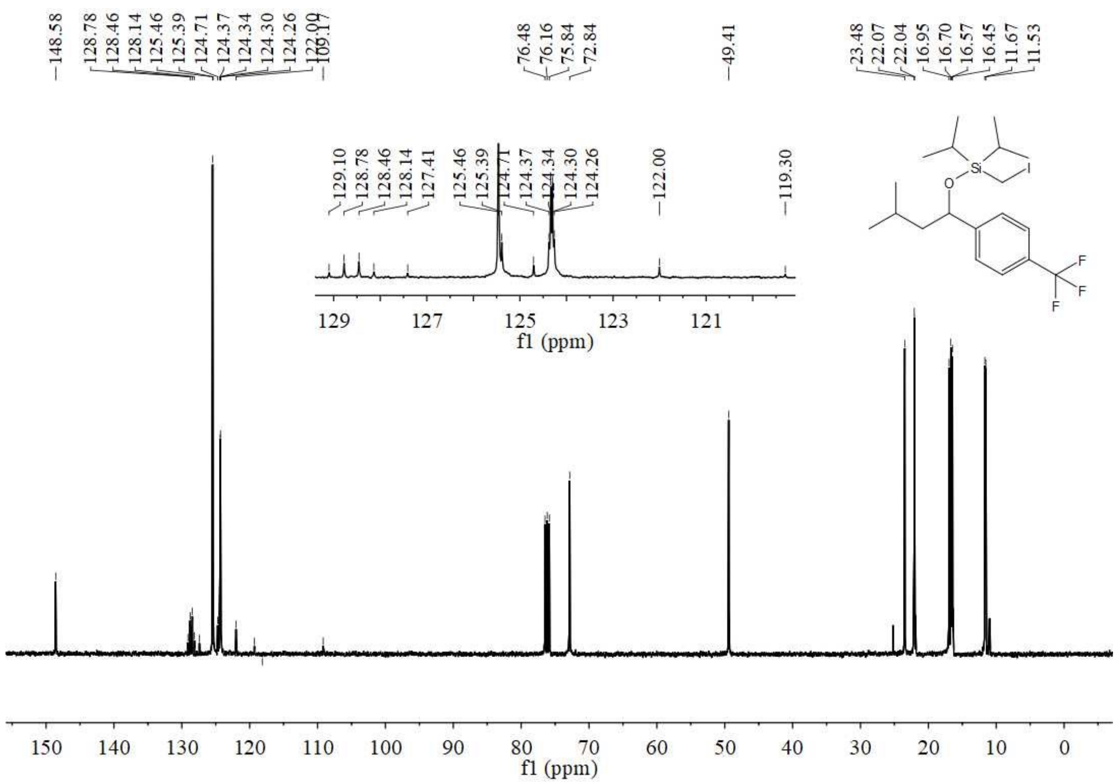

^{19}F NMR (376 MHz, CDCl$_3$) of **1p**

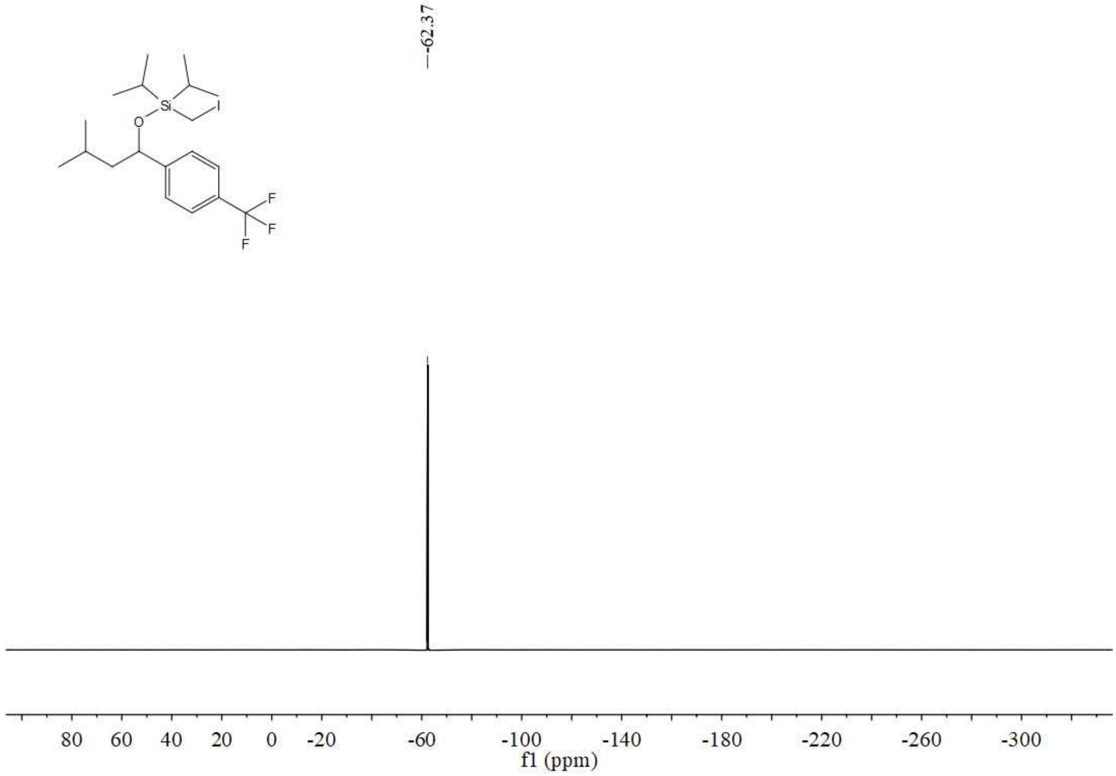

^{1}H NMR (400 MHz, CDCl$_3$) of **1q**

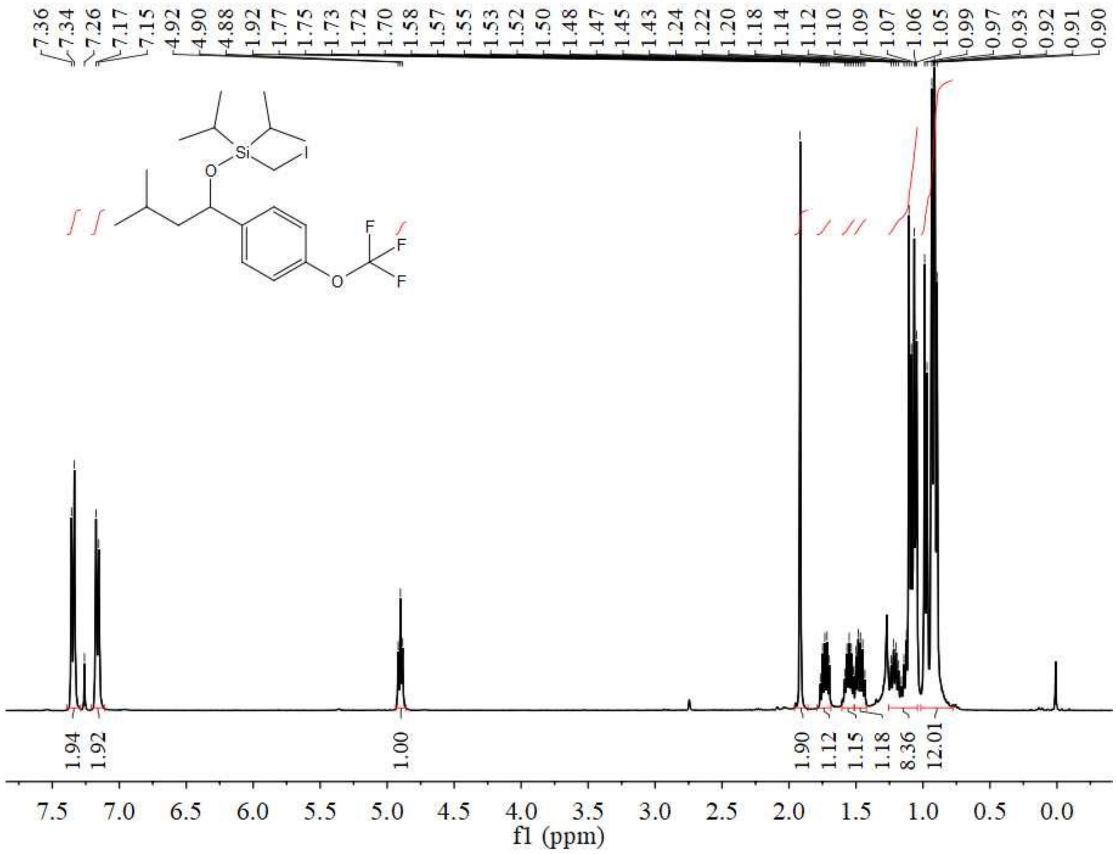

^{13}C NMR (400 MHz, $CDCl_3$) of **1q**

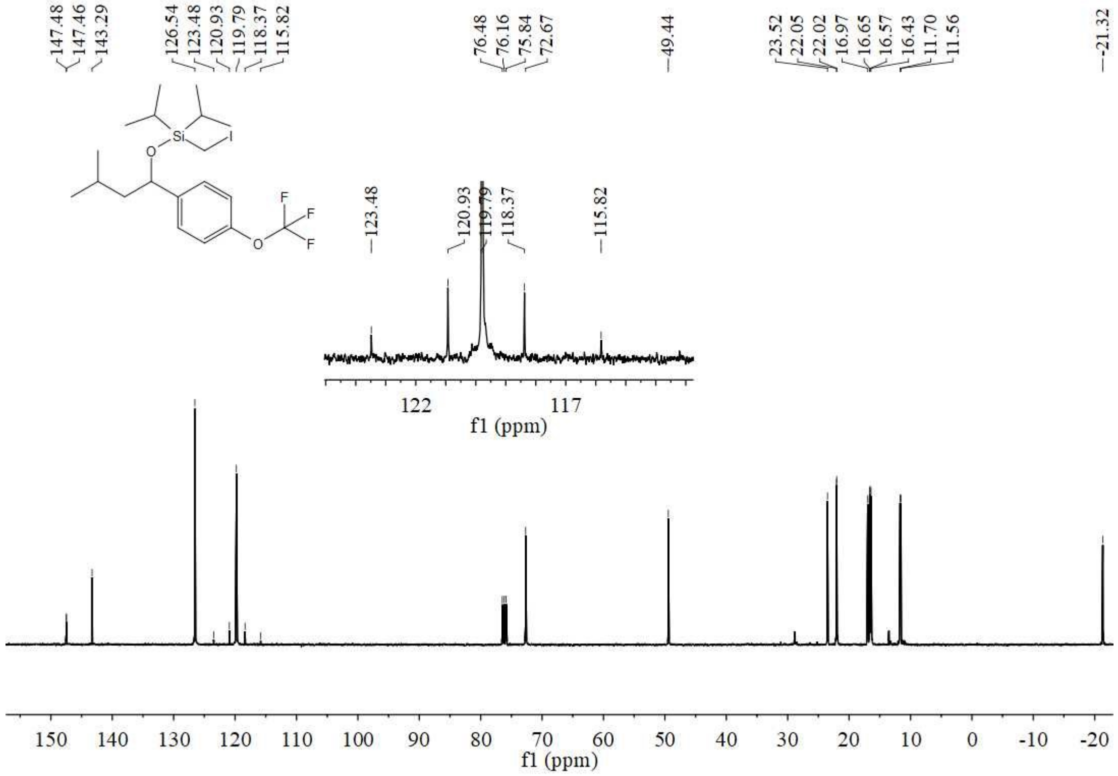

^{19}F NMR (376 MHz, $CDCl_3$) of **1q**

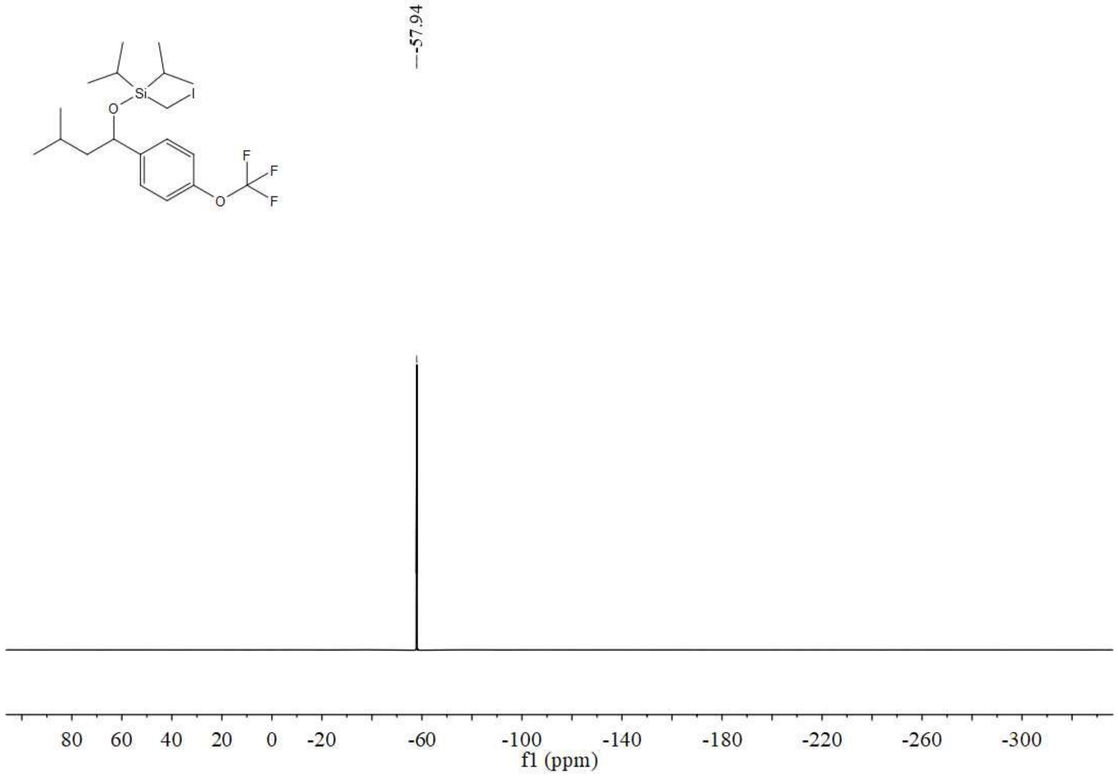

^{1}H NMR (400 MHz, $CDCl_3$) of **1r**

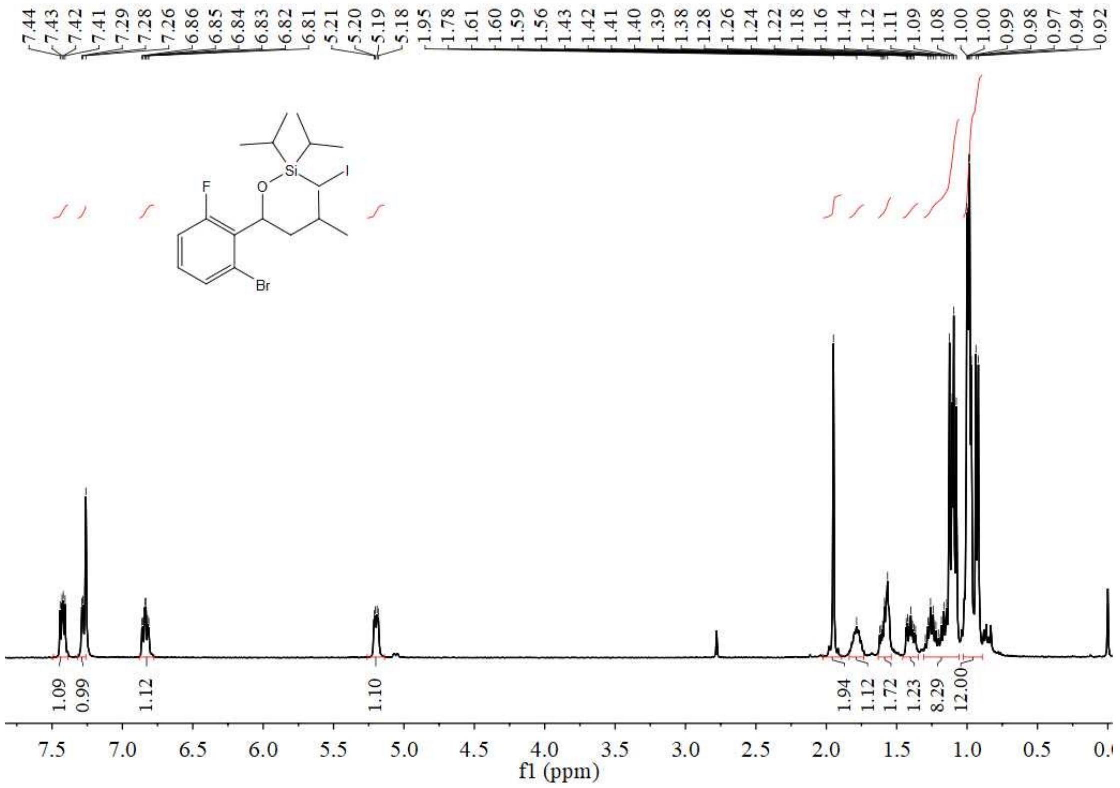

^{13}C NMR (101 MHz, $CDCl_3$) of **1r**

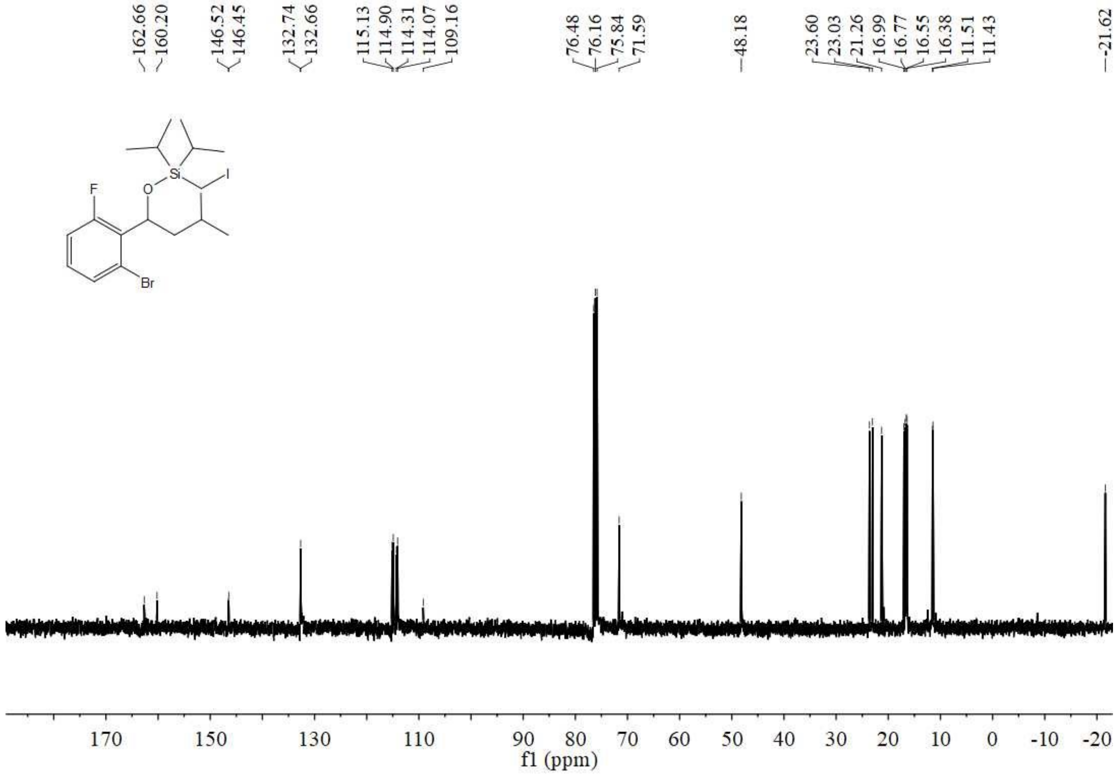

^{19}F NMR (376 MHz, $CDCl_3$) of **1r**

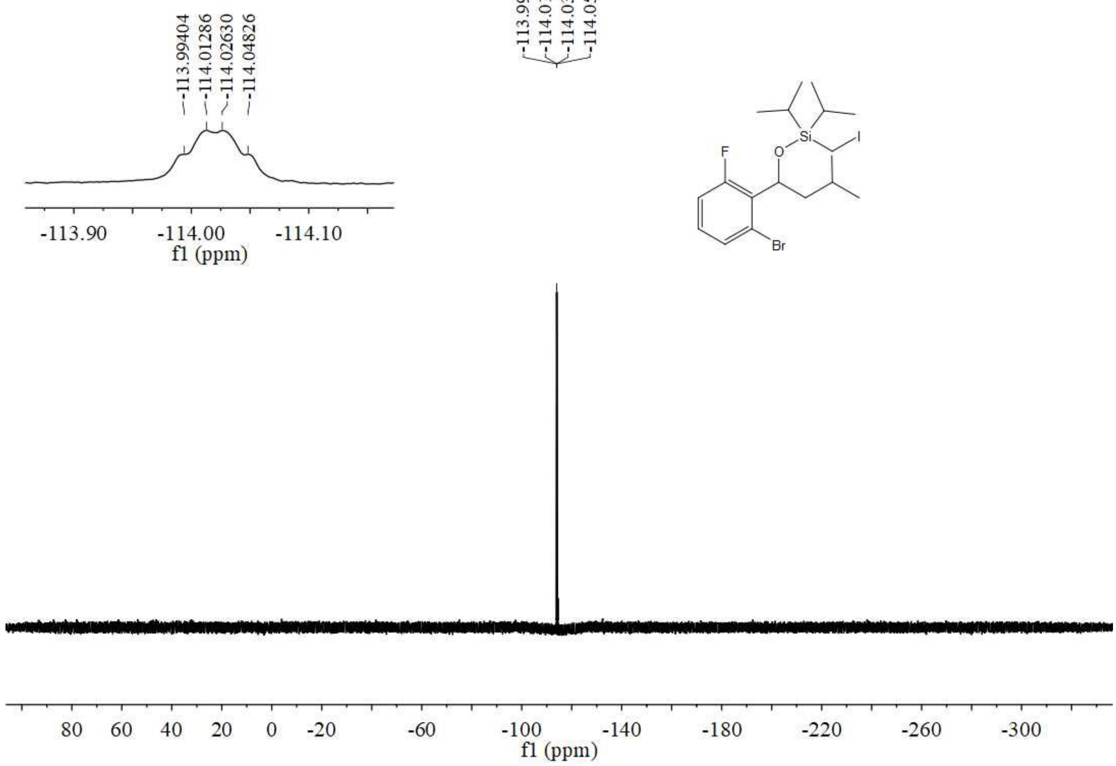

^{1}H NMR (400 MHz, $CDCl_3$) of **3a**

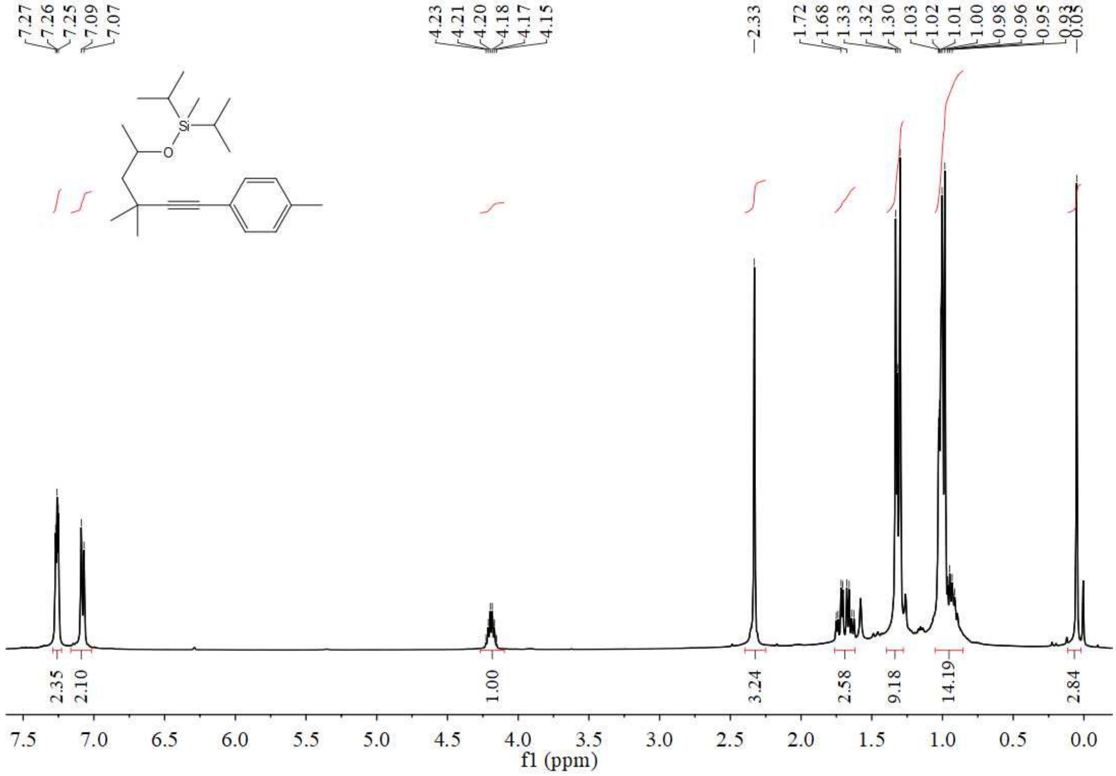

^{13}C NMR (101 MHz, CDCl$_3$) of **3a**

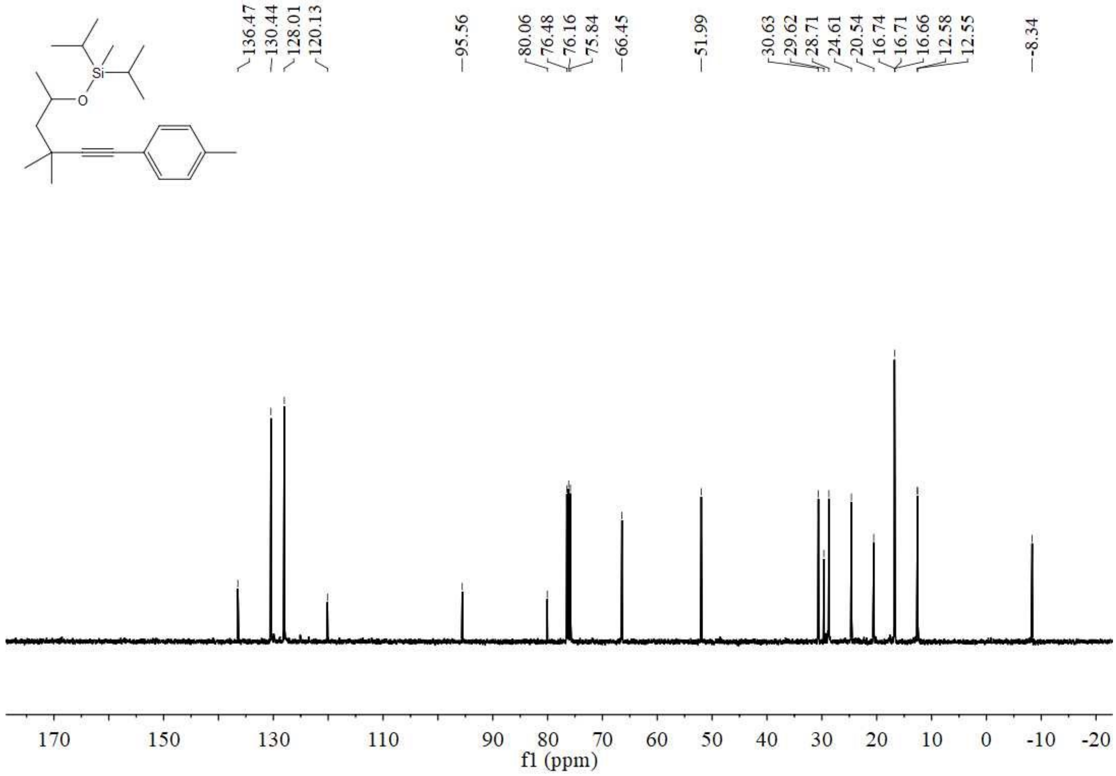

^{1}H NMR (400 MHz, CDCl$_3$) of **3b**

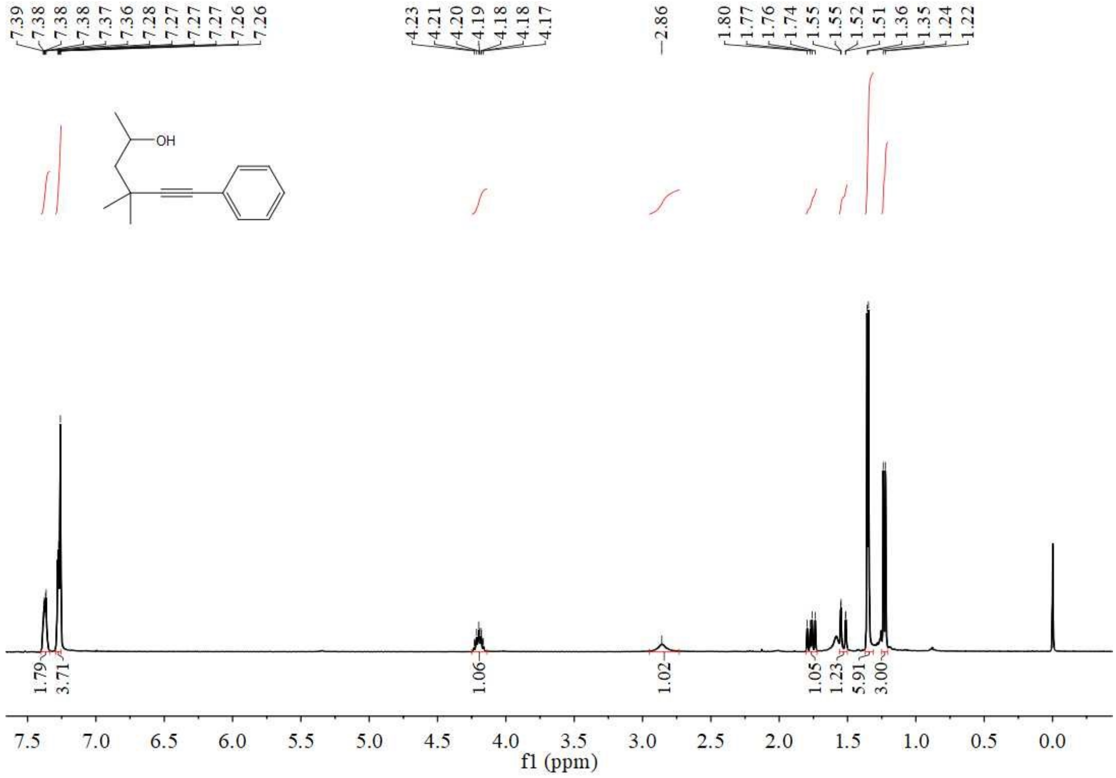

^{13}C NMR (101 MHz, $CDCl_3$) of **3a**

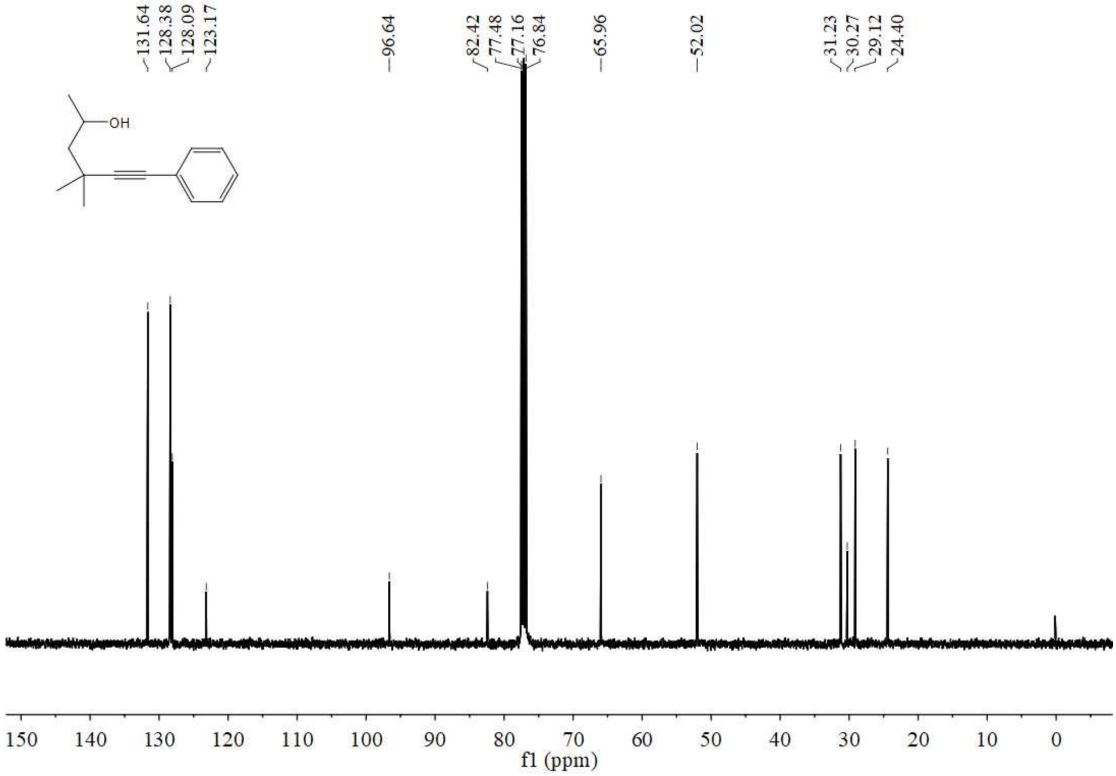

^{1}H NMR (400 MHz, $CDCl_3$) of **3c**

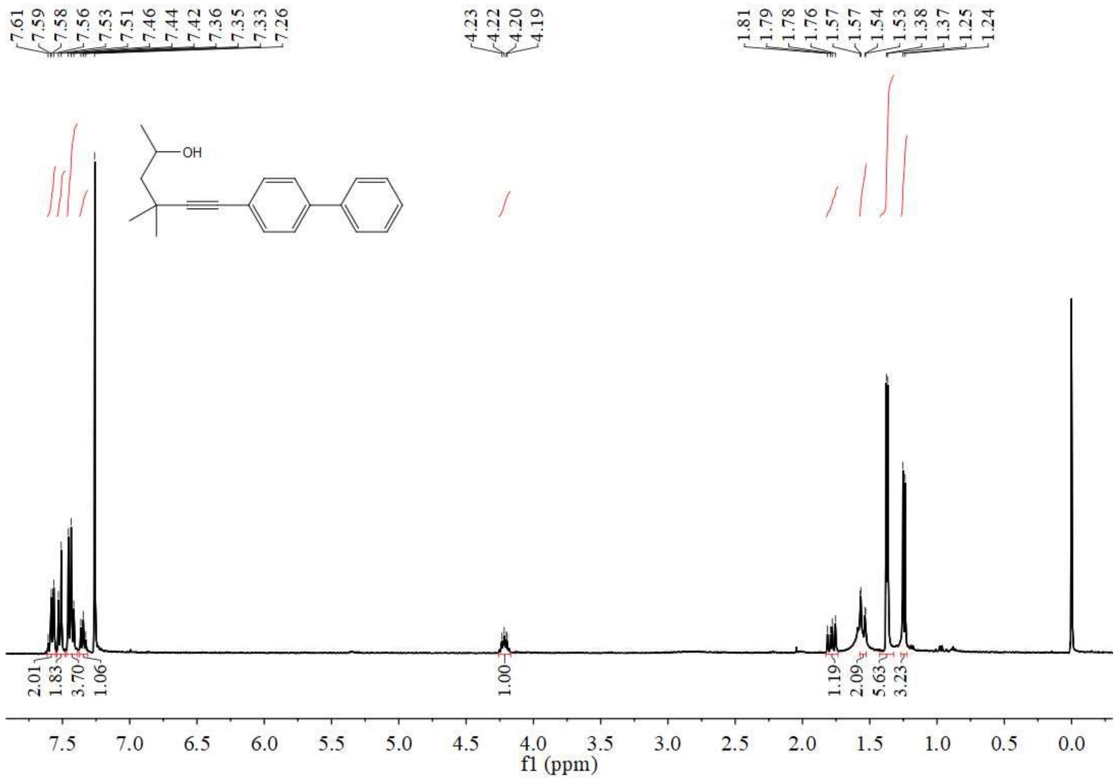

^{13}C NMR (101 MHz, $CDCl_3$) of **3c**

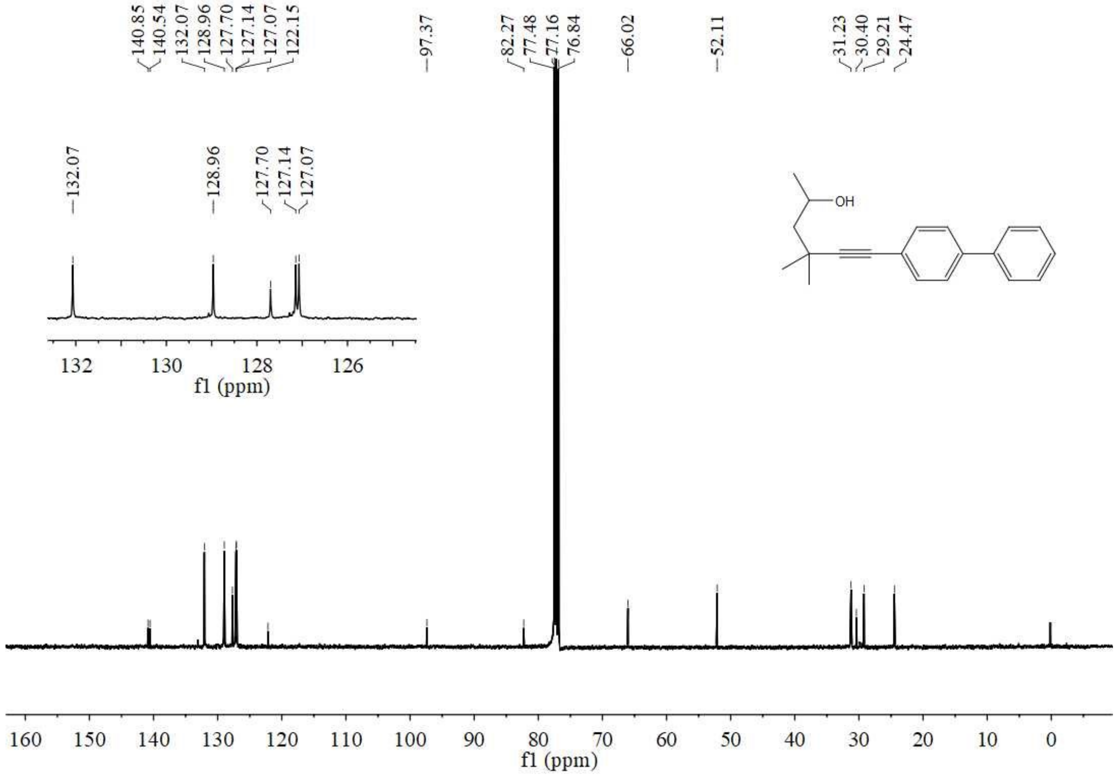

^{1}H NMR (400 MHz, $CDCl_3$) of **3d**

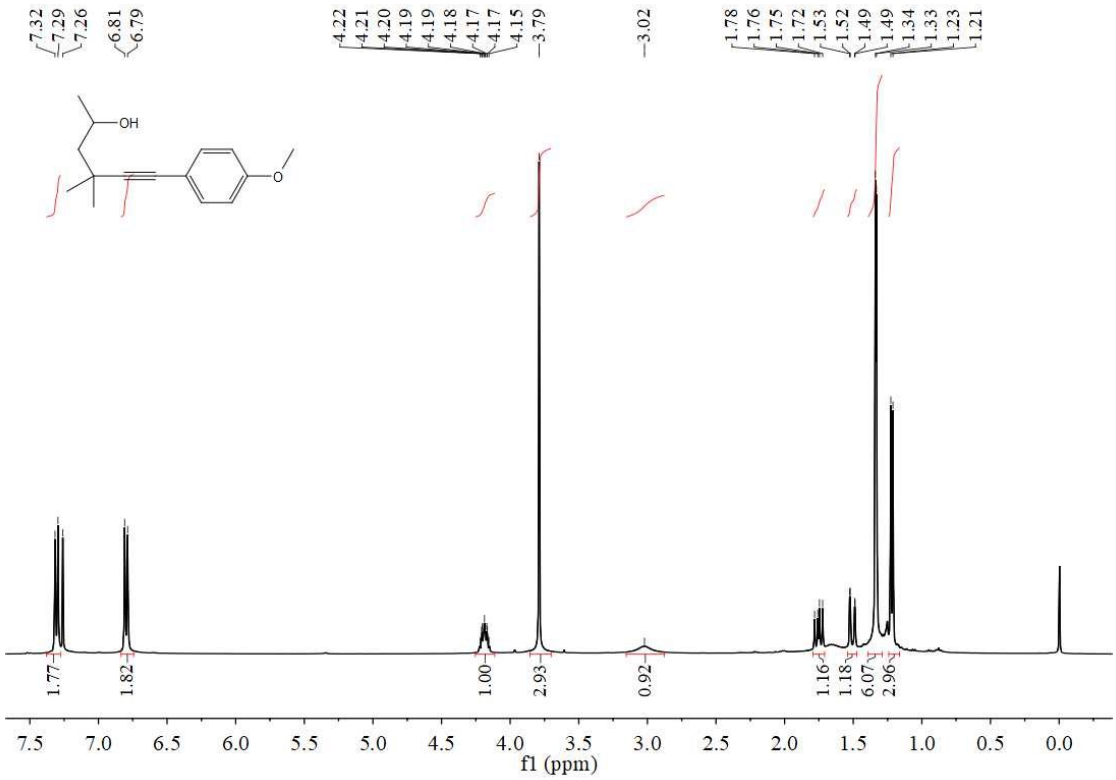

^{13}C NMR (101 MHz, $CDCl_3$) of **3d**

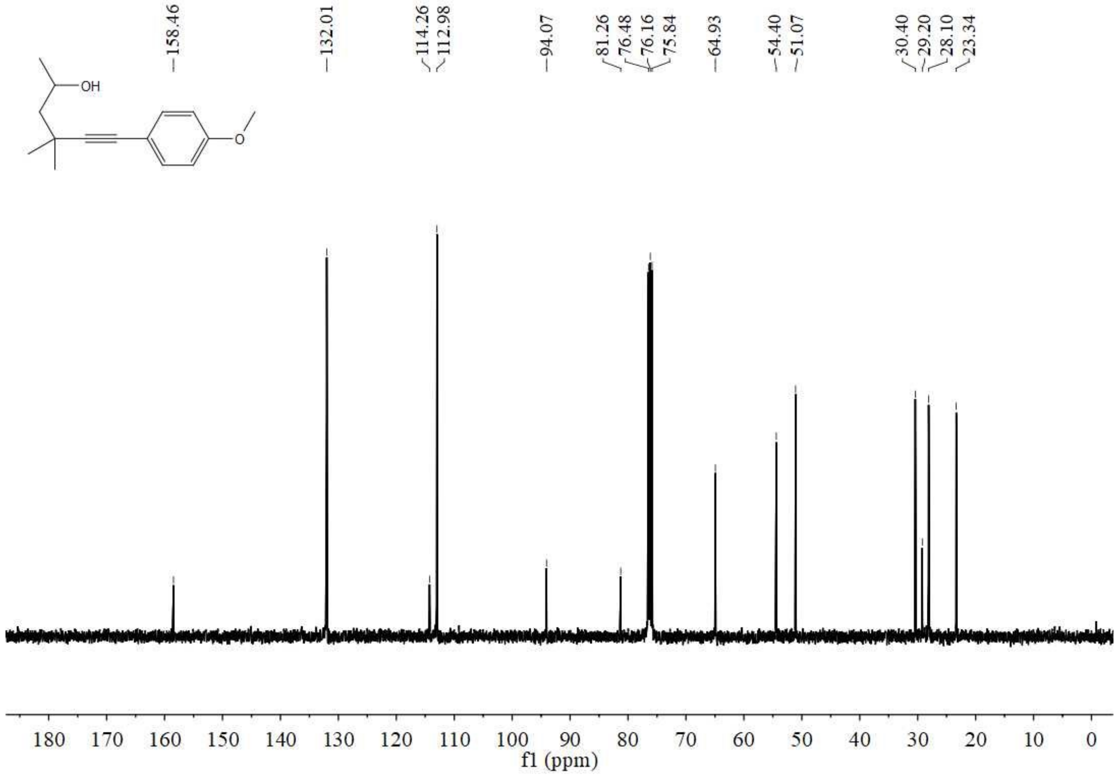

^{1}H NMR (400 MHz, $CDCl_3$) of **3e**

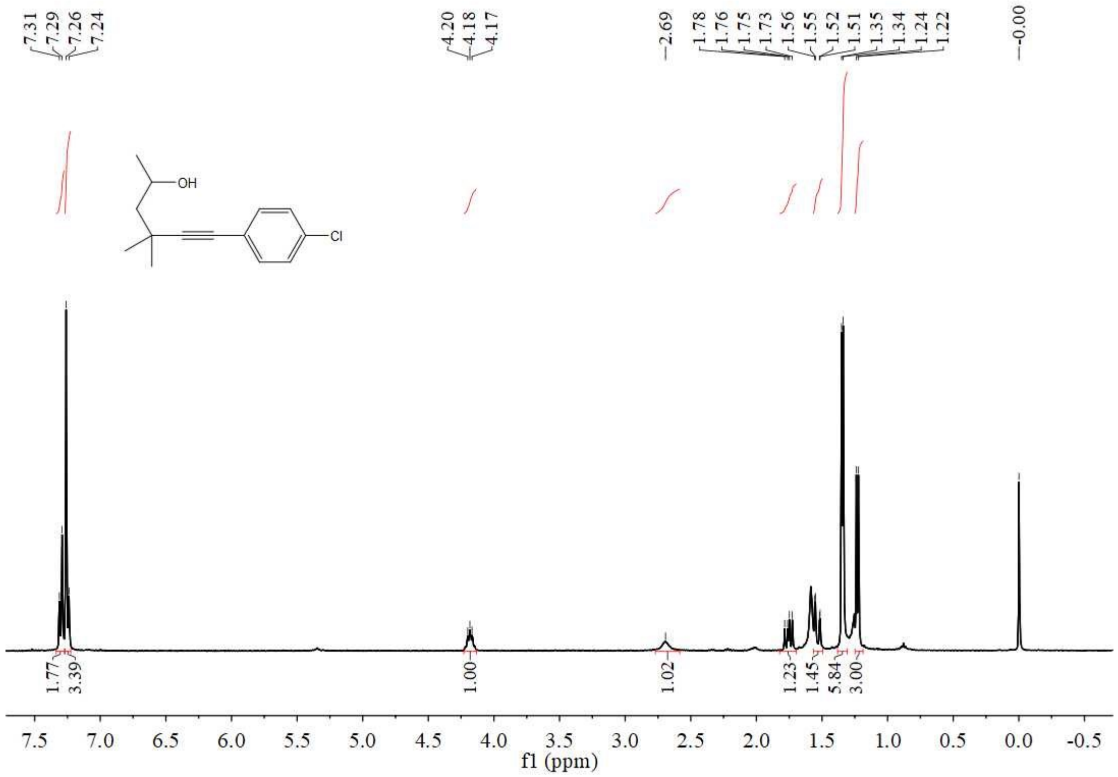

^{13}C NMR (101 MHz, $CDCl_3$) of **3e**

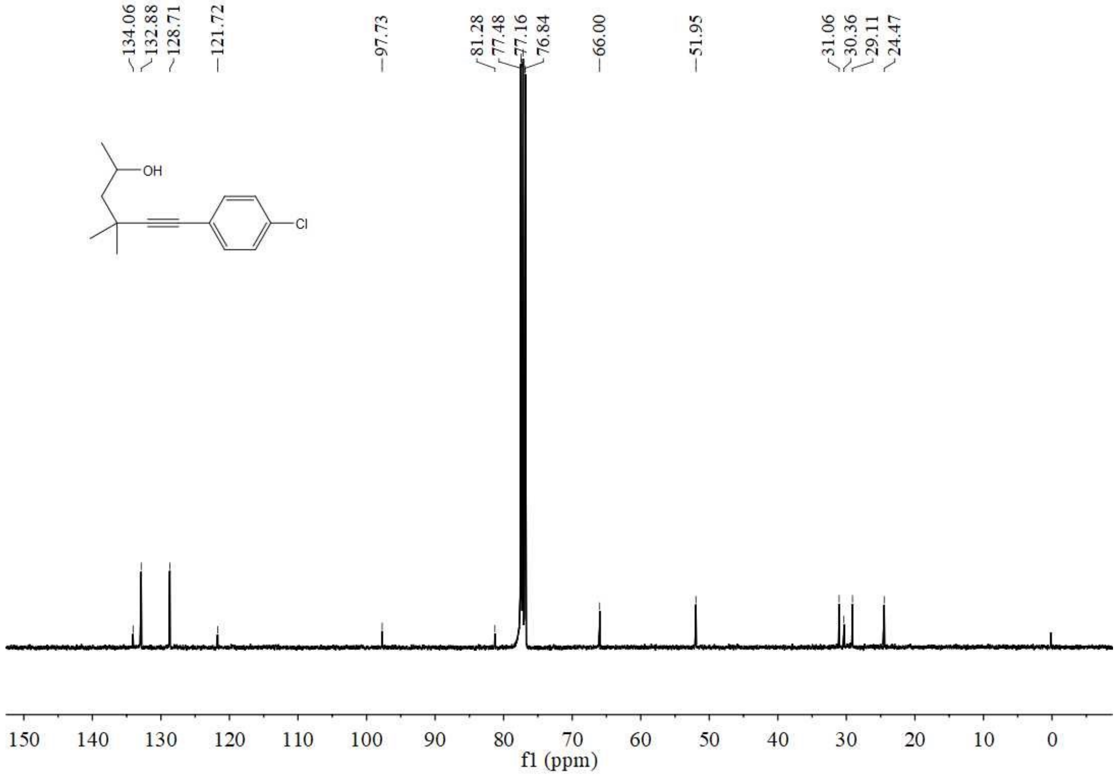

^{1}H NMR (400 MHz, $CDCl_3$) of **3f**

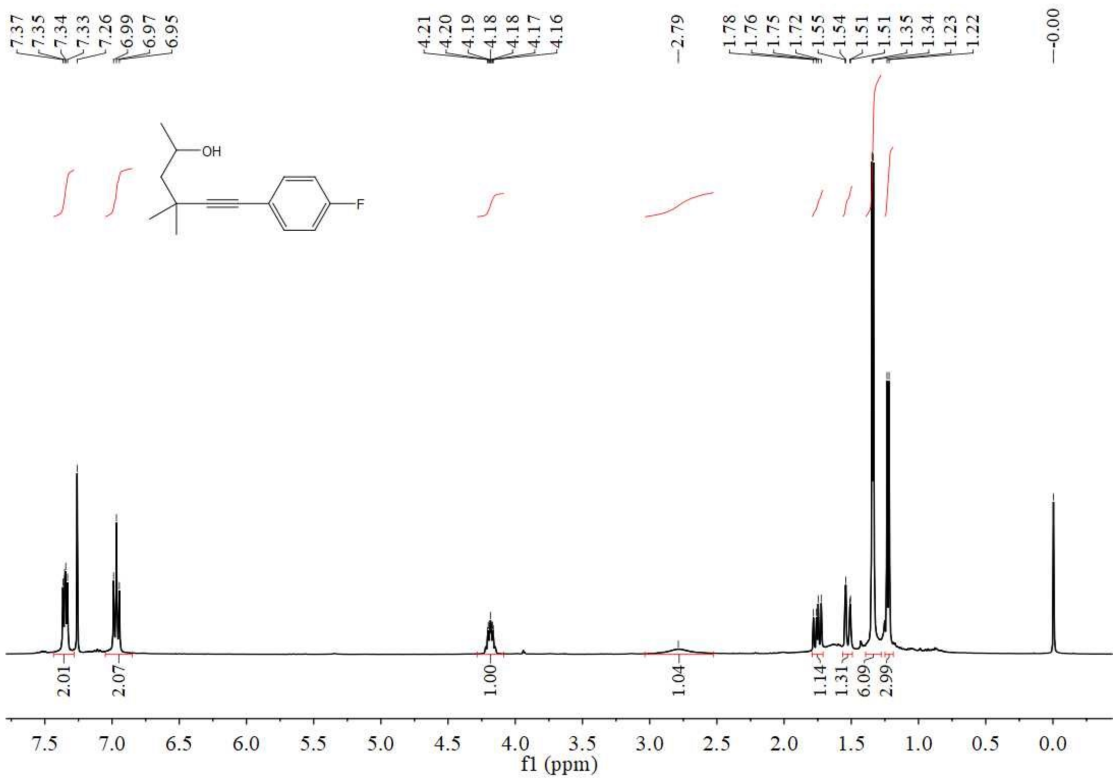

^{19}F NMR (376 MHz, $CDCl_3$) of **3f**

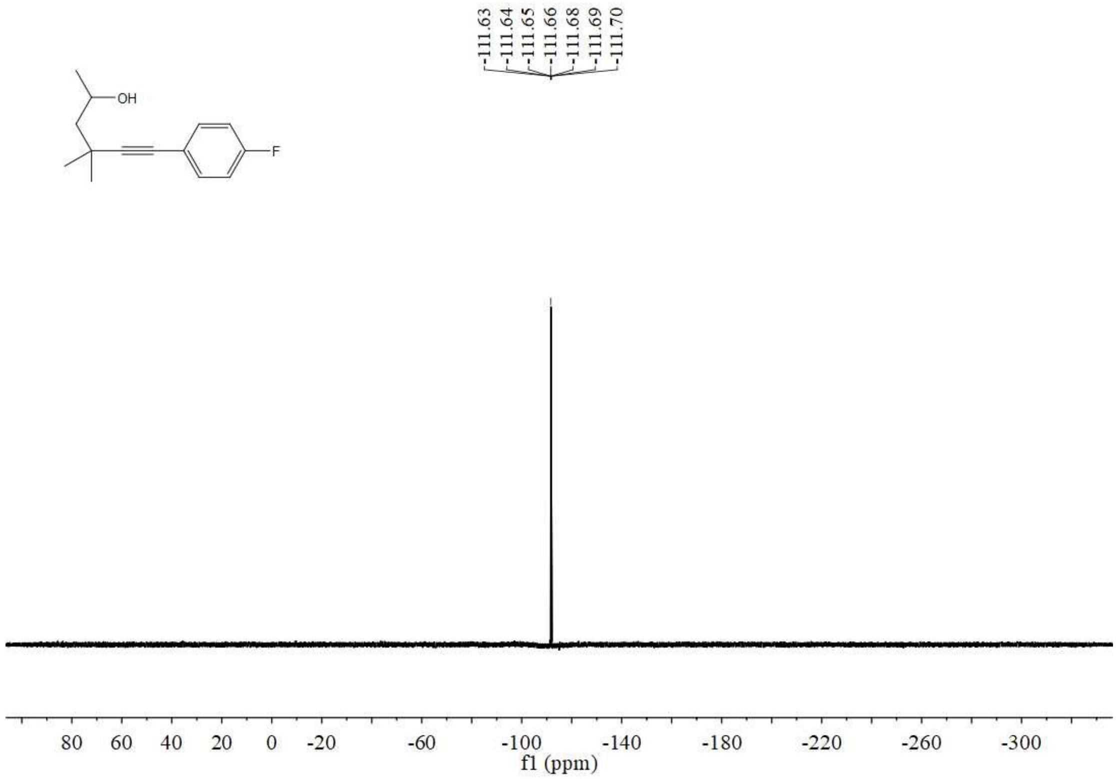

^{1}H NMR (400 MHz, $CDCl_3$) of **3g**

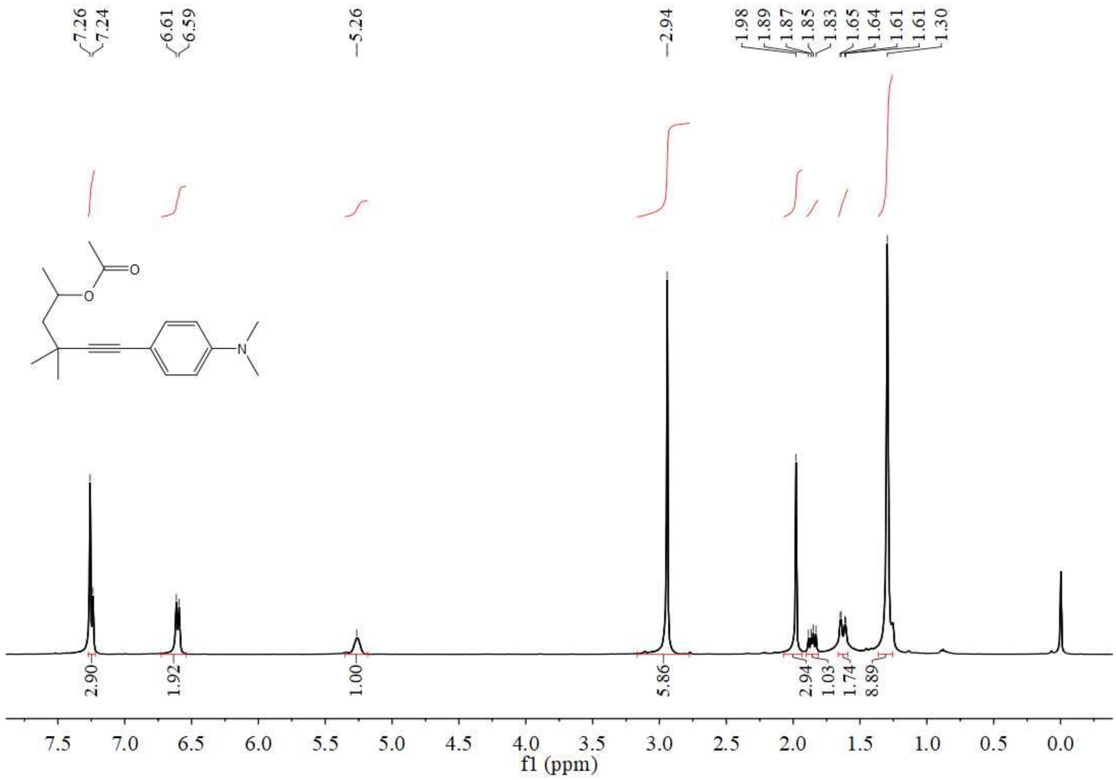

^{13}C NMR (101 MHz, CDCl$_3$) of **3g**

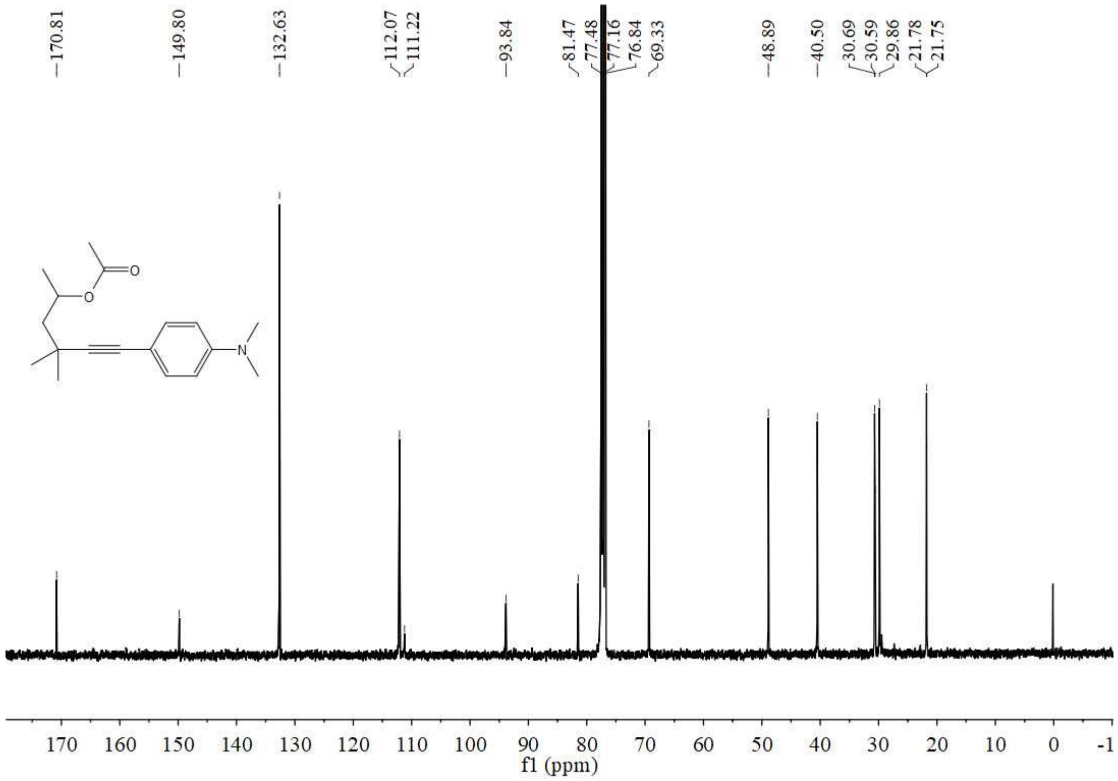

^{1}H NMR (400 MHz, CDCl$_3$) of **3h**

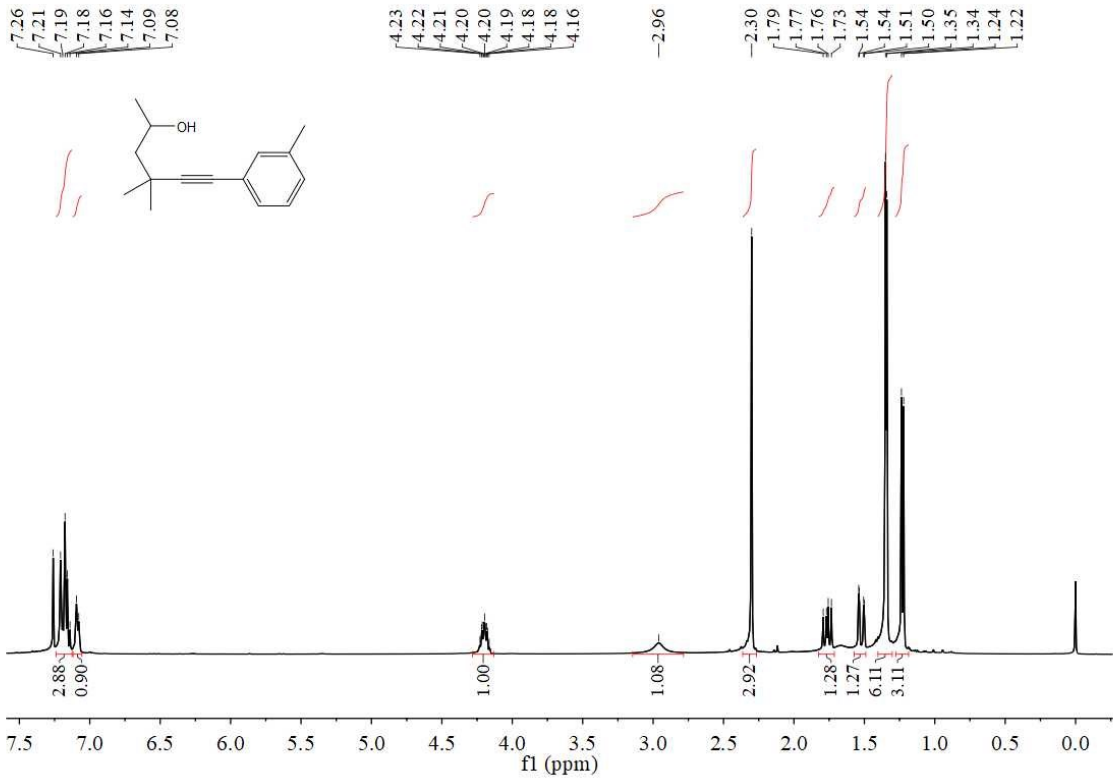

^{13}C NMR (101 MHz, CDCl3) of **3h**

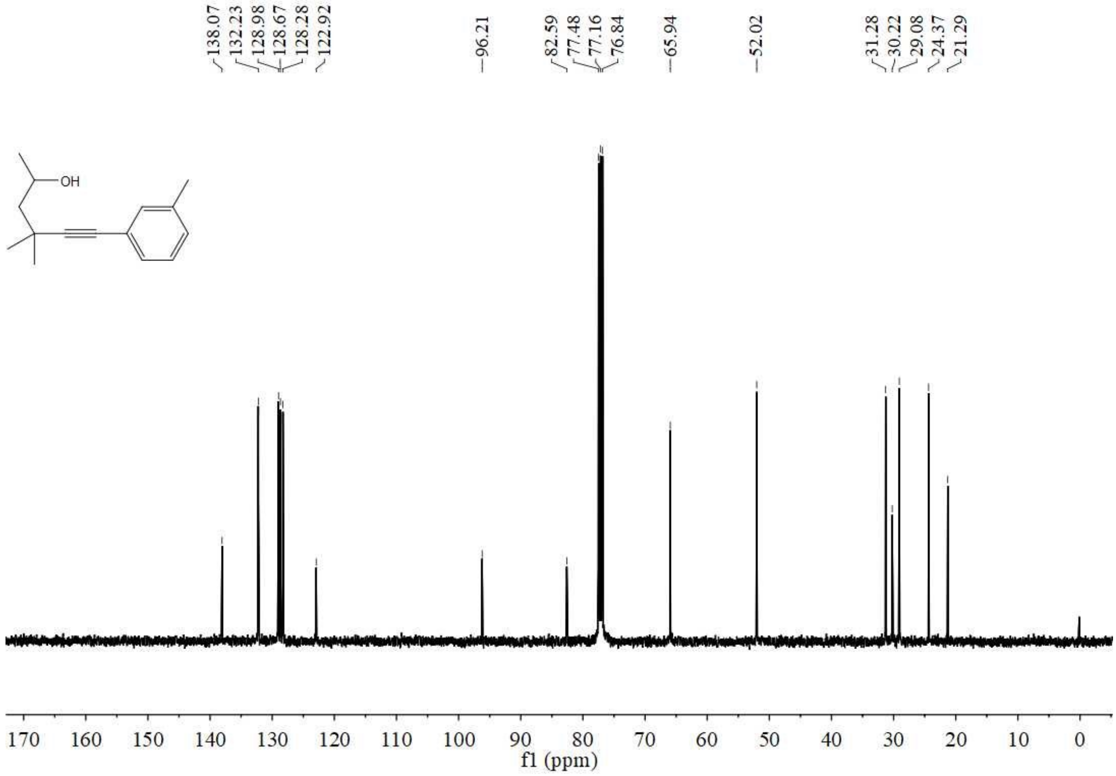

^{1}H NMR (400 MHz, CDCl3) of **3i**

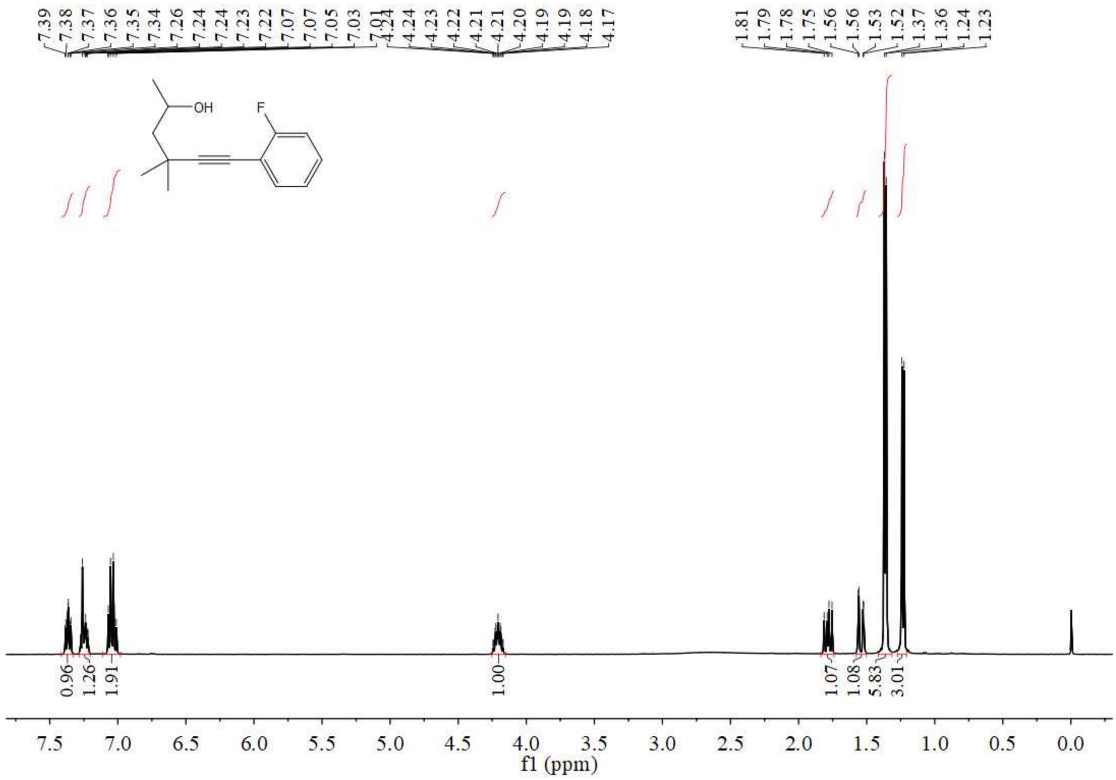

^{13}C NMR (101 MHz, $CDCl_3$) of **3i**

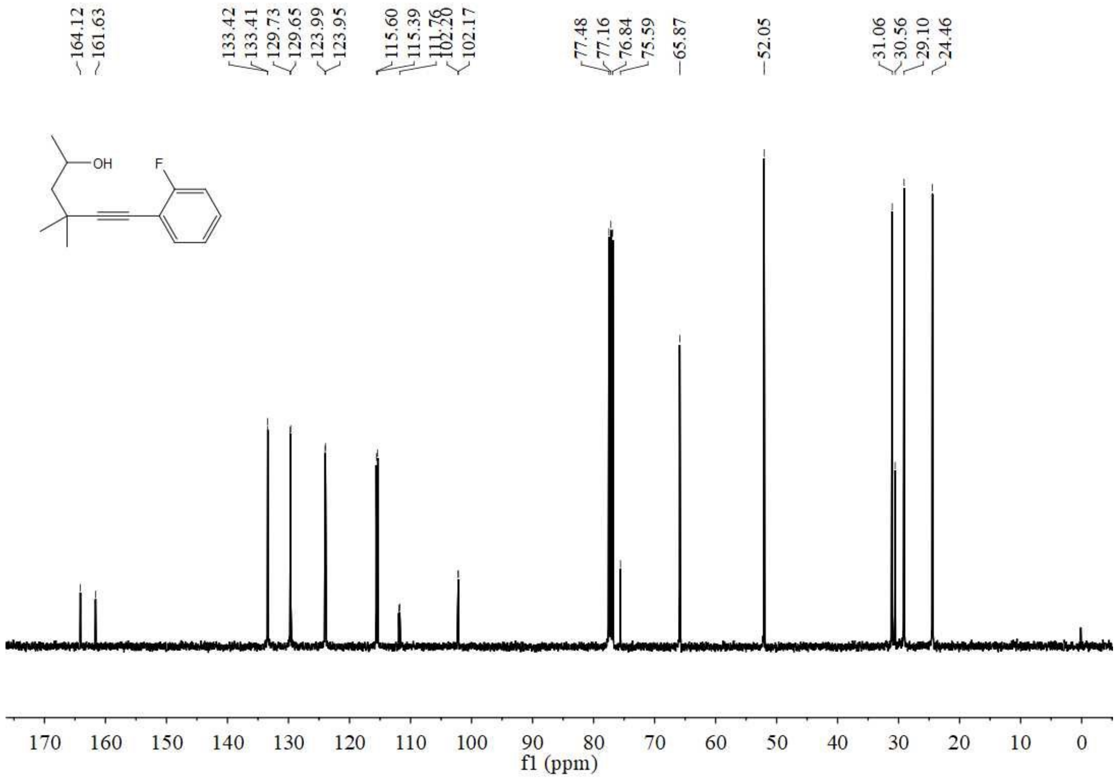

^{19}F NMR (376 MHz, $CDCl_3$) of **3i**

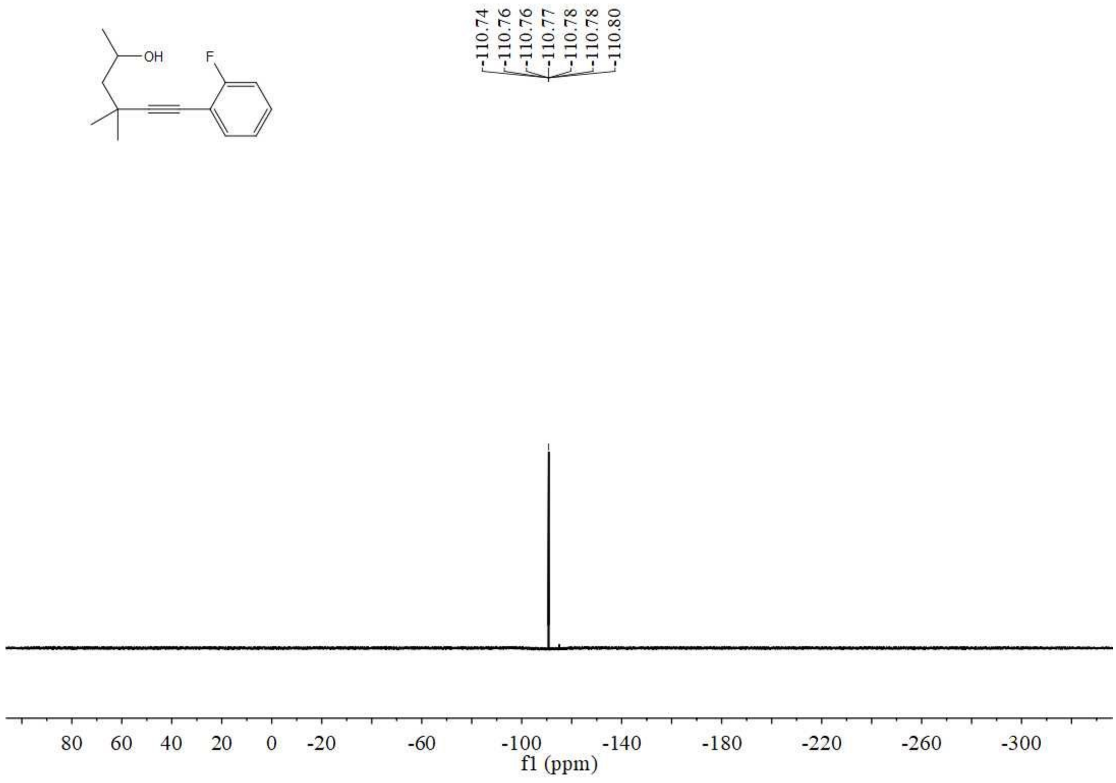

^{1}H NMR (400 MHz, $CDCl_3$) of **3j**

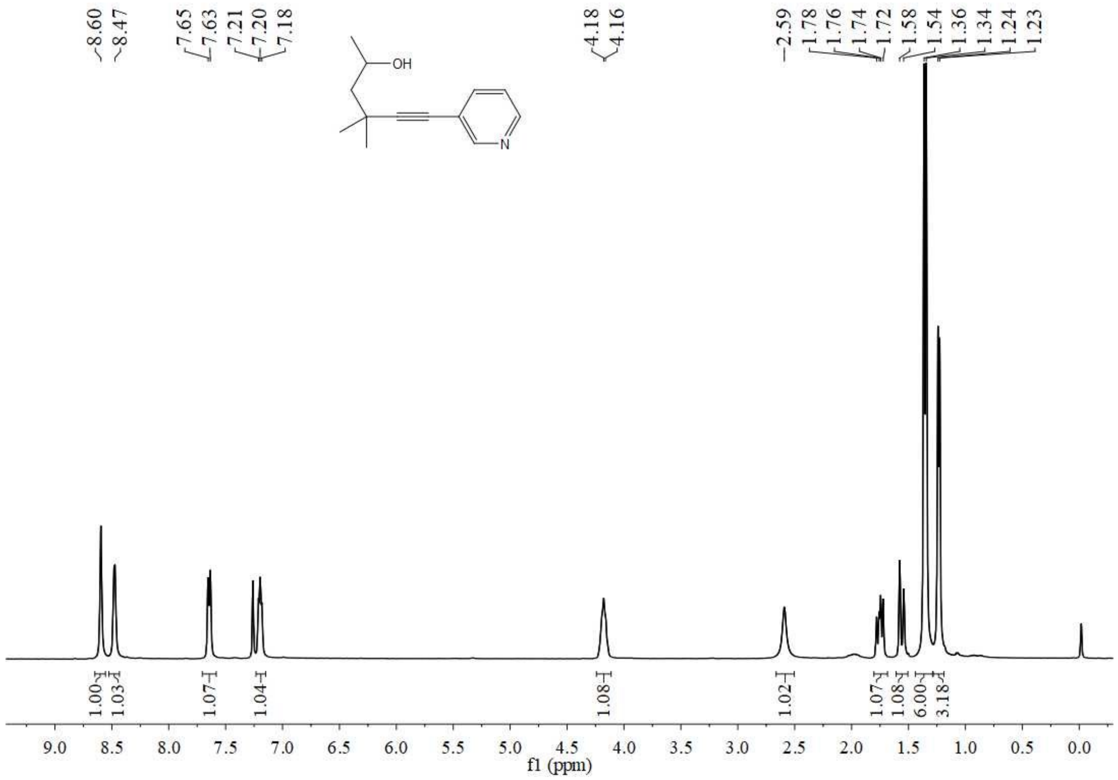

^{13}C NMR (101 MHz, $CDCl_3$) of **3j**

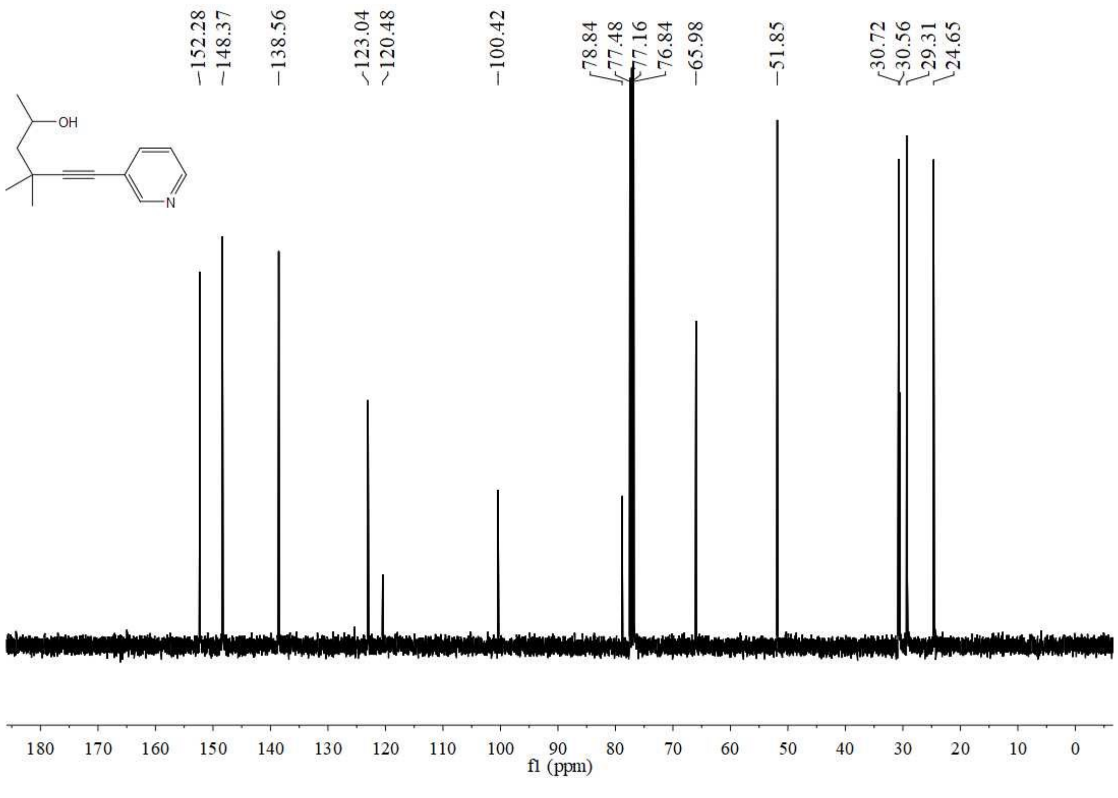

^{1}H NMR (400 MHz, $CDCl_3$) of **4a**

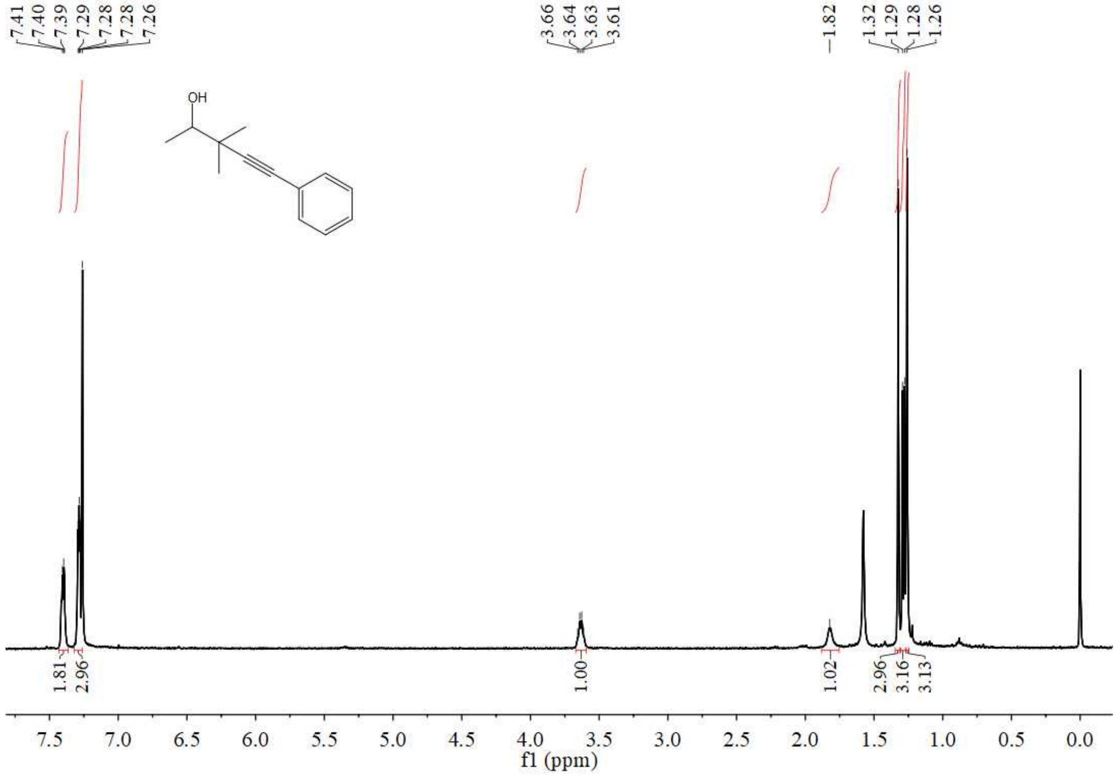

^{13}C NMR (101 MHz, $CDCl_3$) of **4a**

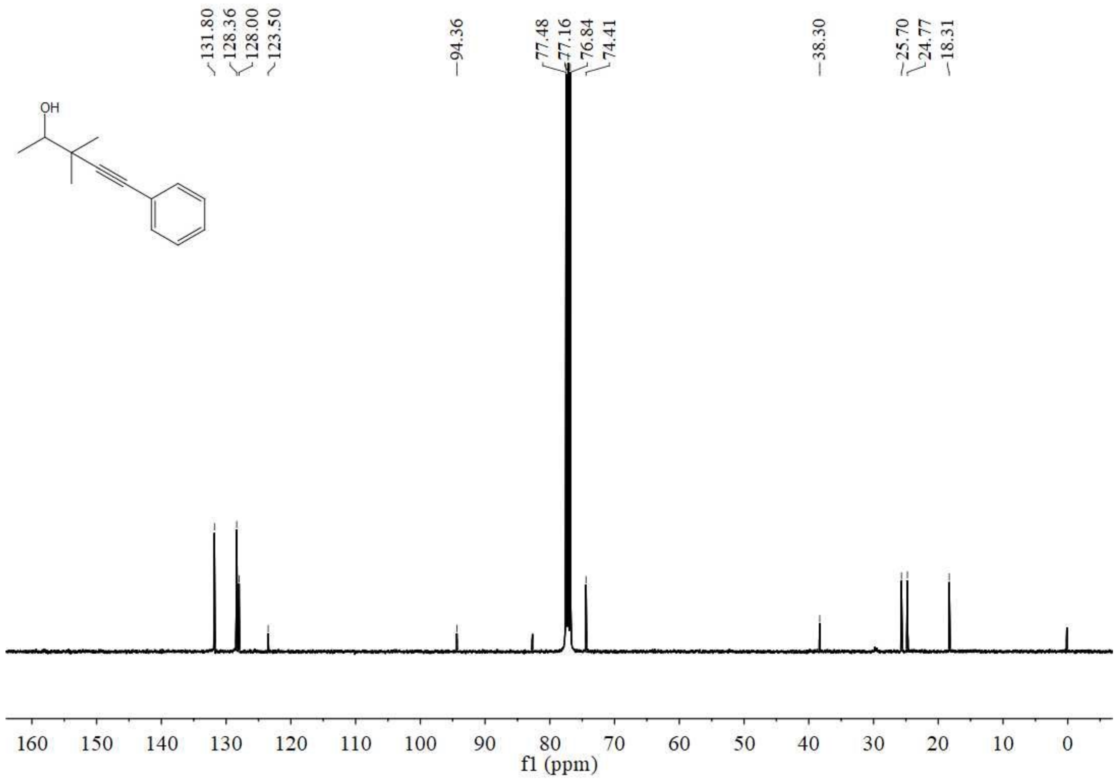

^{1}H NMR (400 MHz, $CDCl_3$) of **4b**

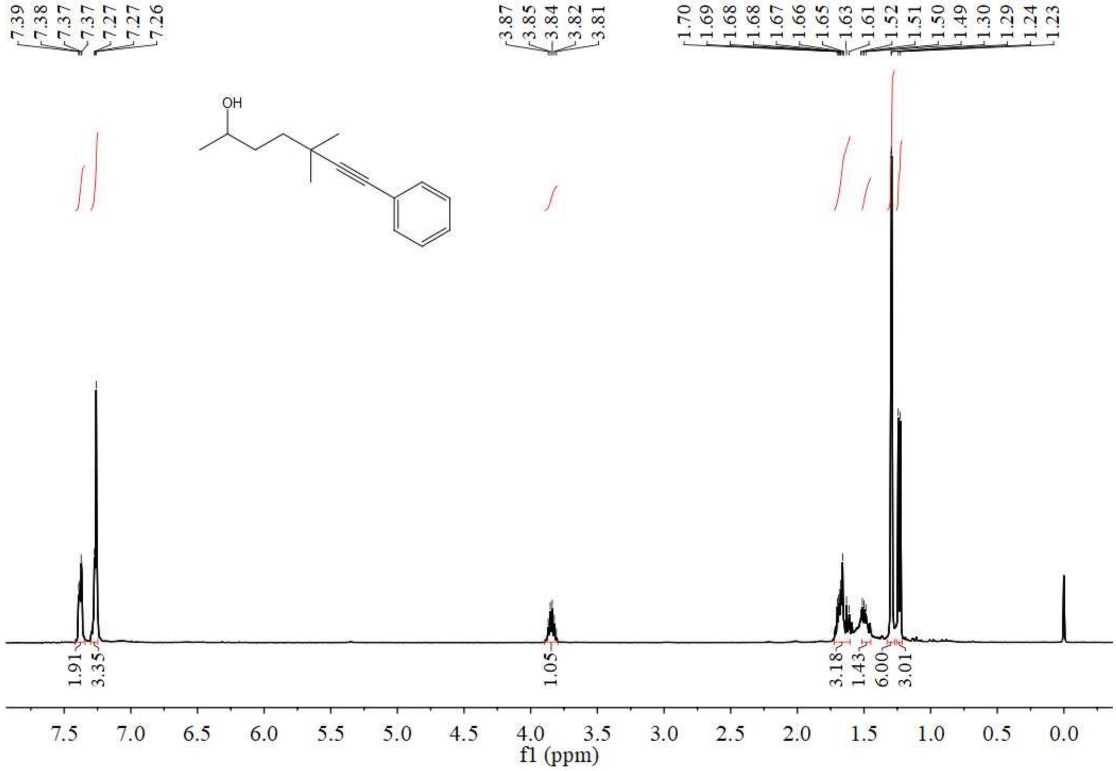

^{13}C NMR (101 MHz, $CDCl_3$) of **4b**

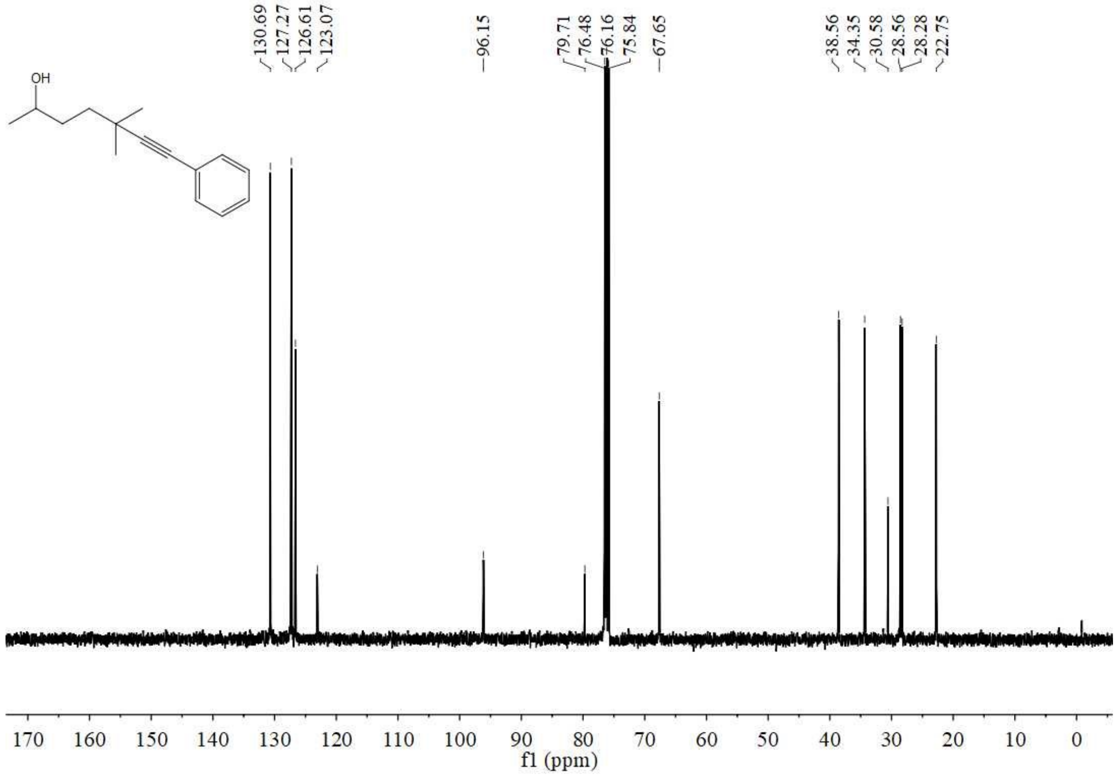

^{1}H NMR (400 MHz, $CDCl_3$) of **4c**

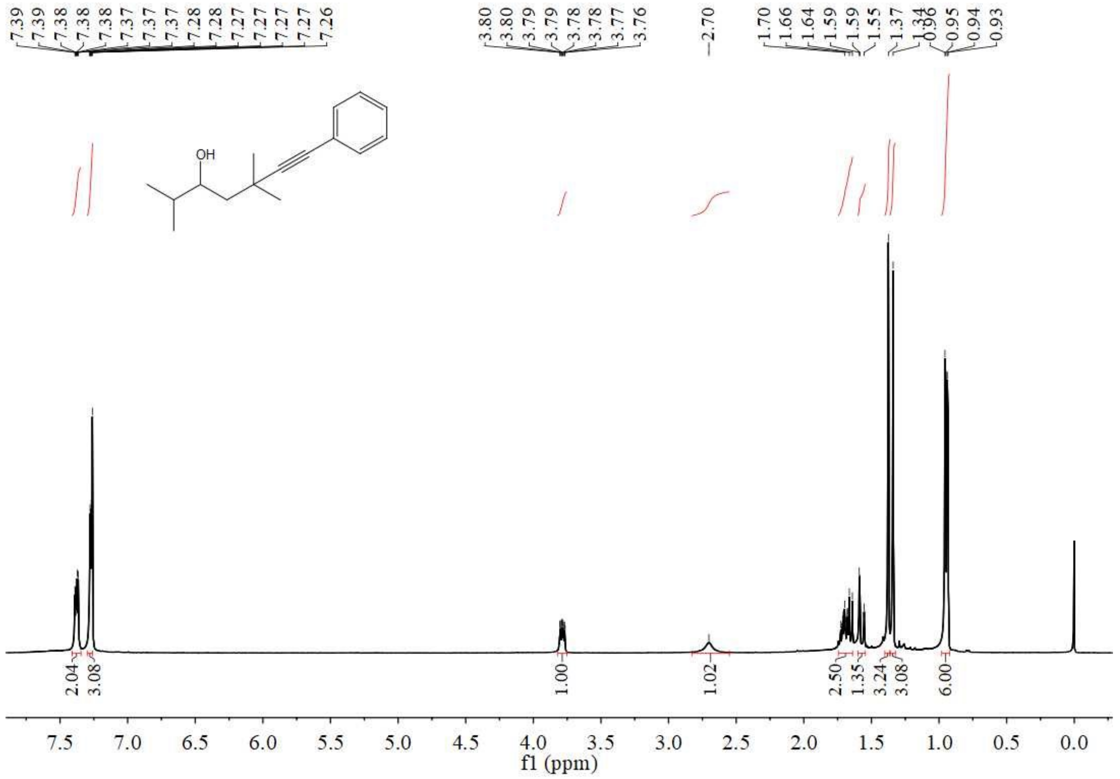

^{13}C NMR (101 MHz, $CDCl_3$) of **4c**

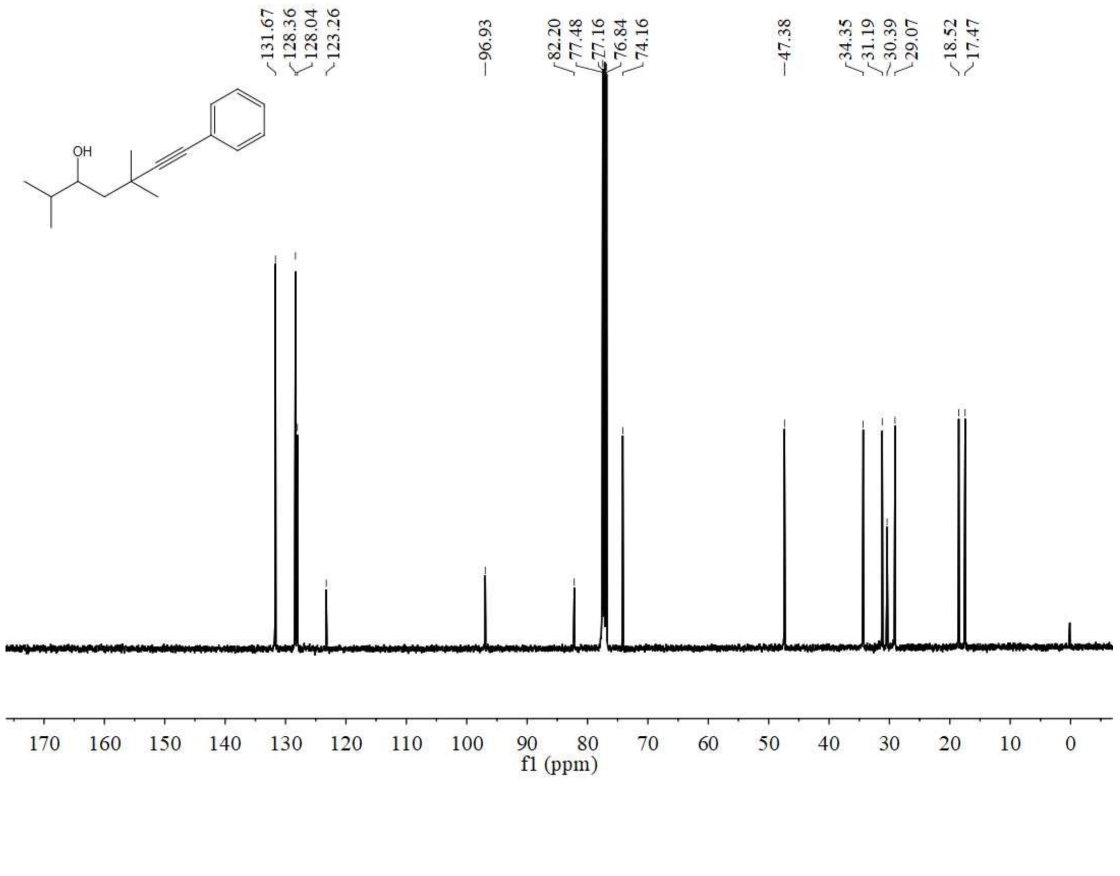

^{1}H NMR (400 MHz, $CDCl_3$) of **4d**

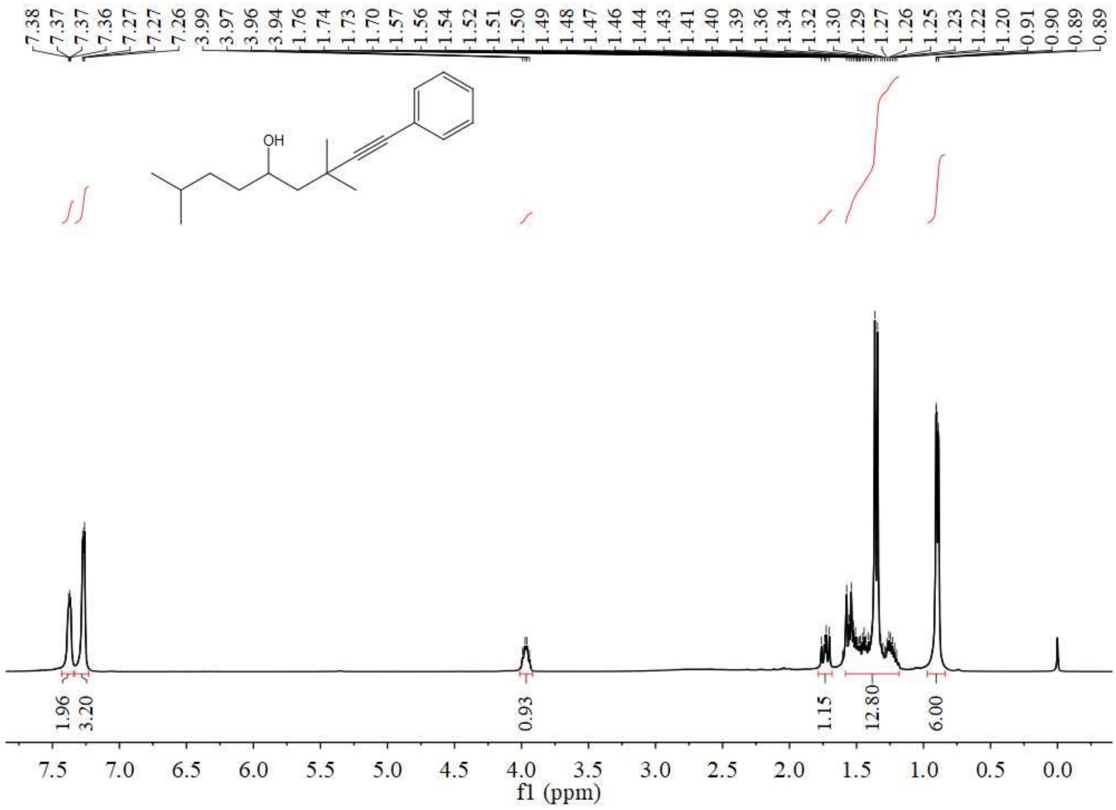

^{13}C NMR (101 MHz, $CDCl_3$) of **4d**

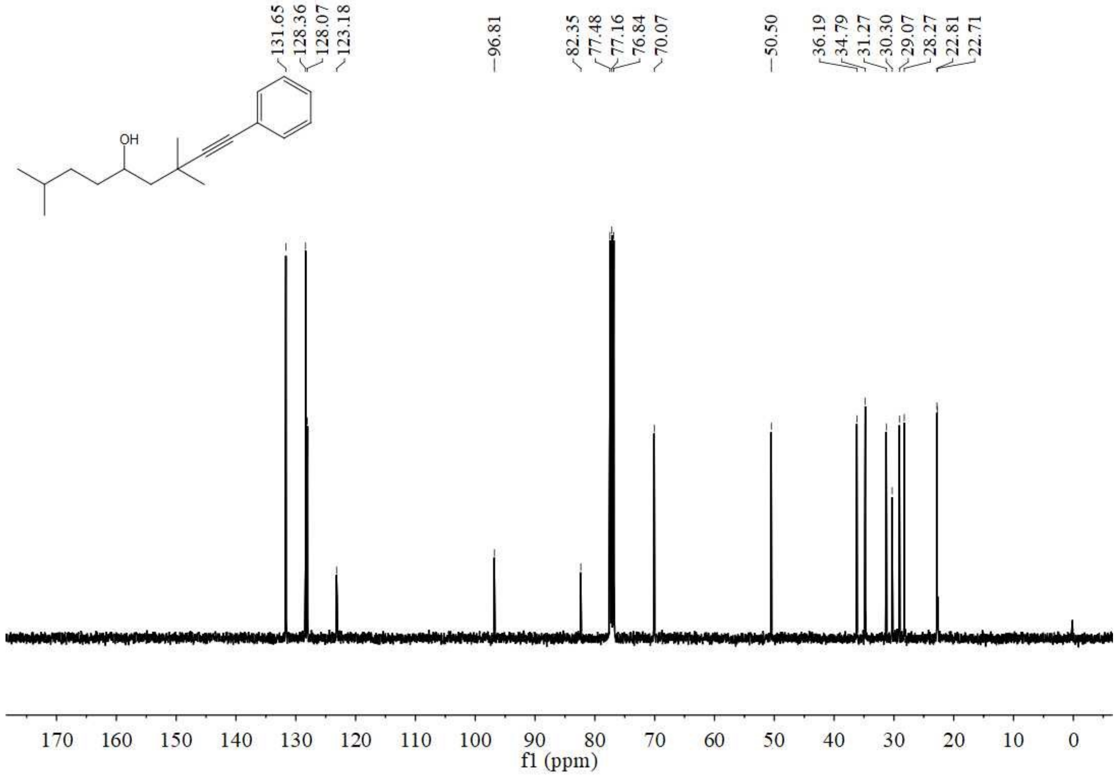

^{1}H NMR (400 MHz, $CDCl_3$) of **4e**

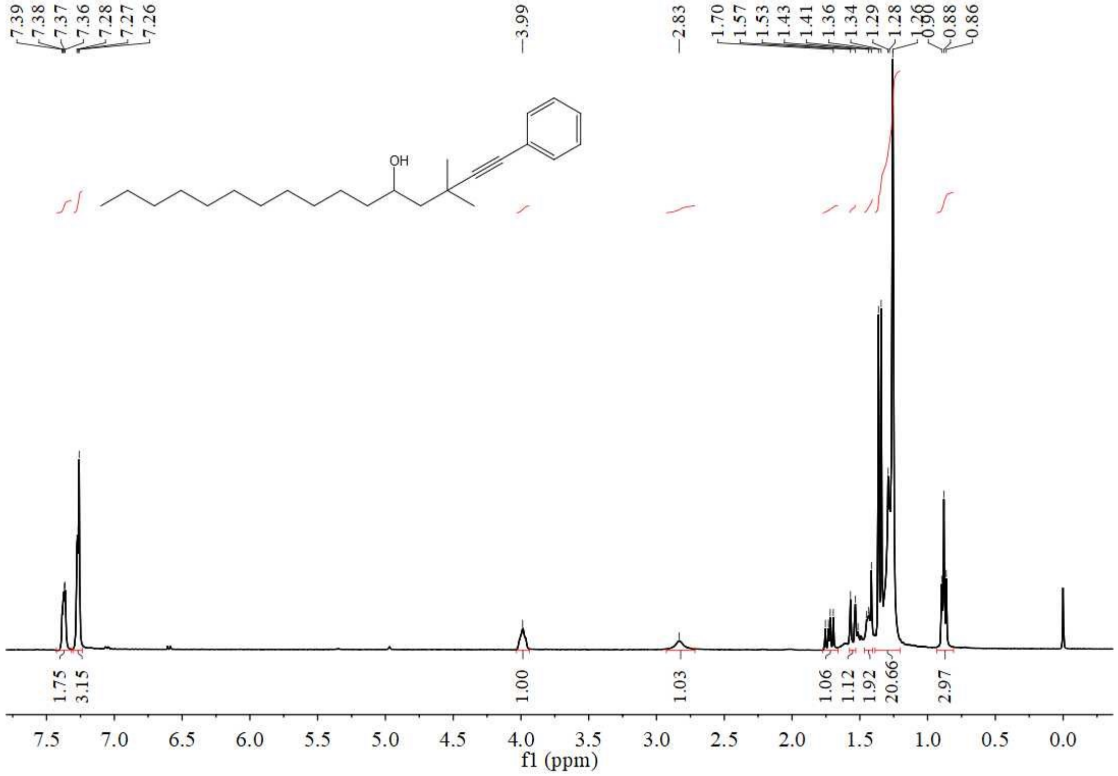

^{13}C NMR (101 MHz, $CDCl_3$) of **4e**

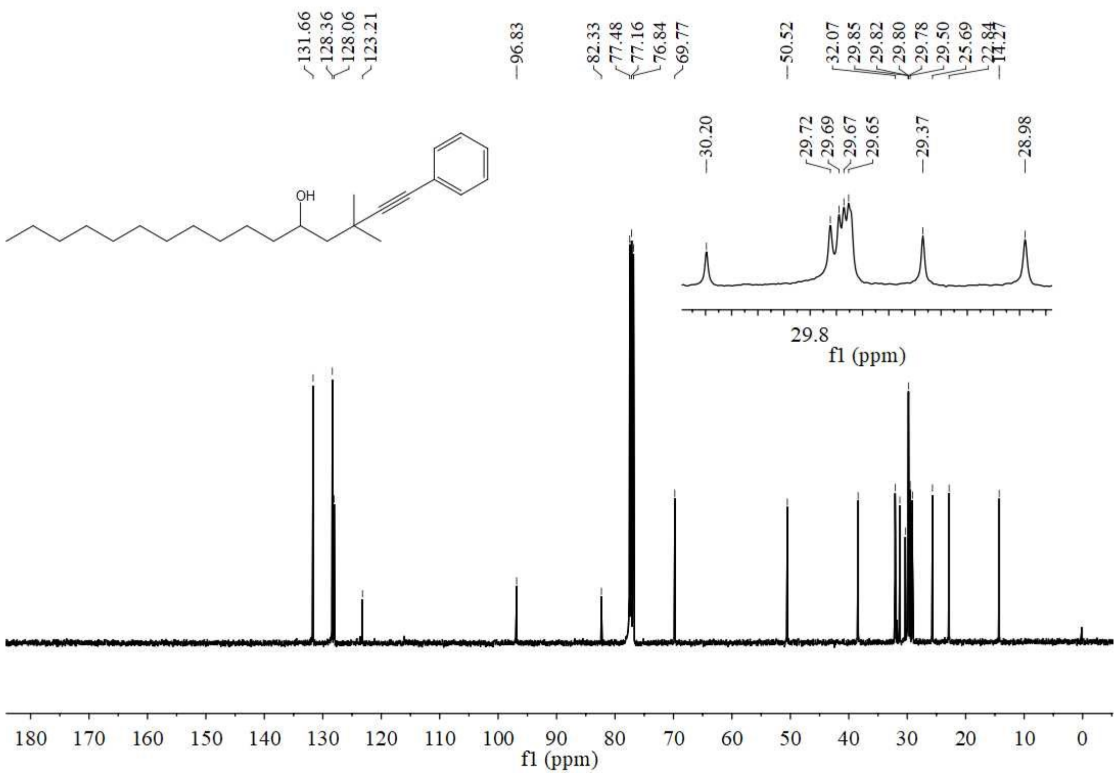

^{1}H NMR (400 MHz, $CDCl_3$) of **4f**

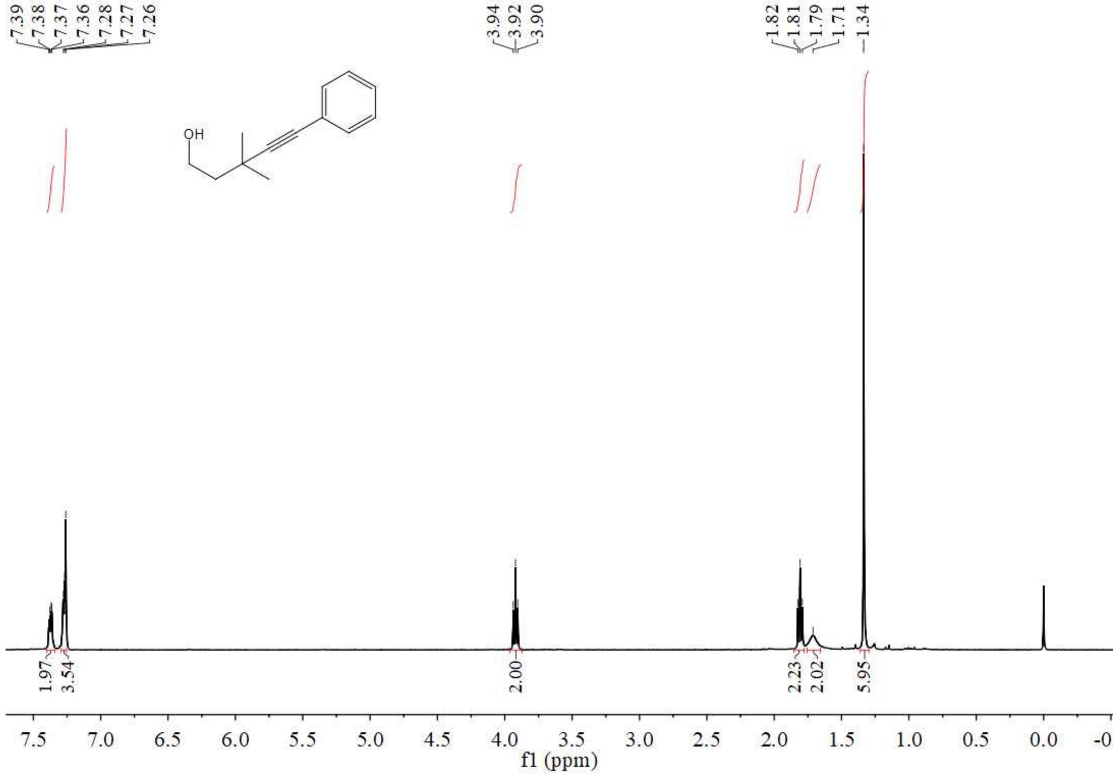

^{13}C NMR (101 MHz, $CDCl_3$) of **4f**

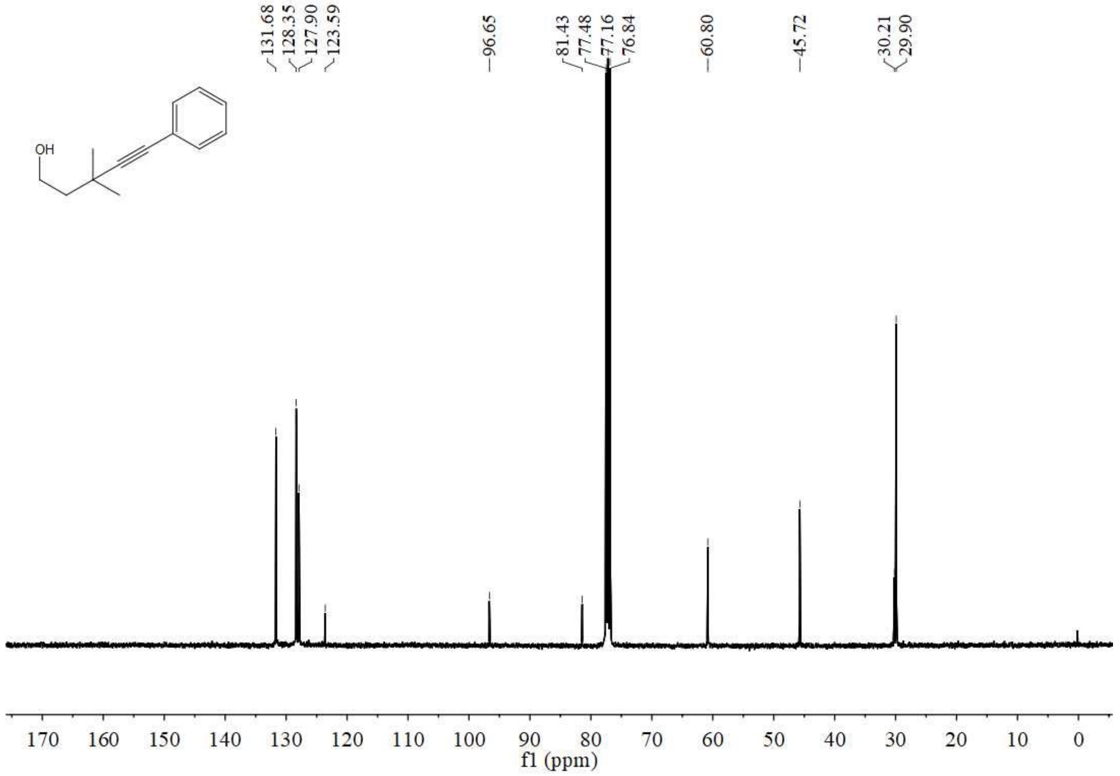

^{1}H NMR (400 MHz, $CDCl_3$) of **4g**

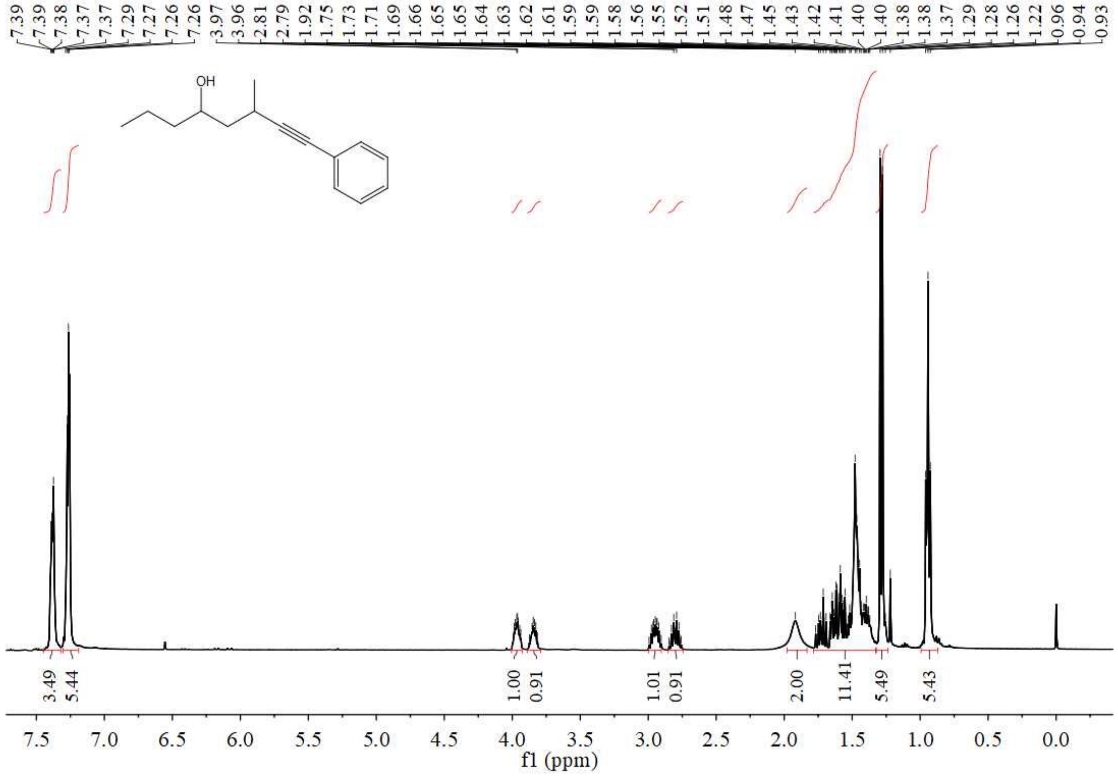

^{13}C NMR (101 MHz, $CDCl_3$) of **4g**

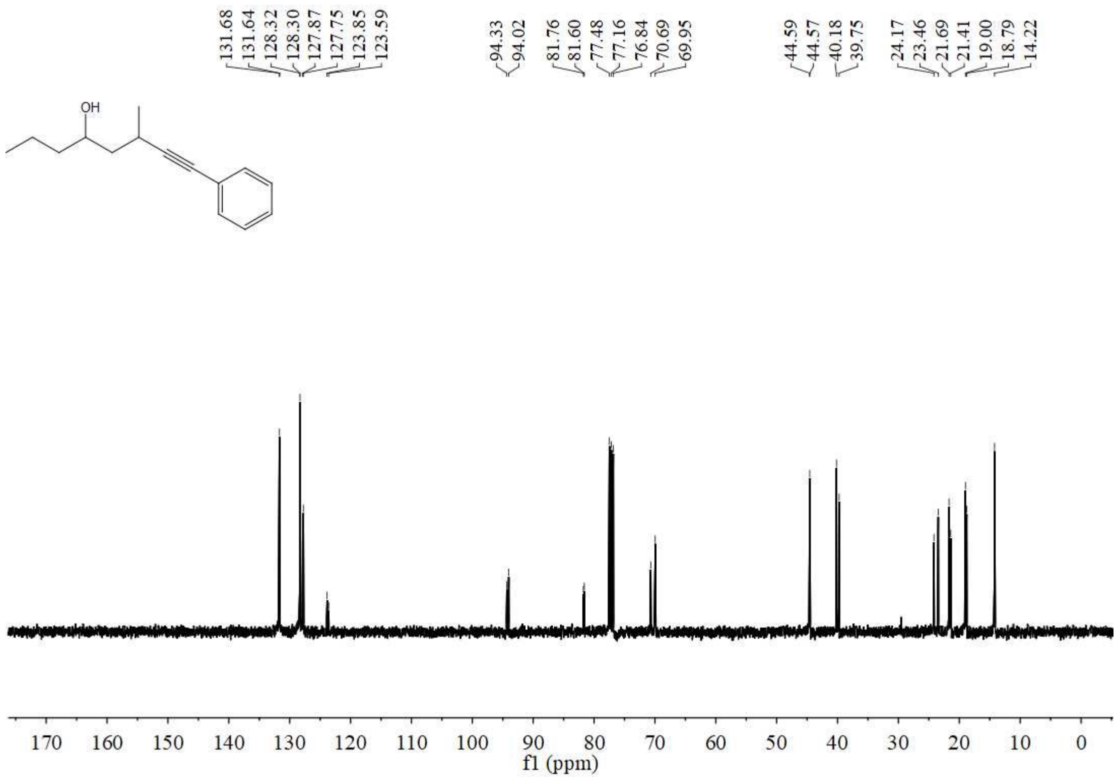

^{1}H NMR (400 MHz, $CDCl_3$) of **4h**

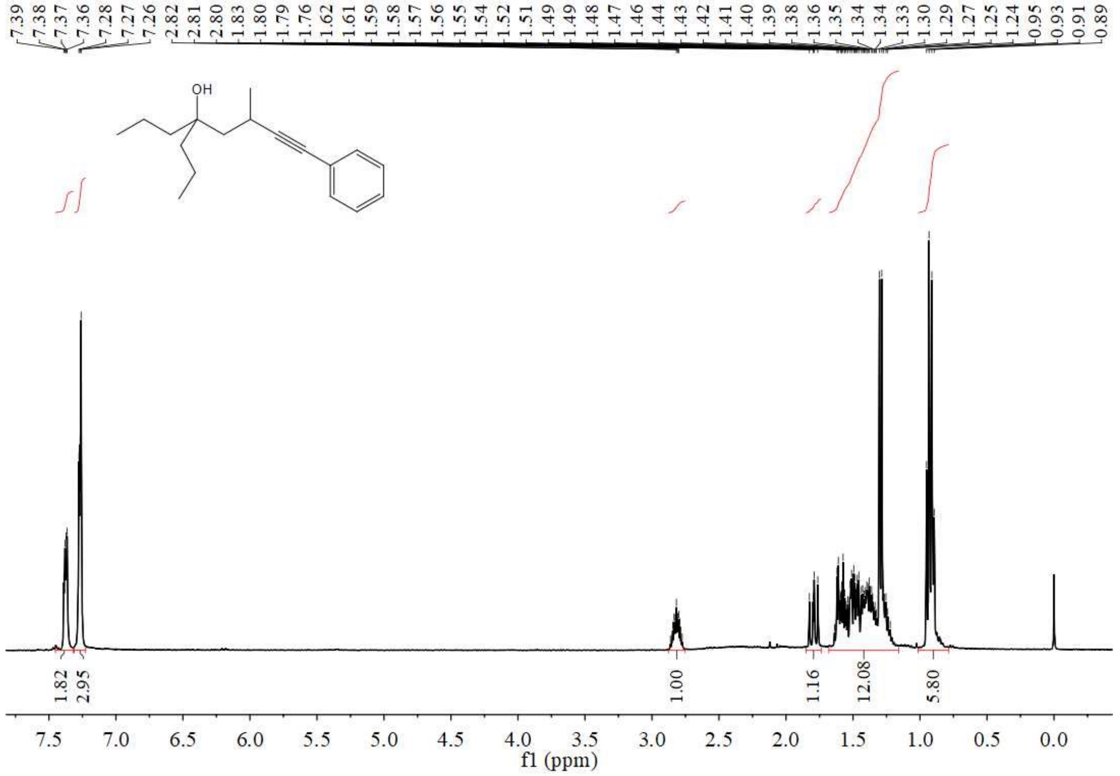

^{13}C NMR (101 MHz, $CDCl_3$) of **4h**

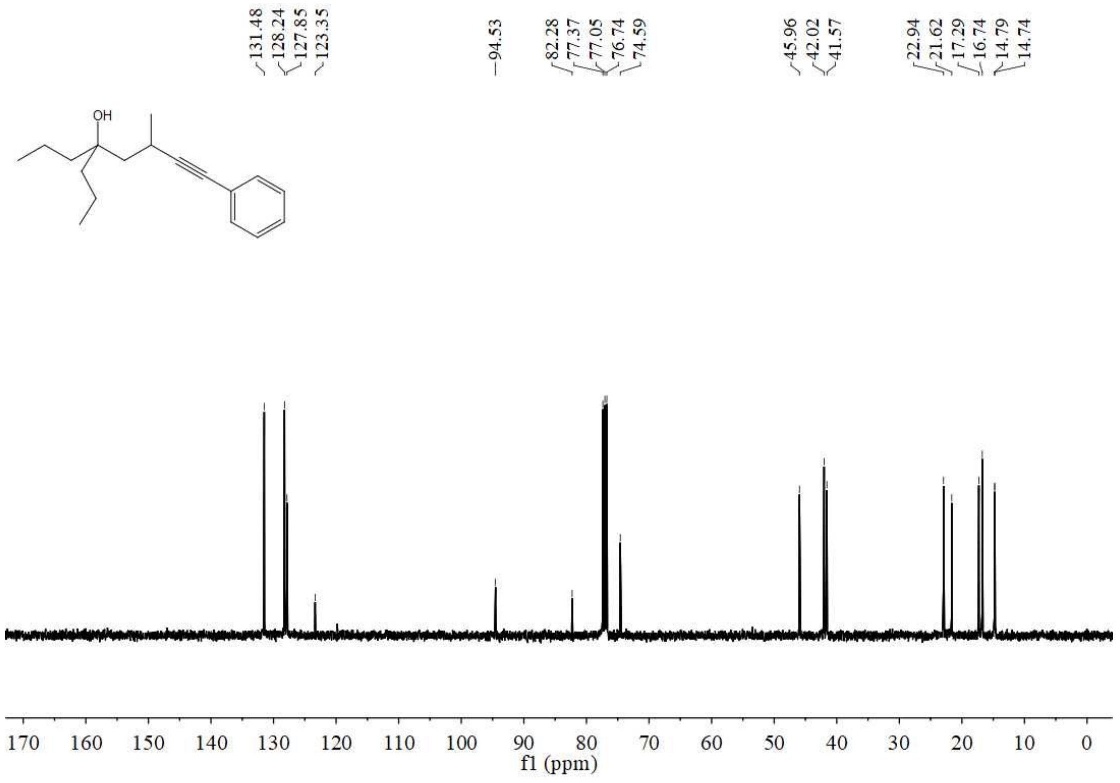

^{1}H NMR (400 MHz, $CDCl_3$) of **4i**

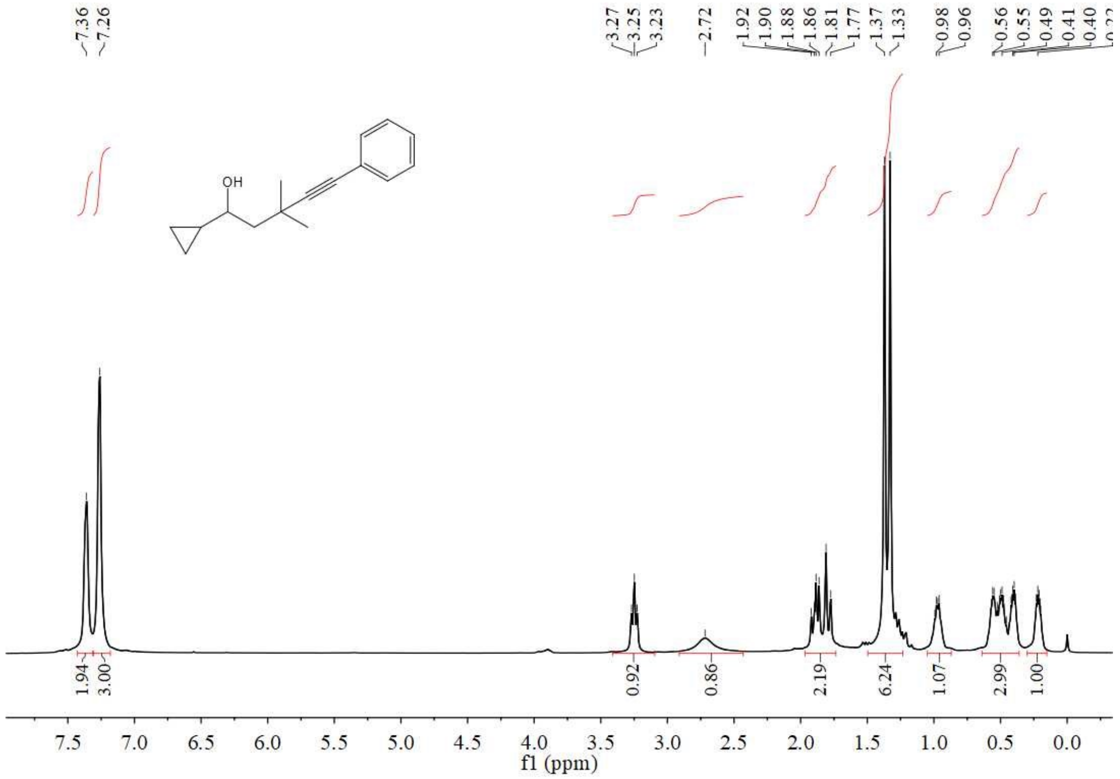

^{13}C NMR (101 MHz, $CDCl_3$) of **4i**

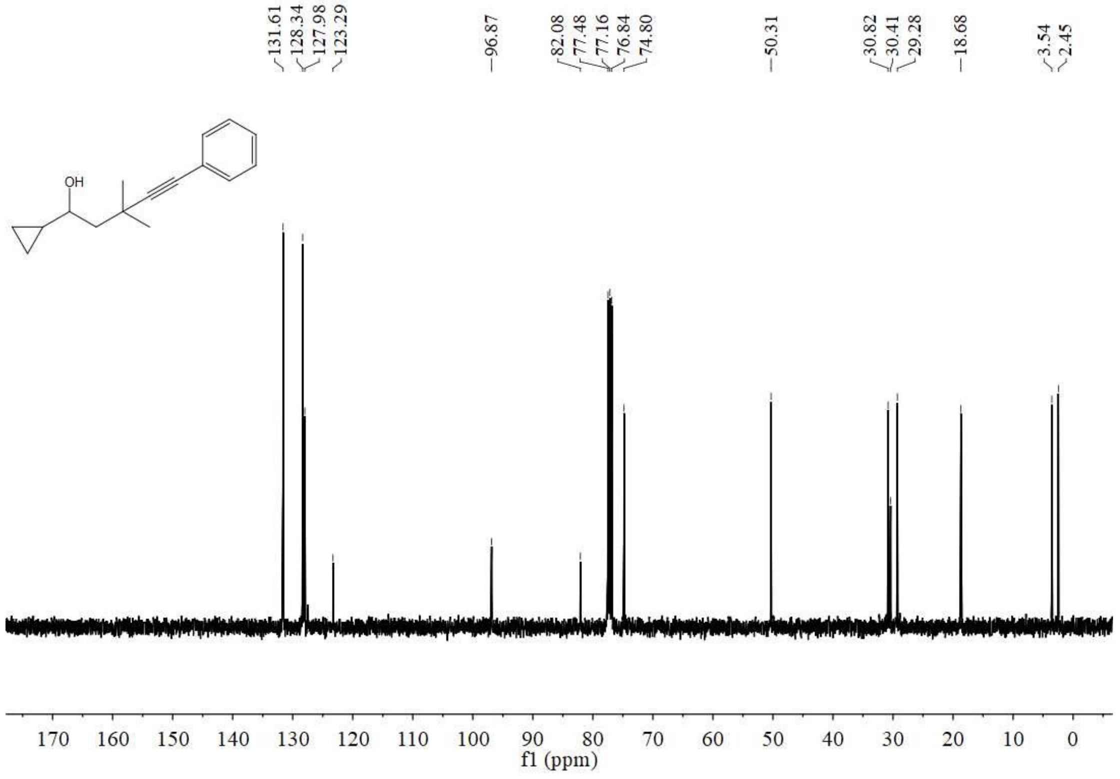

^{1}H NMR (400 MHz, $CDCl_3$) of **4j**

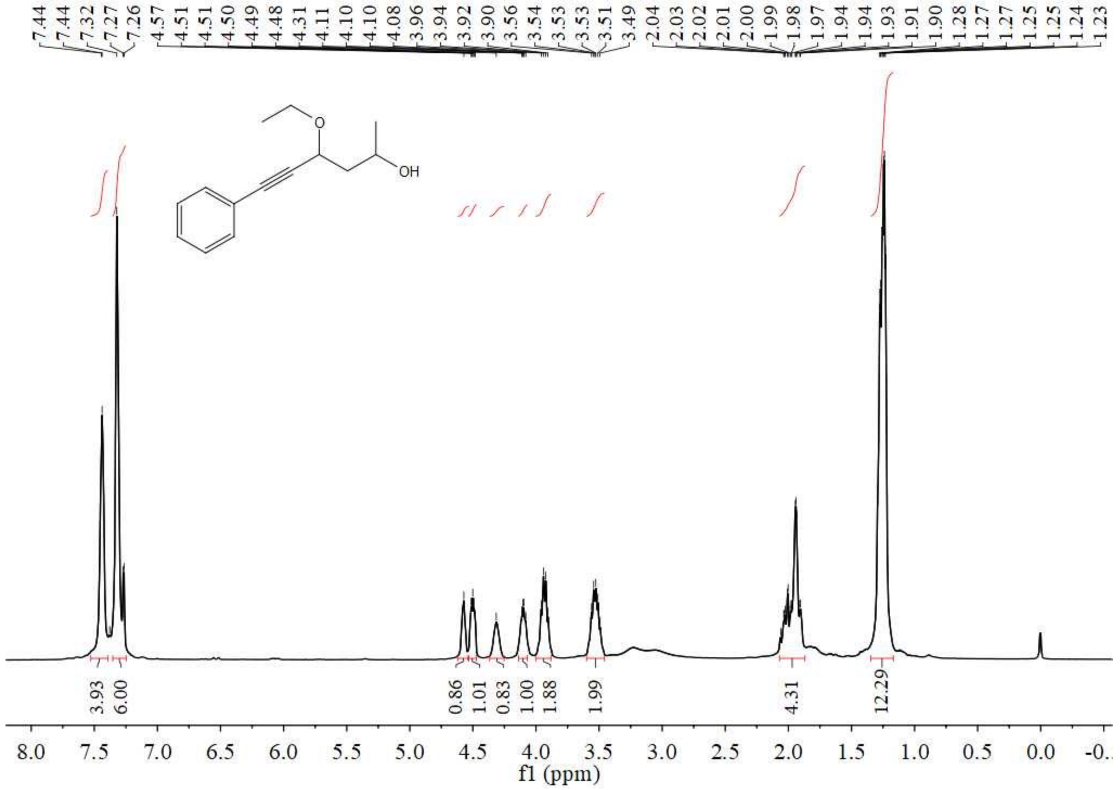

^{13}C NMR (101 MHz, $CDCl_3$) of **4j**

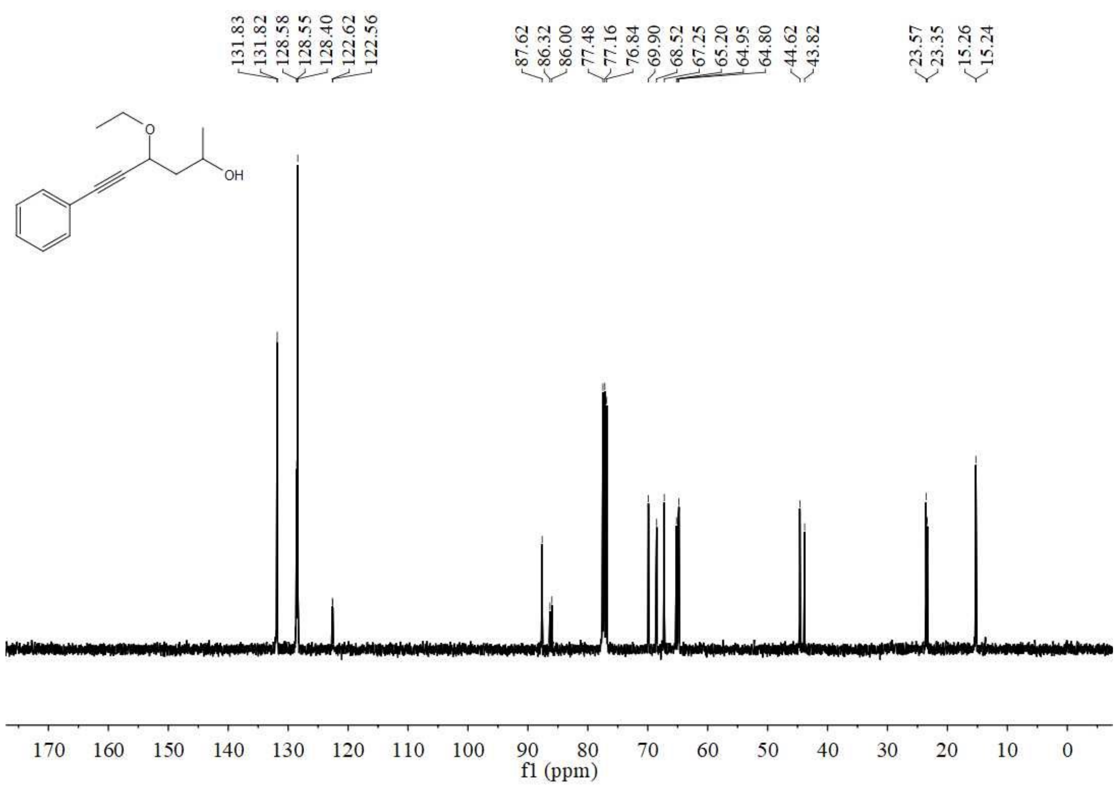

^{1}H NMR (400 MHz, $CDCl_3$) of **4k**

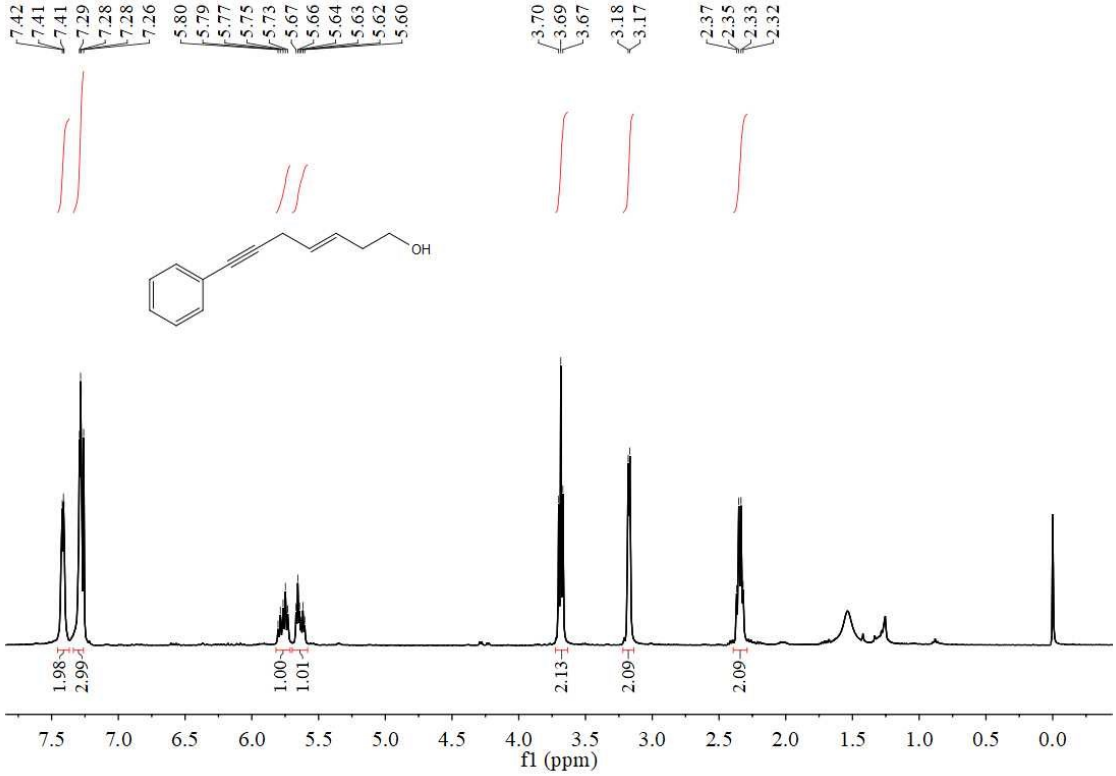

^{13}C NMR (101 MHz, $CDCl_3$) of **4k**

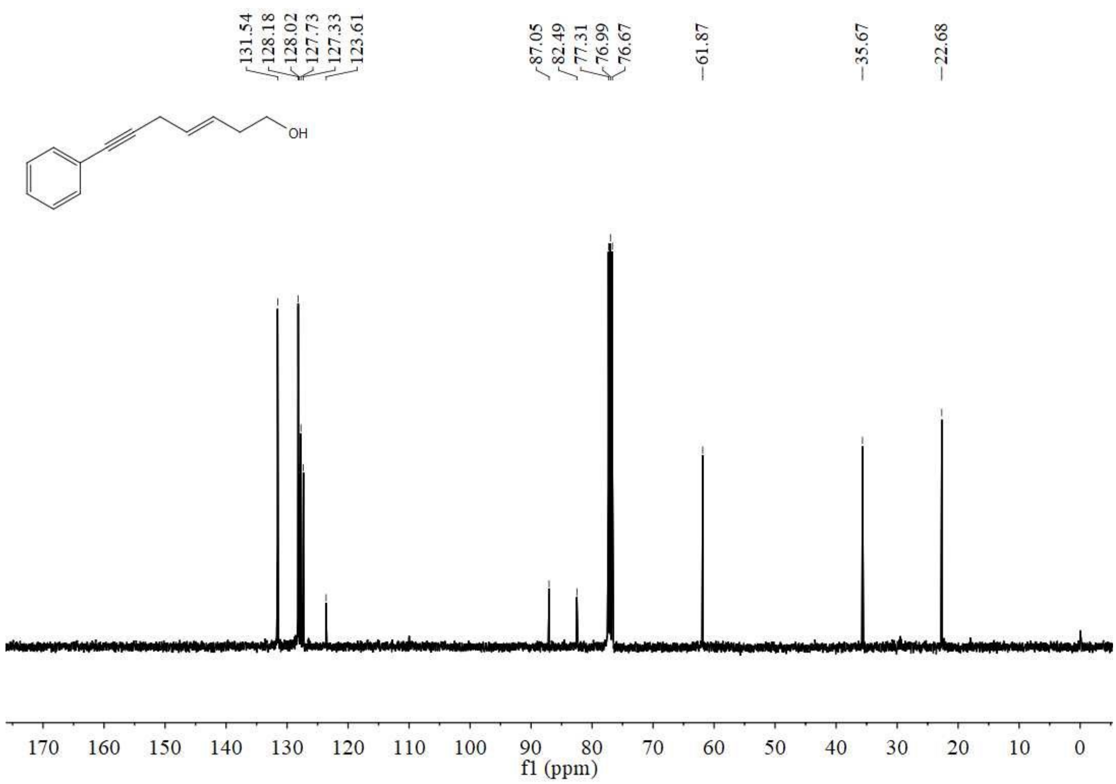

^{1}H NMR (400 MHz, $CDCl_3$) of **4l**

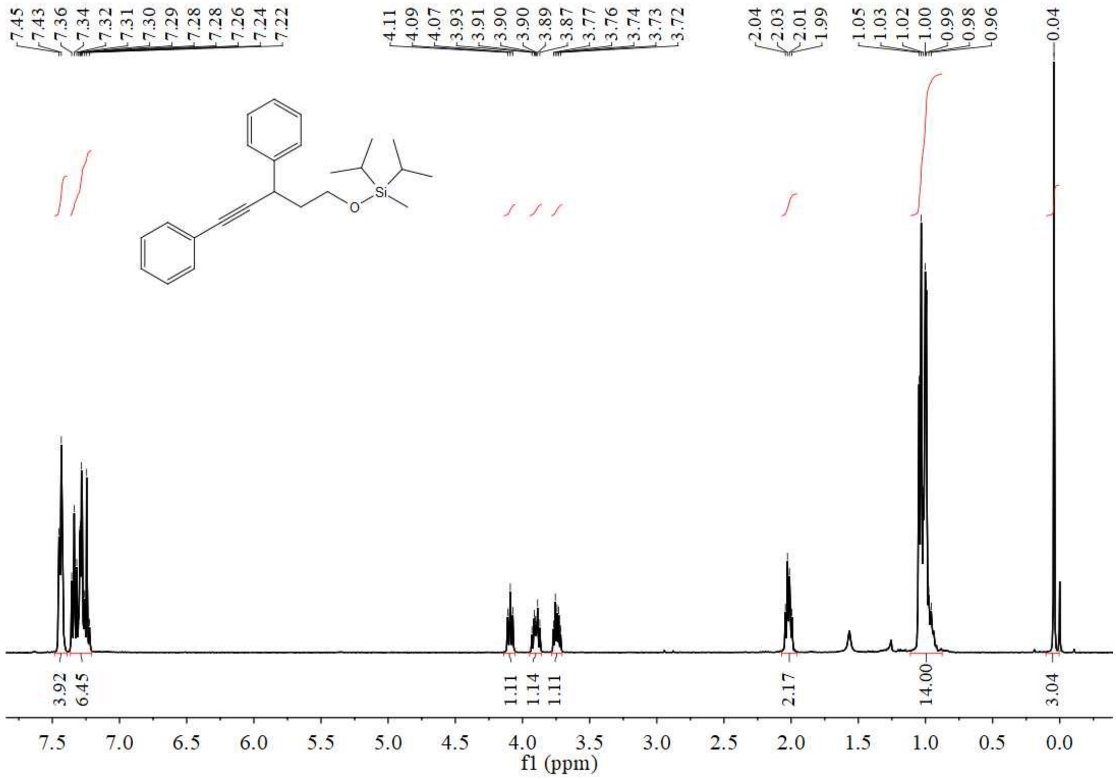

^{13}C NMR (101 MHz, $CDCl_3$) of **4l**

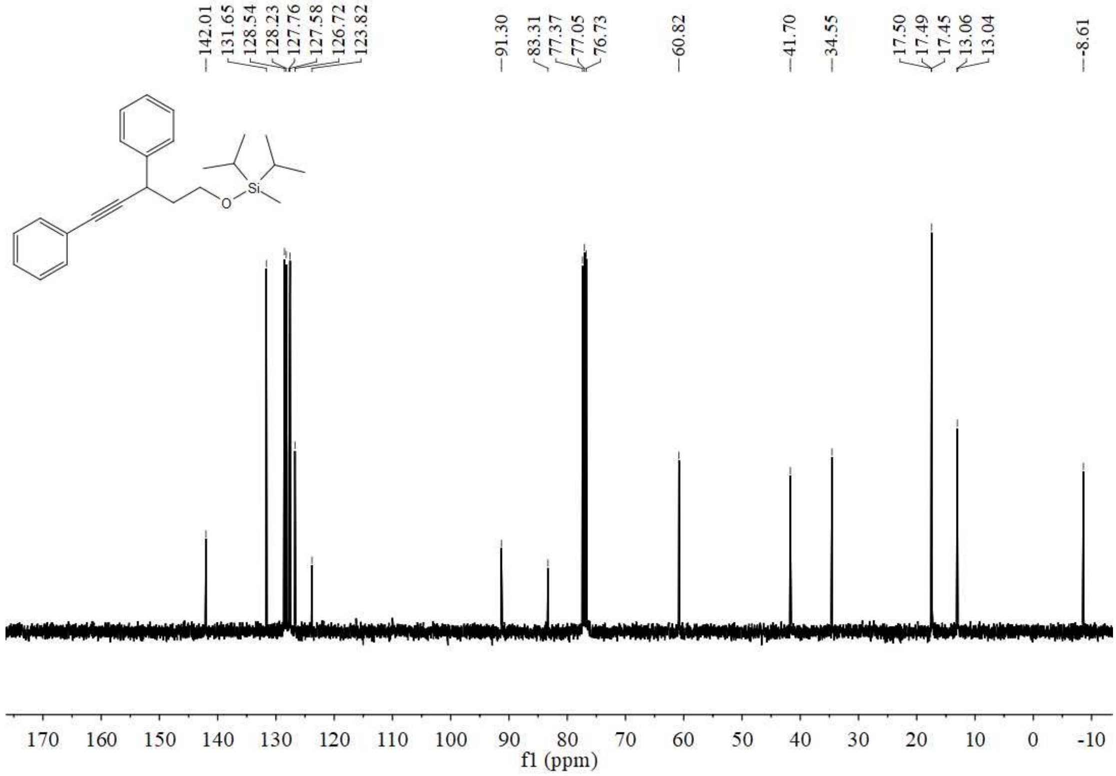

^{1}H NMR (400 MHz, CDCl3) of **4m**

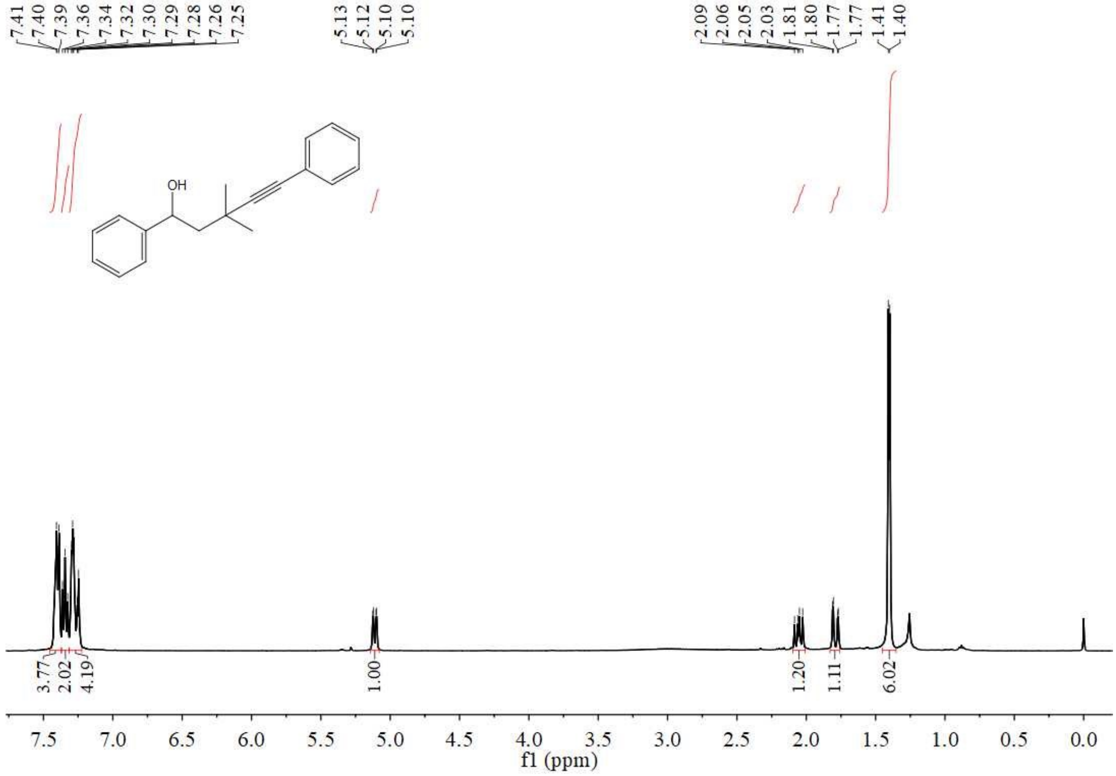

^{13}C NMR (101 MHz, CDCl3) of **4m**

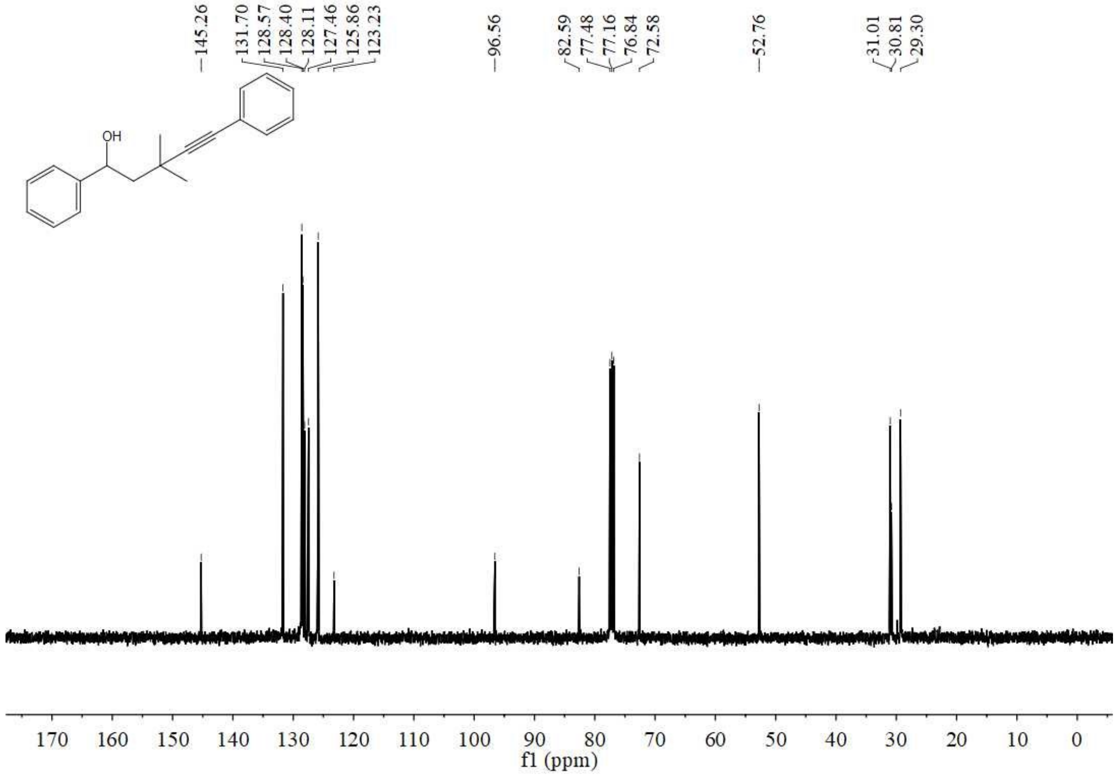

^{1}H NMR (400 MHz, $CDCl_3$) of **4n**

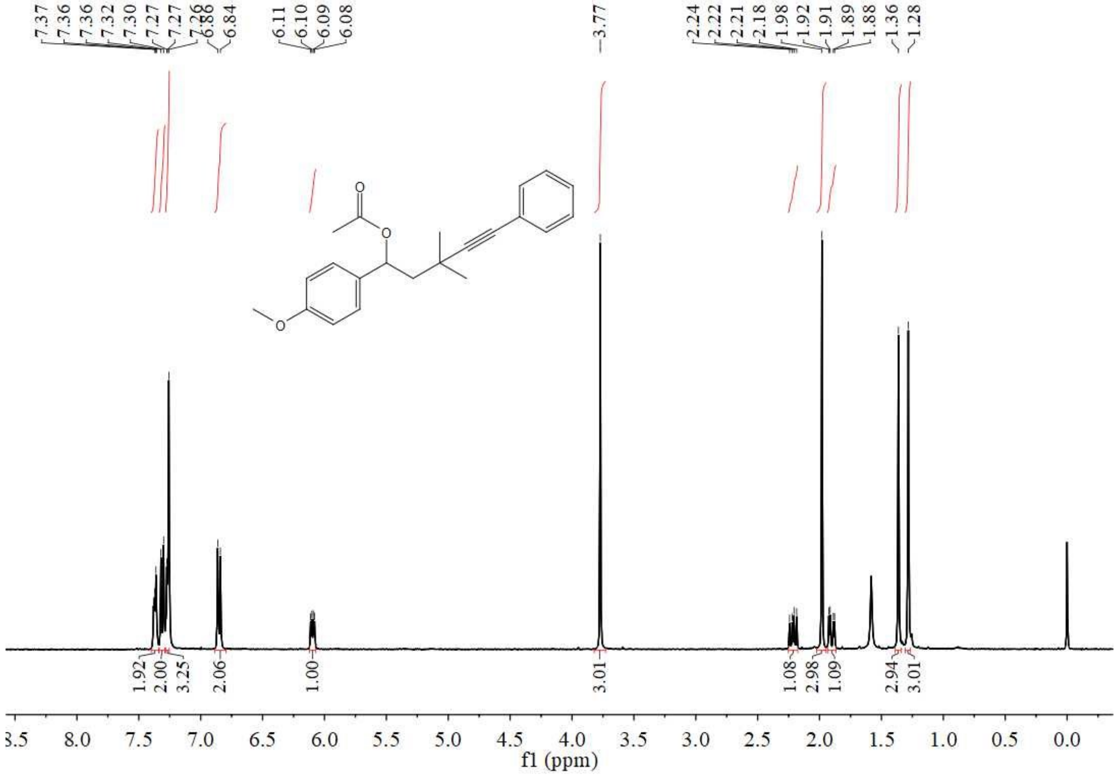

^{13}C NMR (101 MHz, $CDCl_3$) of **4n**

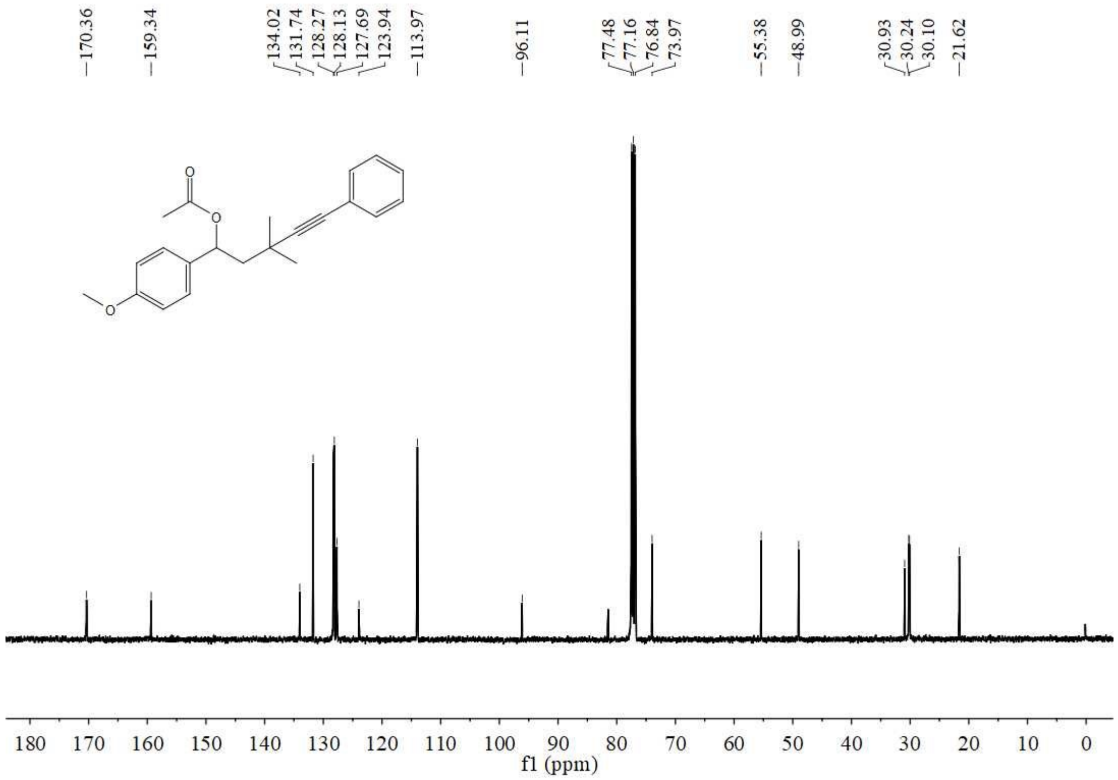

^{1}H NMR (400 MHz, $CDCl_3$) of **4o**

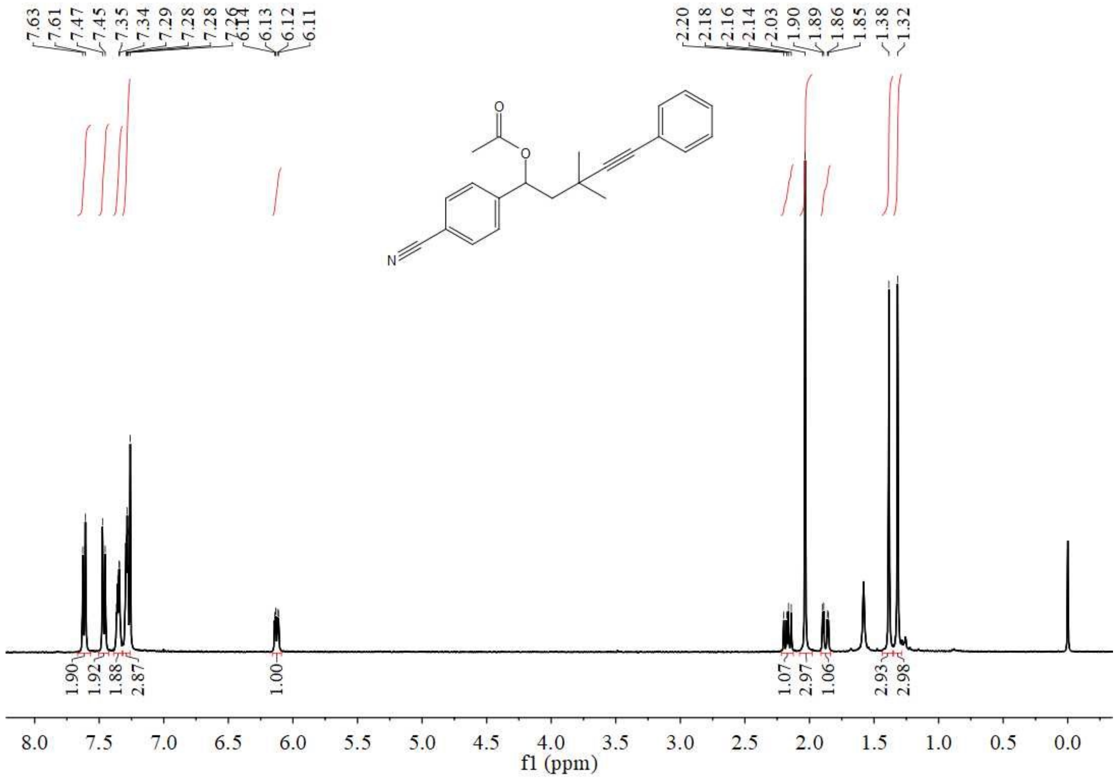

^{13}C NMR (101 MHz, $CDCl_3$) of **4o**

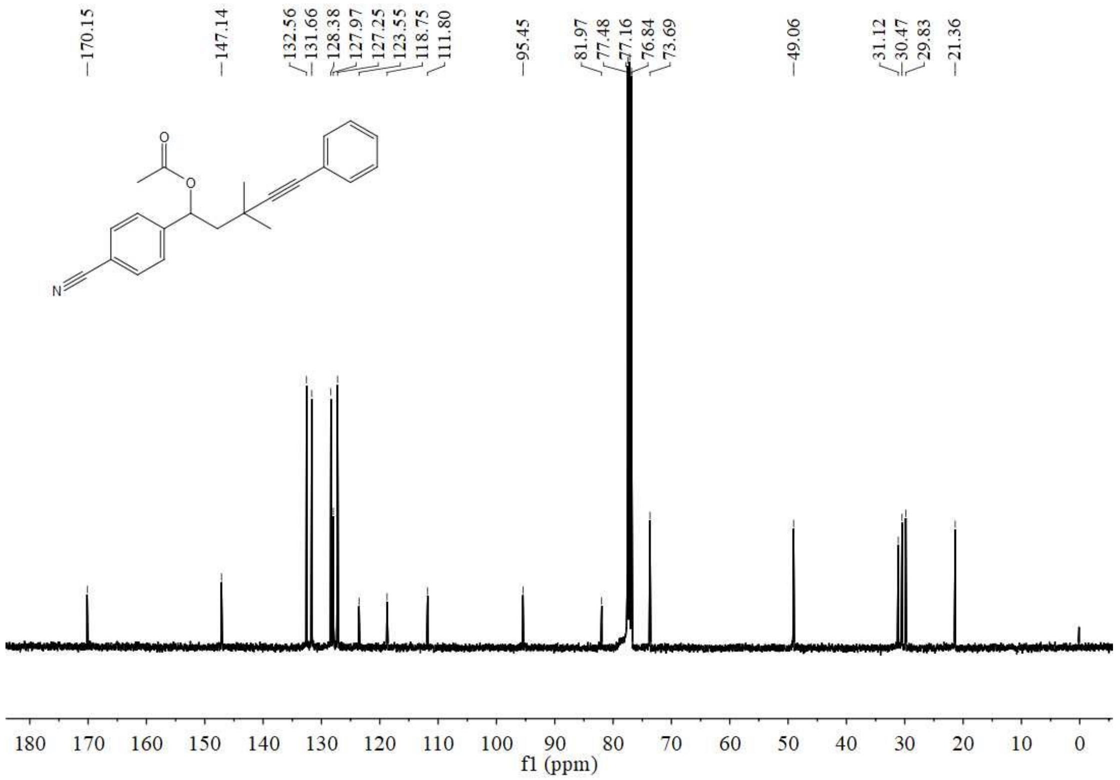

^{1}H NMR (400 MHz, $CDCl_3$) of **4p**

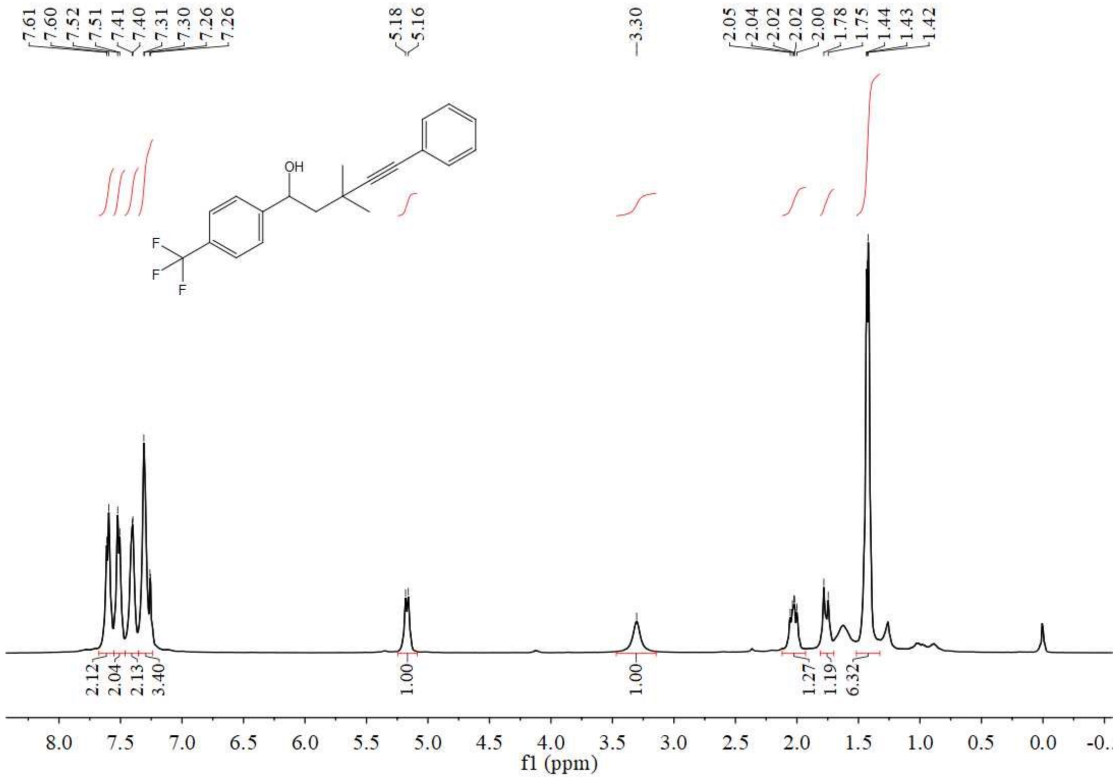

^{13}C NMR (101 MHz, $CDCl_3$) of **4p**

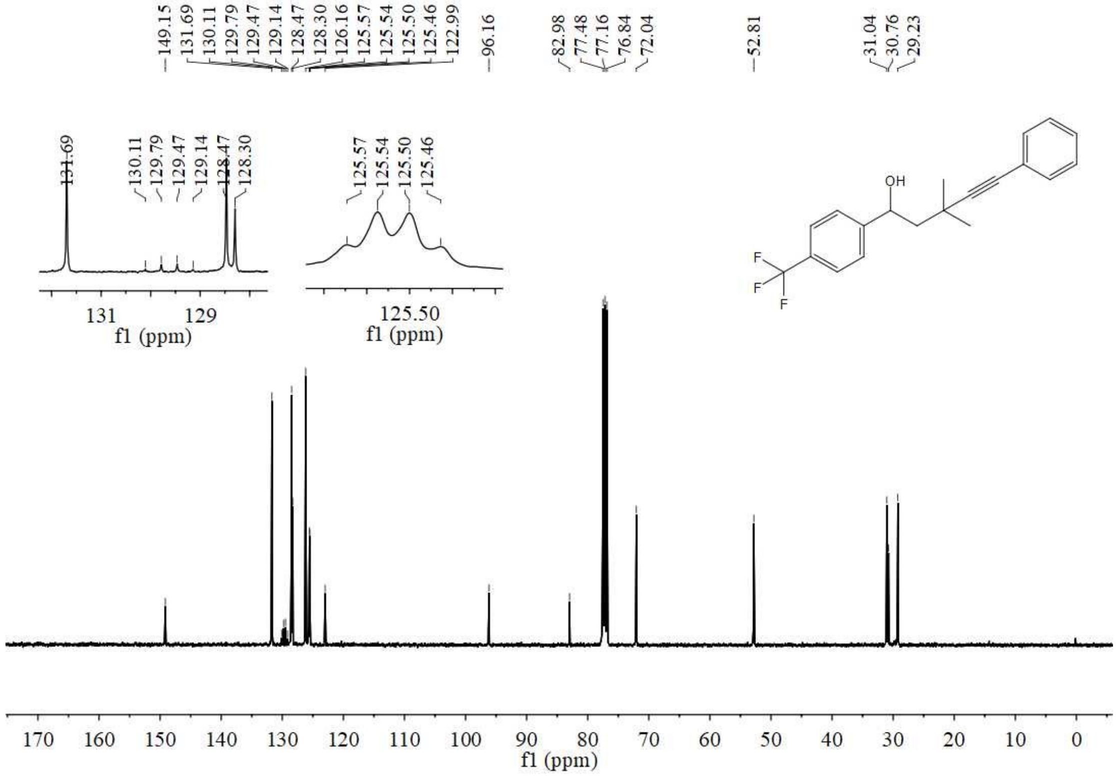

^{19}F NMR (376 MHz, $CDCl_3$) of **4p**

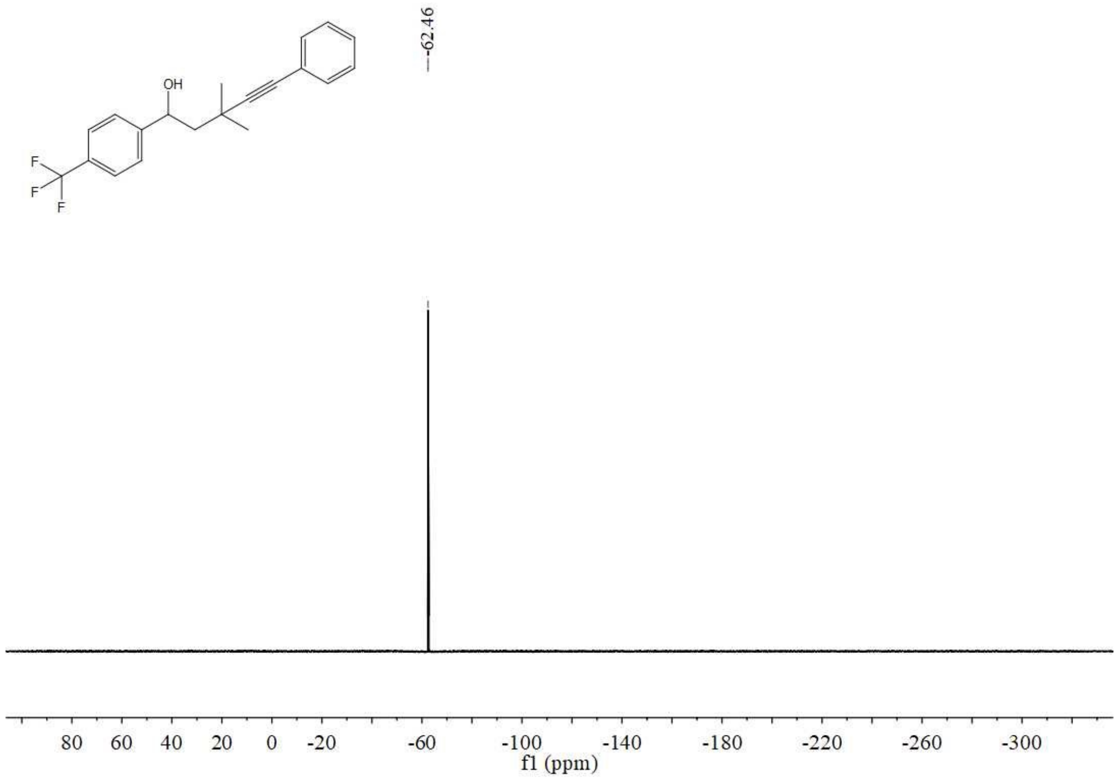

^{1}H NMR (400 MHz, $CDCl_3$) of **4q**

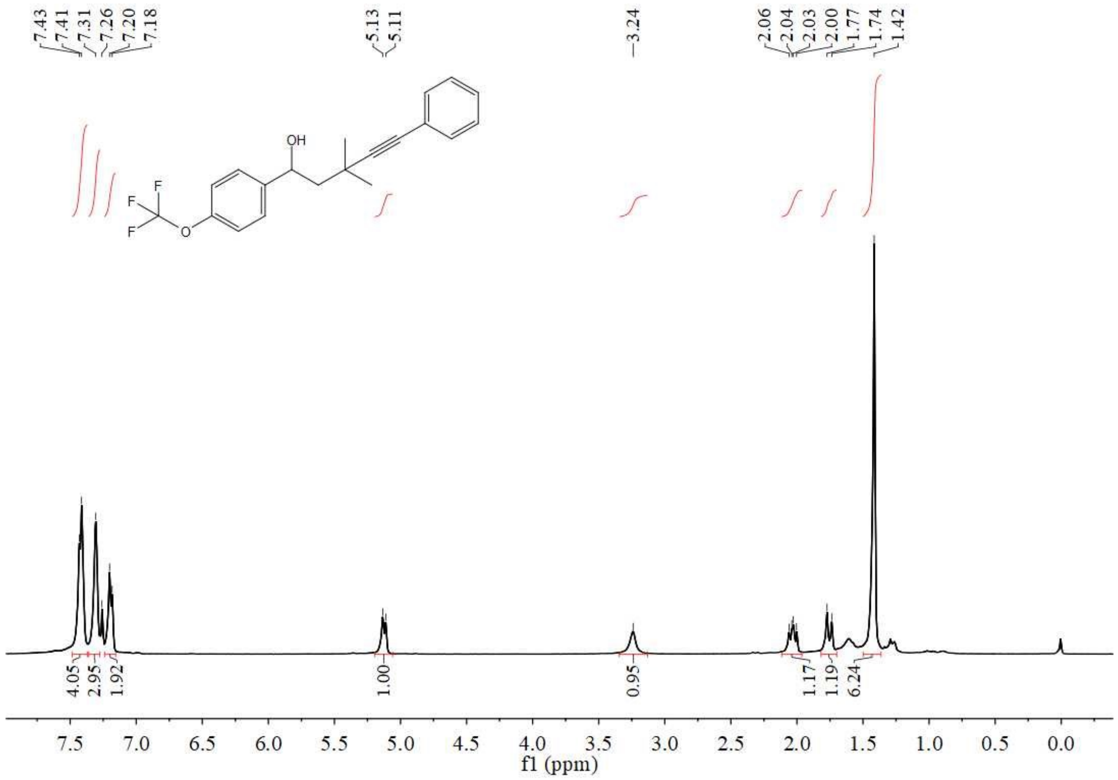

^{13}C NMR (101 MHz, CDCl$_3$) of **4q**

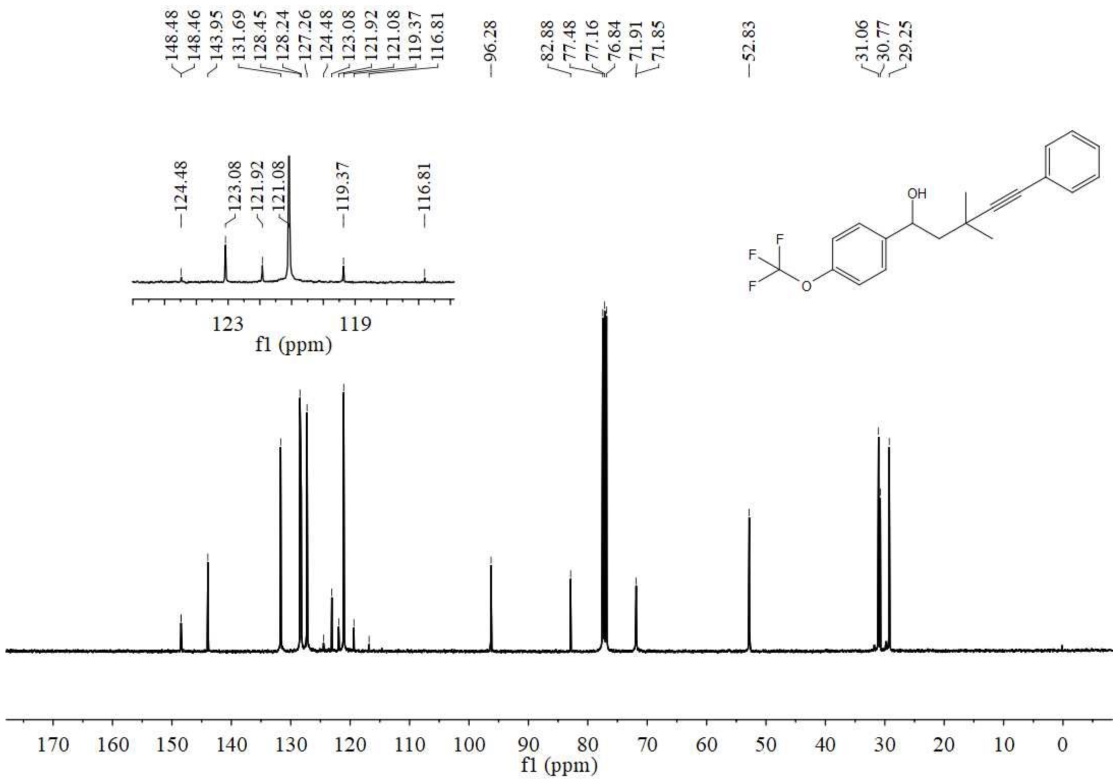

^{19}F NMR (376 MHz, CDCl$_3$) of **4q**

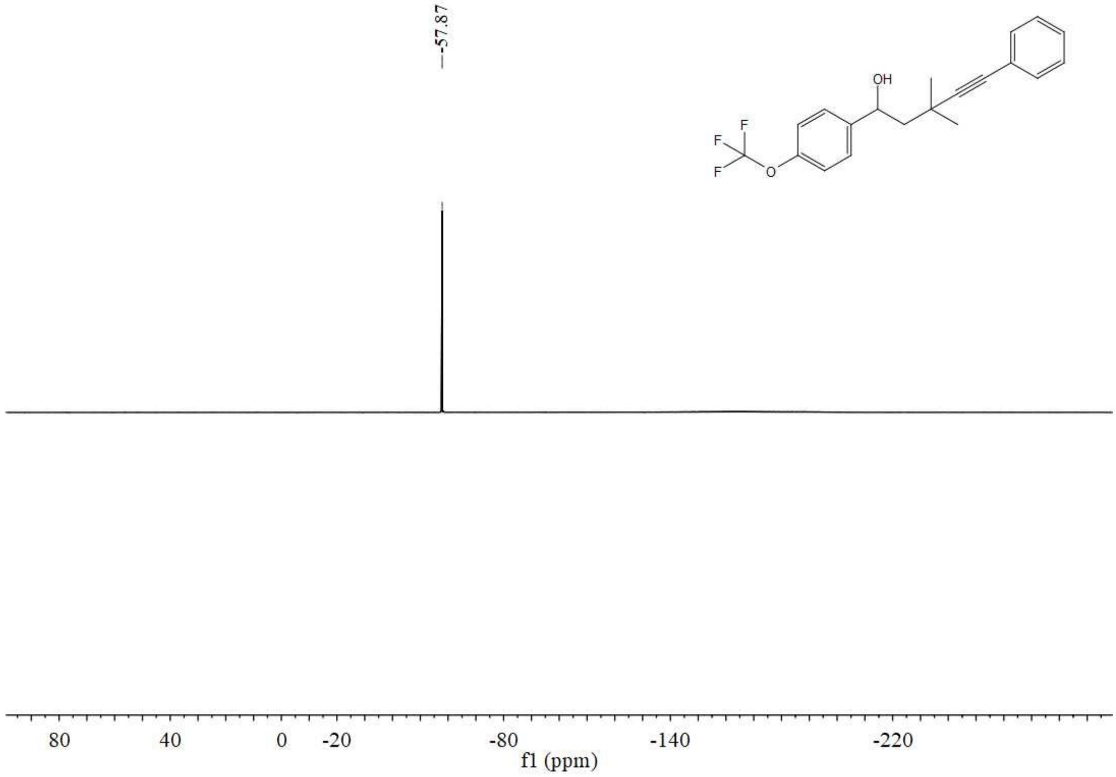

^{1}H NMR (400 MHz, $CDCl_3$) of **4r**

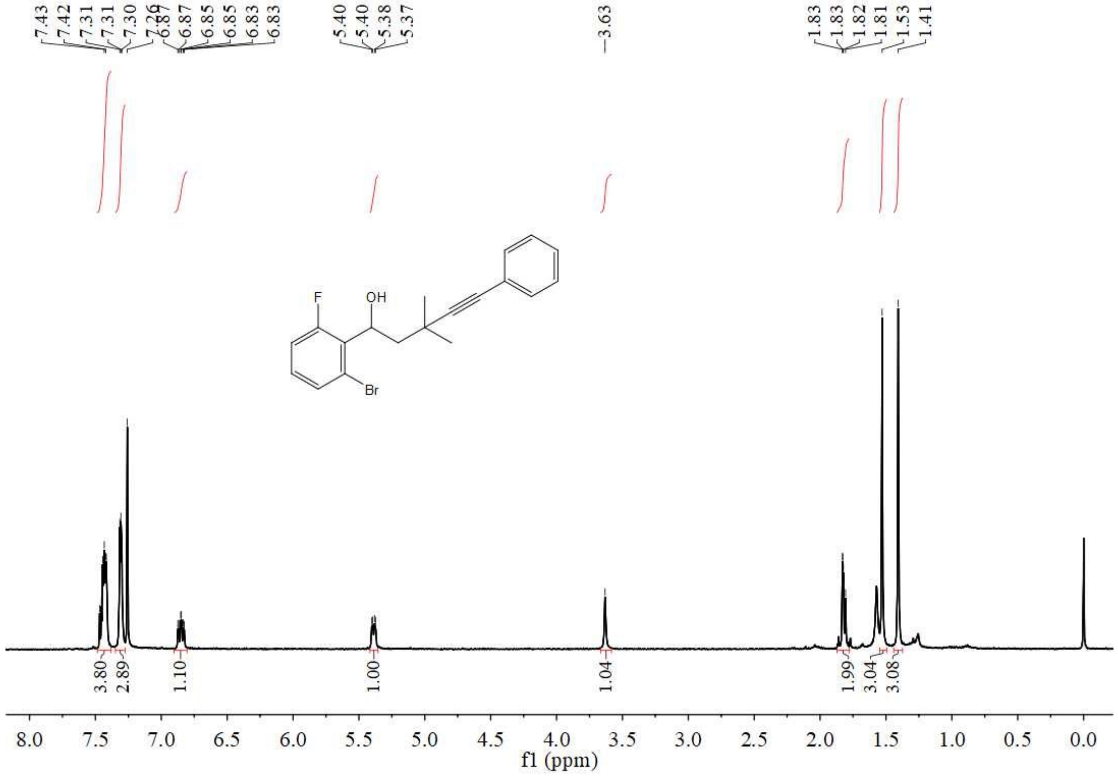

^{13}C NMR (101 MHz, $CDCl_3$) of **4r**

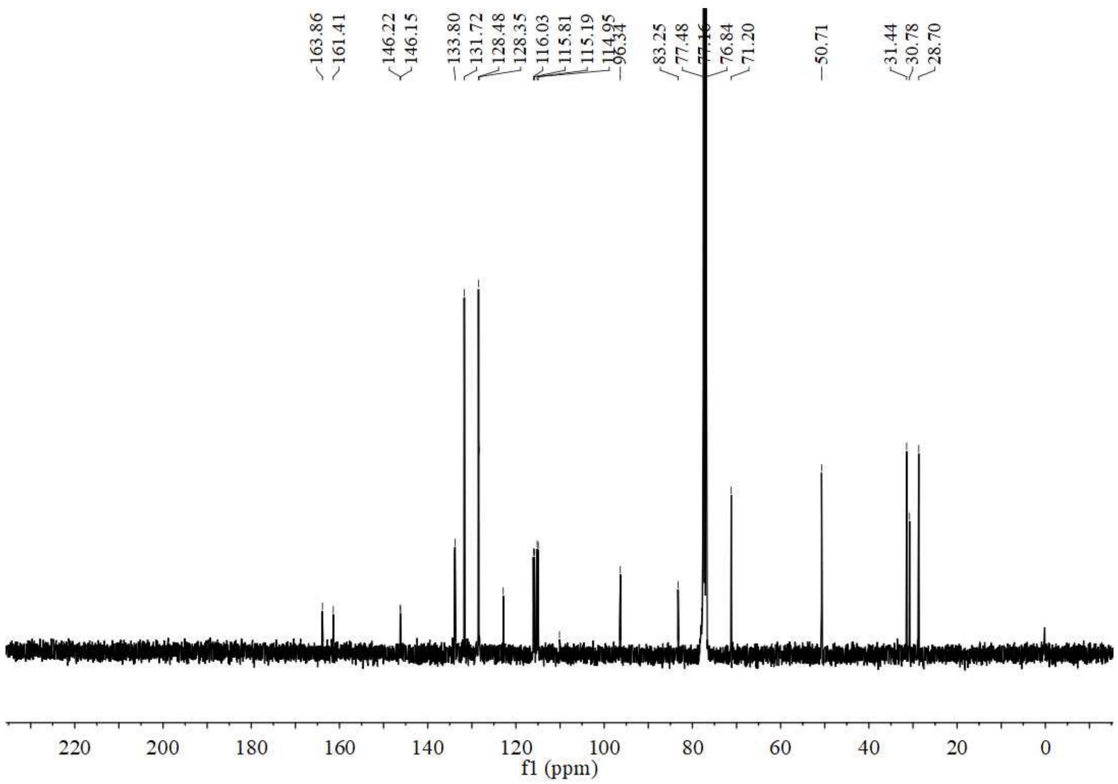

^{19}F NMR (376 MHz, $CDCl_3$) of **4r**

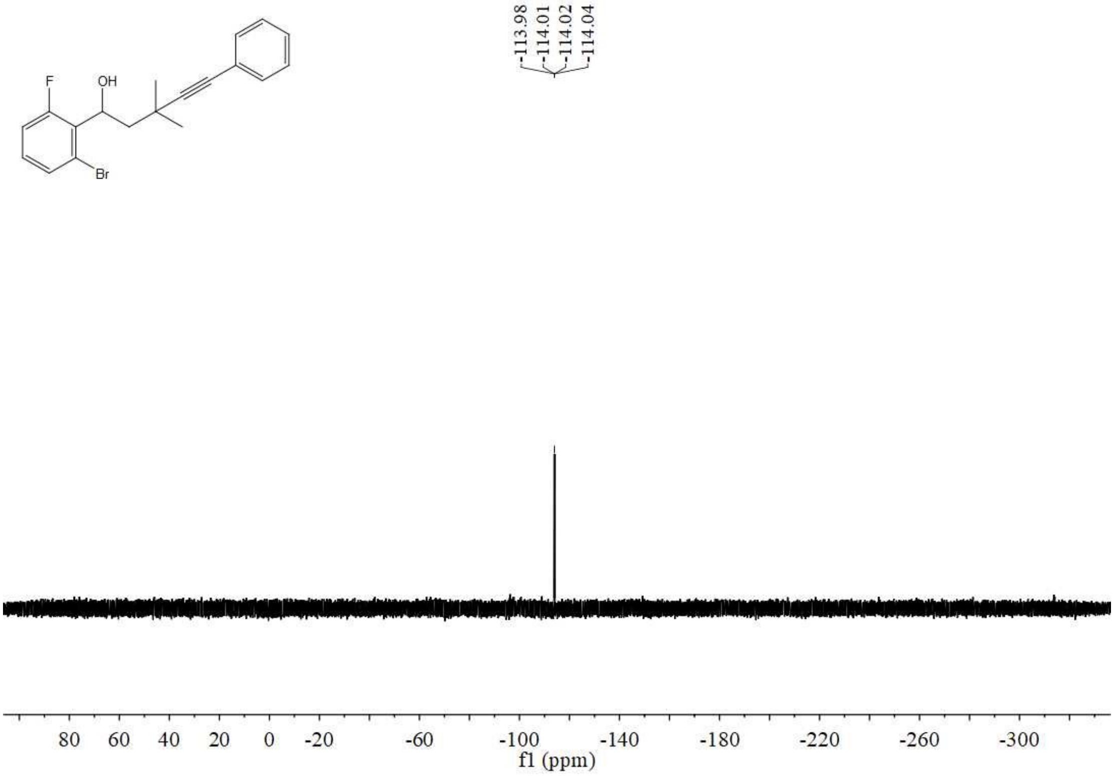

^{1}H NMR (400 MHz, $CDCl_3$) of **5a**

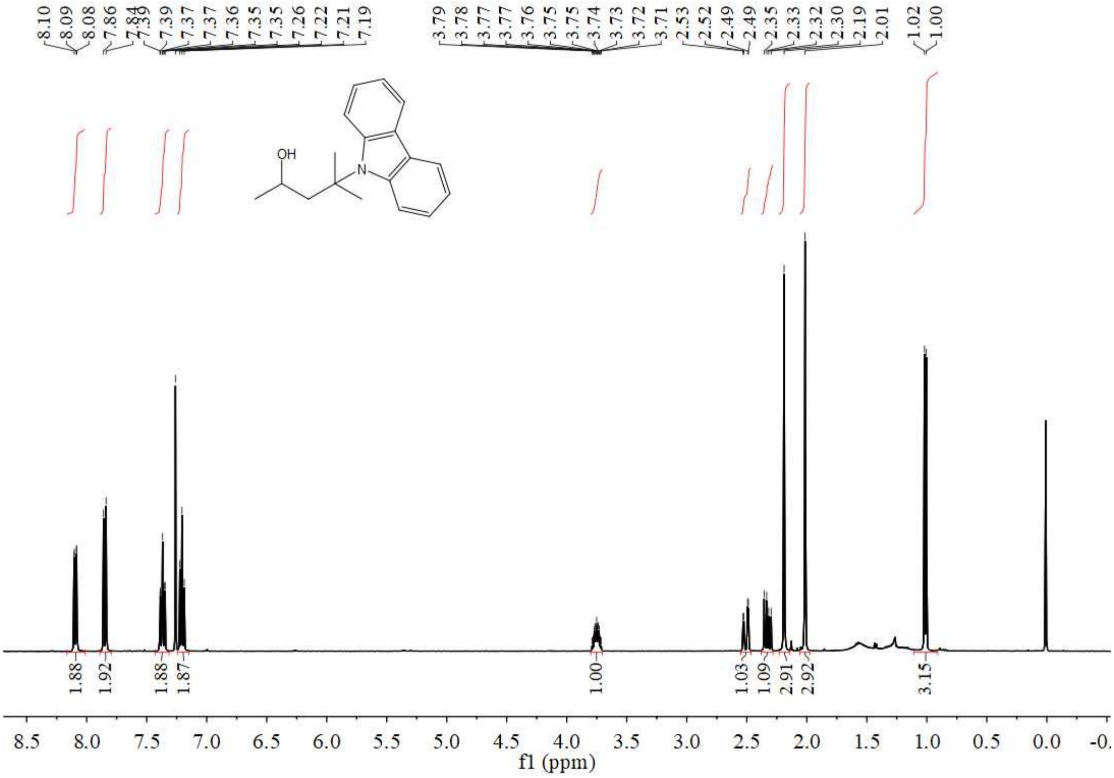

^{13}C NMR (101 MHz, CDCl3) of **5a**

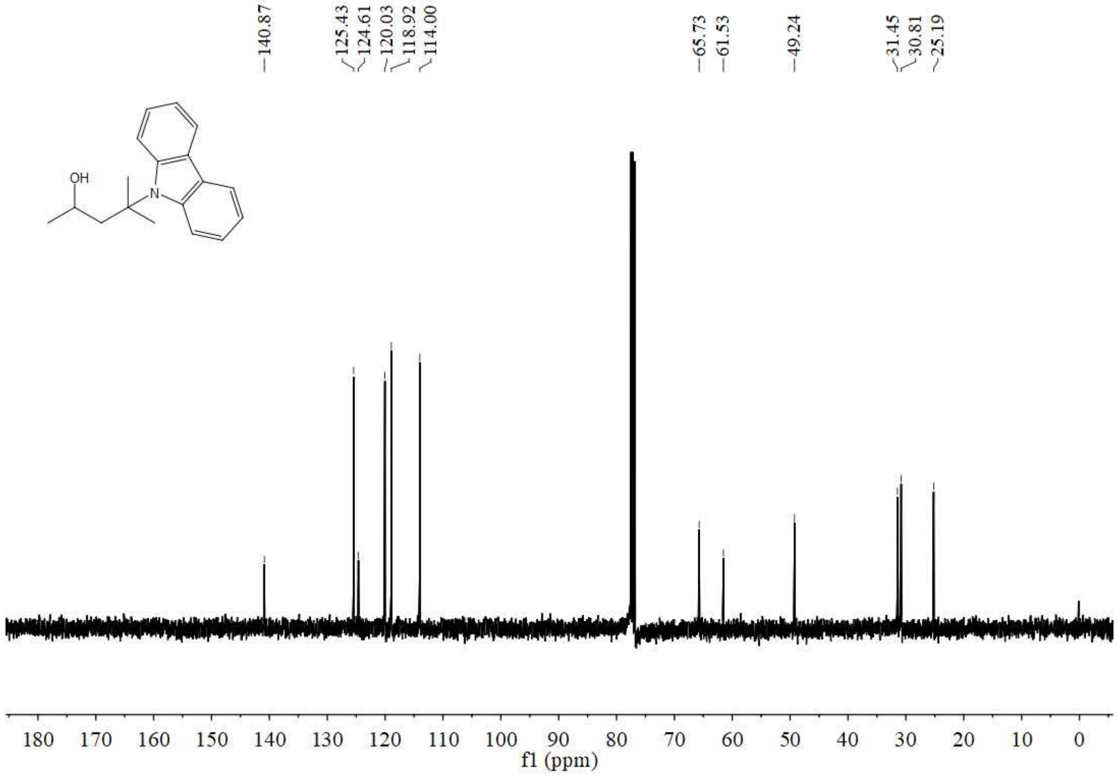

^{1}H NMR (400 MHz, CDCl3) of **5b**

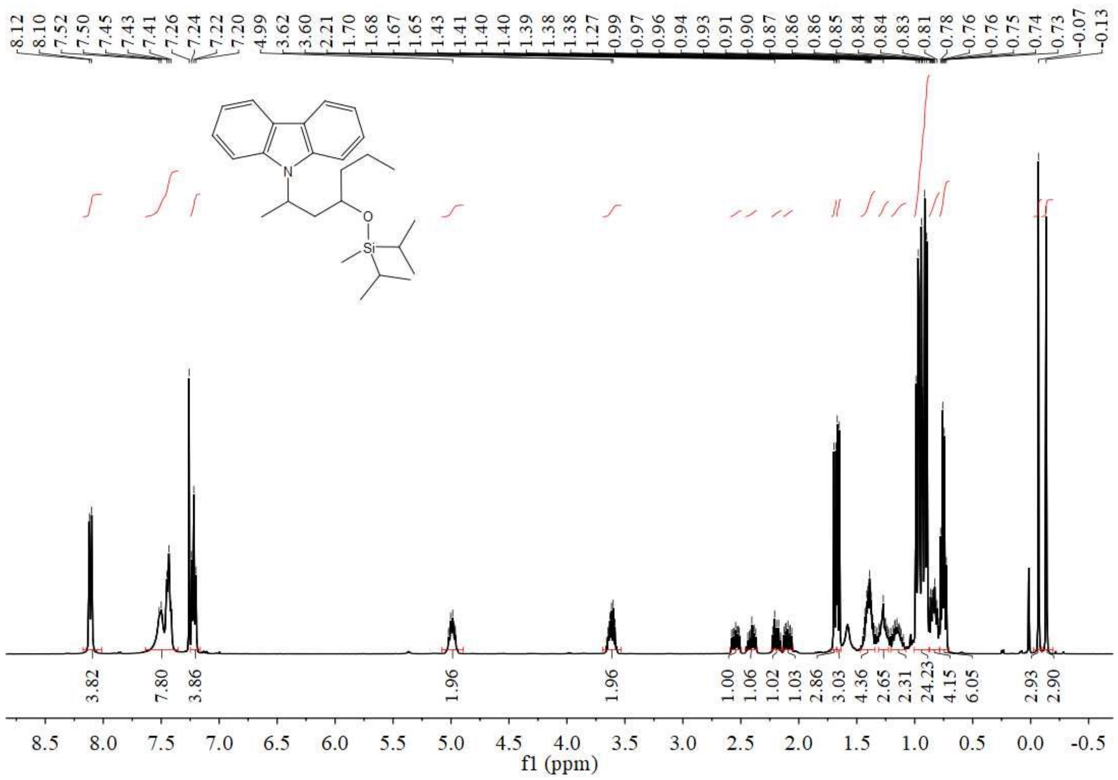

^{13}C NMR (101 MHz, $CDCl_3$) of **5b**

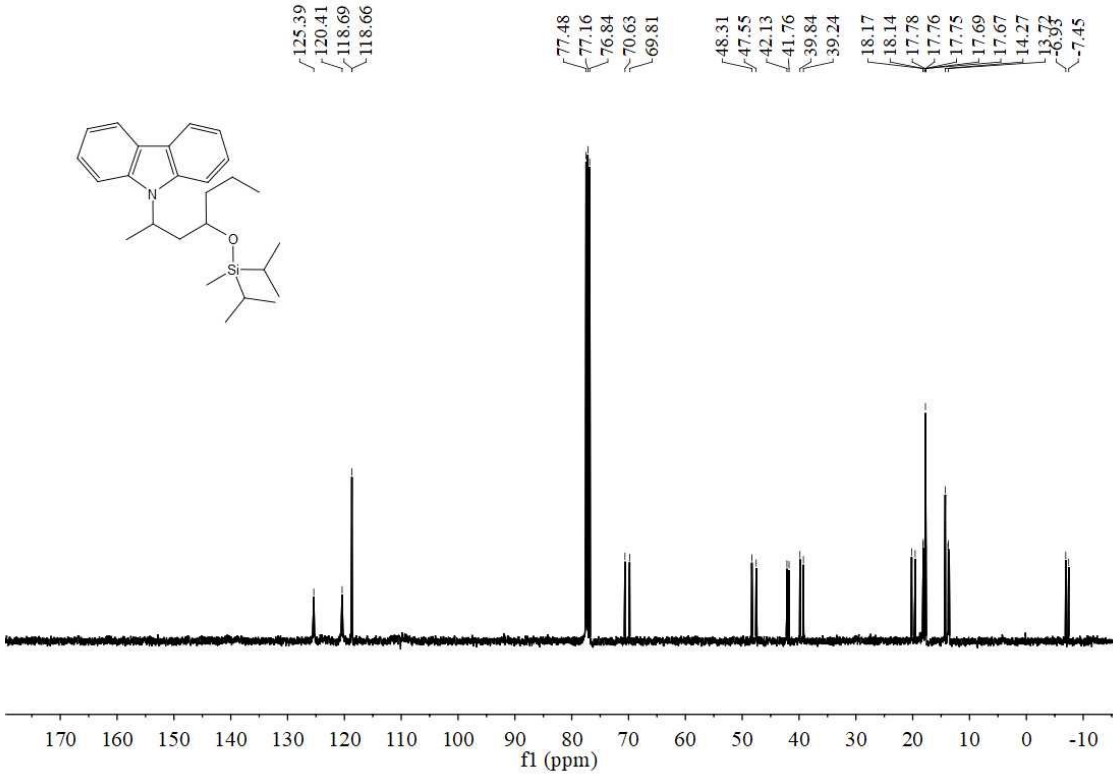

^{1}H NMR (400 MHz, $CDCl_3$) of **5c**

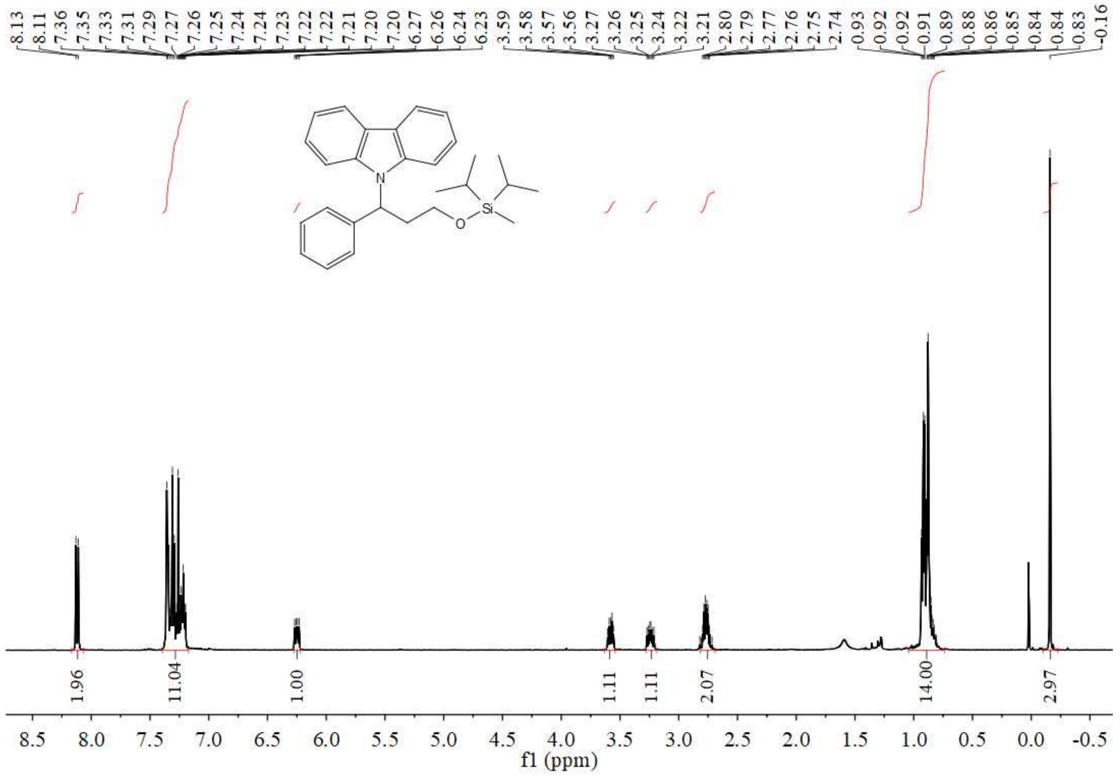

^{13}C NMR (101 MHz, $CDCl_3$) of **5c**

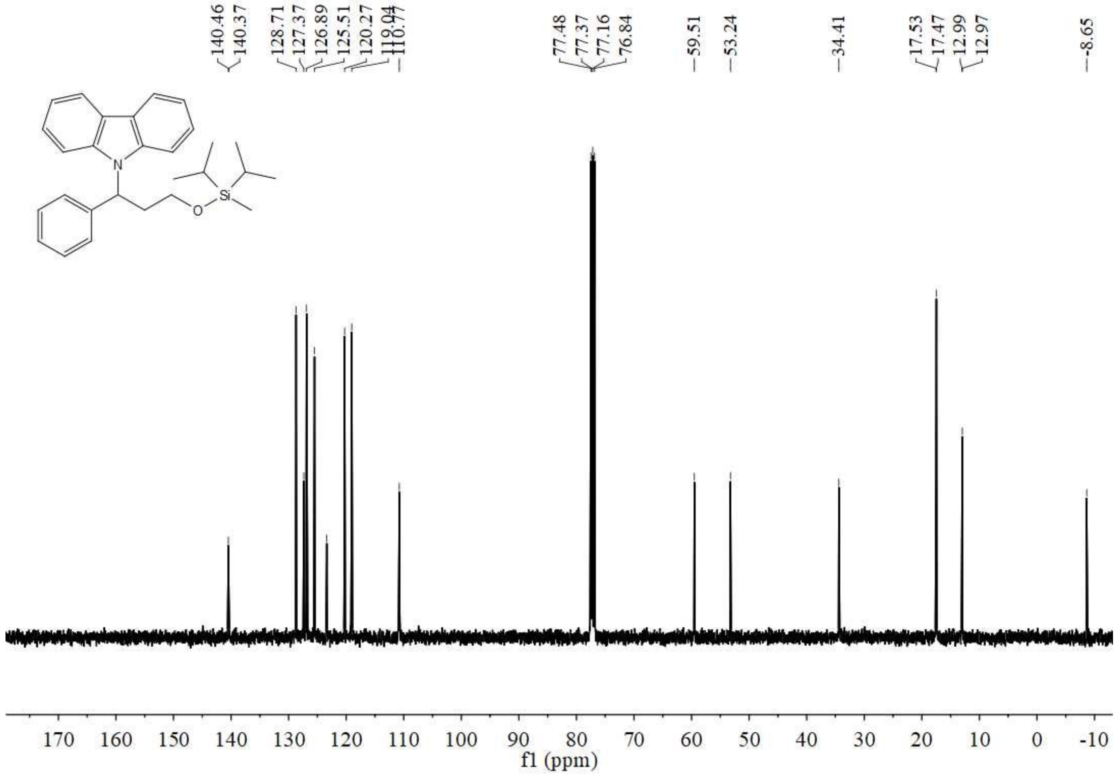

^{1}H NMR (400 MHz, $CDCl_3$) of **5d**

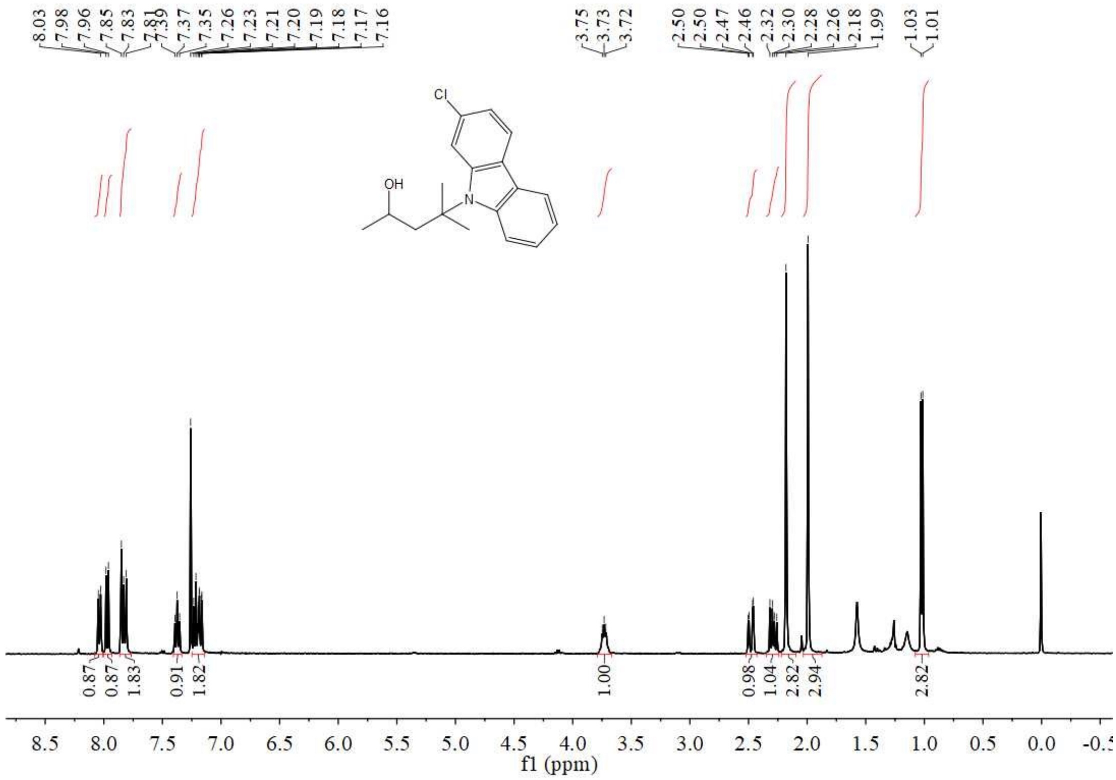

^{13}C NMR (101 MHz, $CDCl_3$) of **5d**

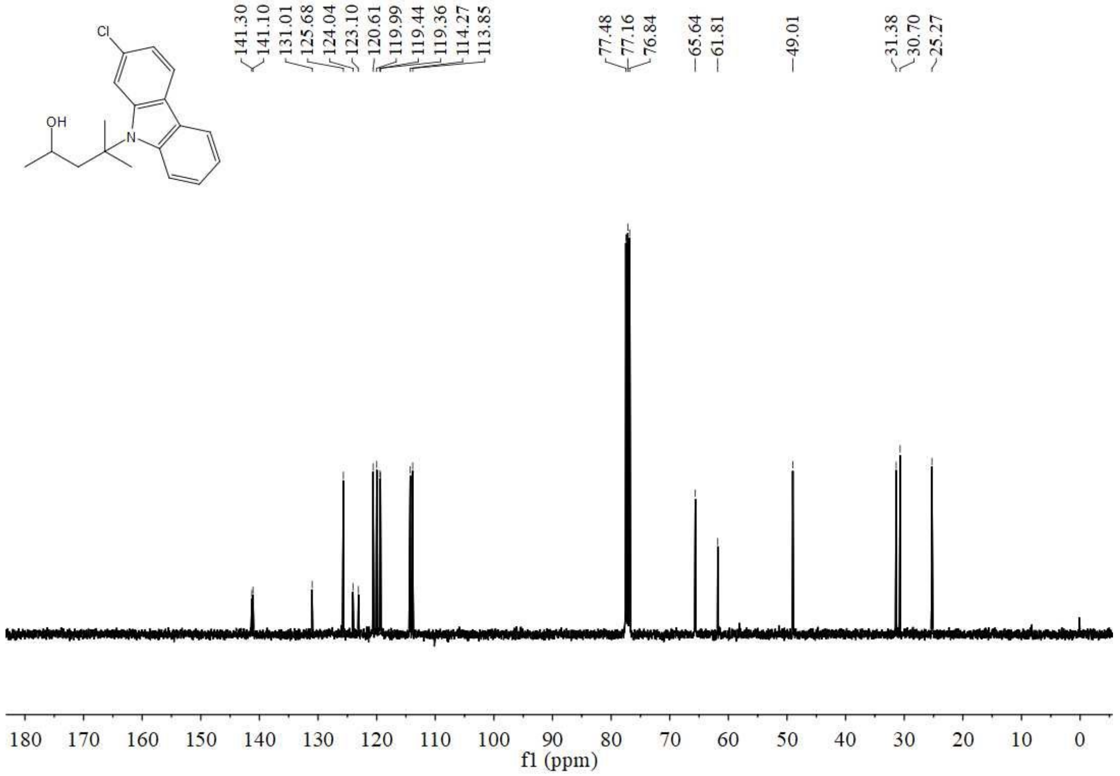

^{1}H NMR (400 MHz, $CDCl_3$) of **5e**

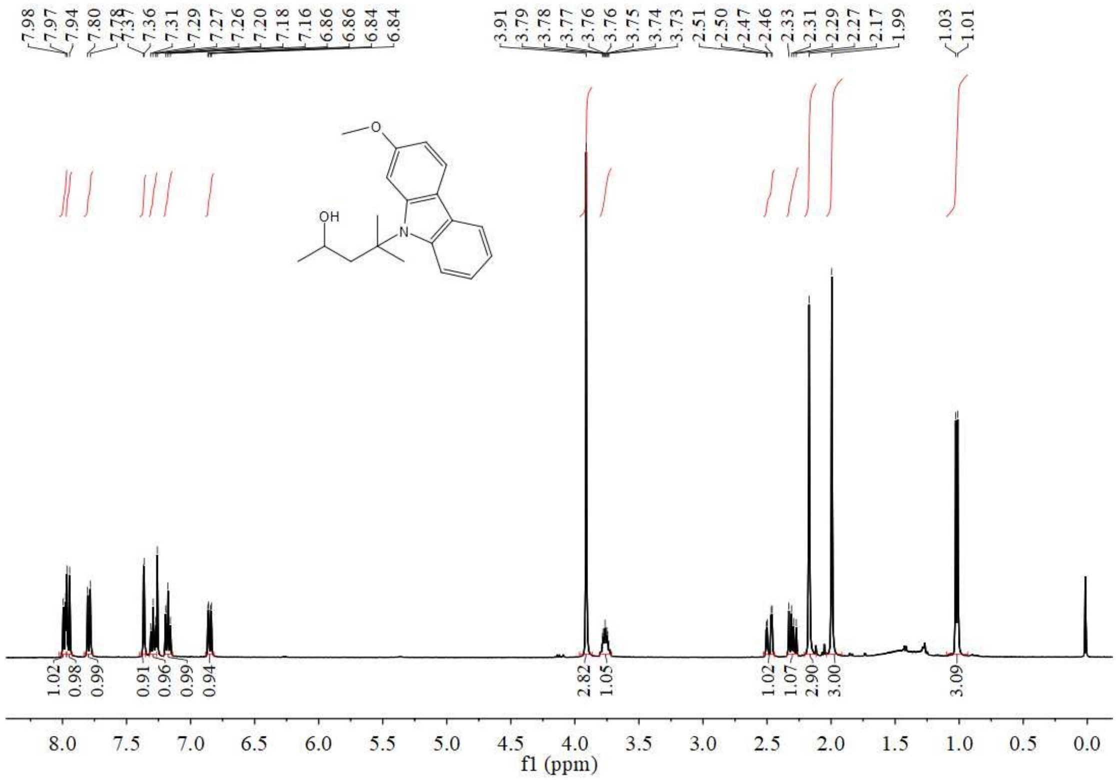

^{13}C NMR (101 MHz, $CDCl_3$) of **5e**

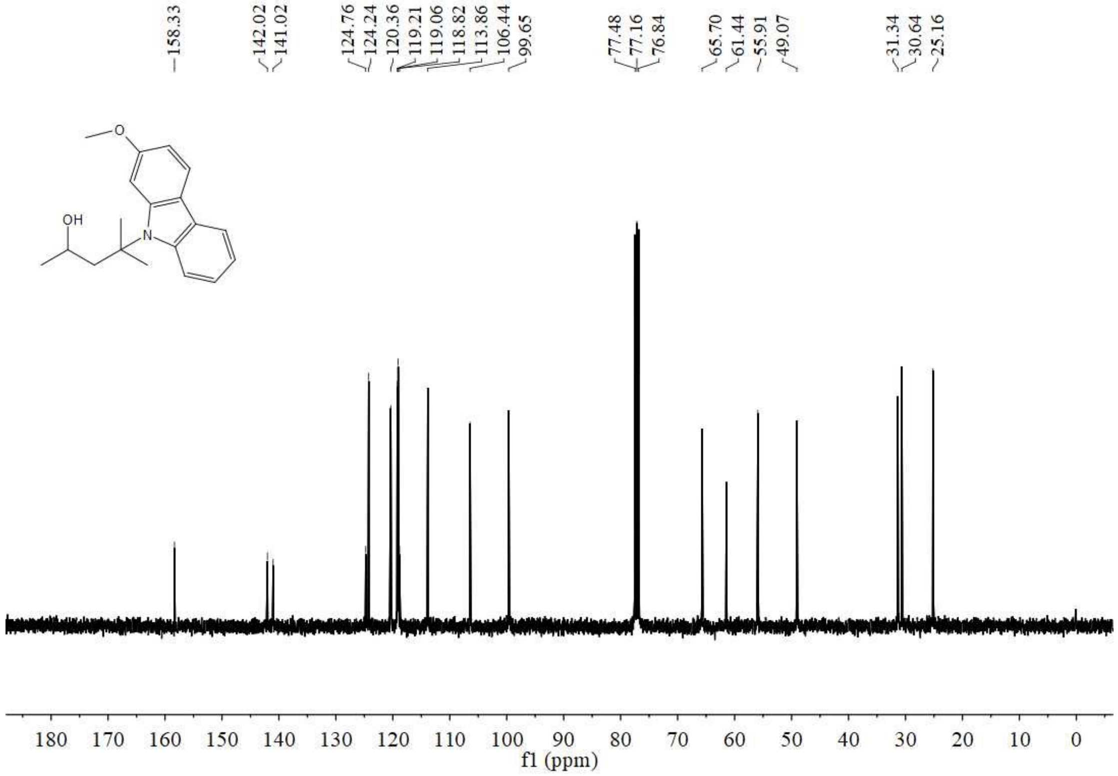

^1H NMR (400 MHz, $CDCl_3$) of **5f**

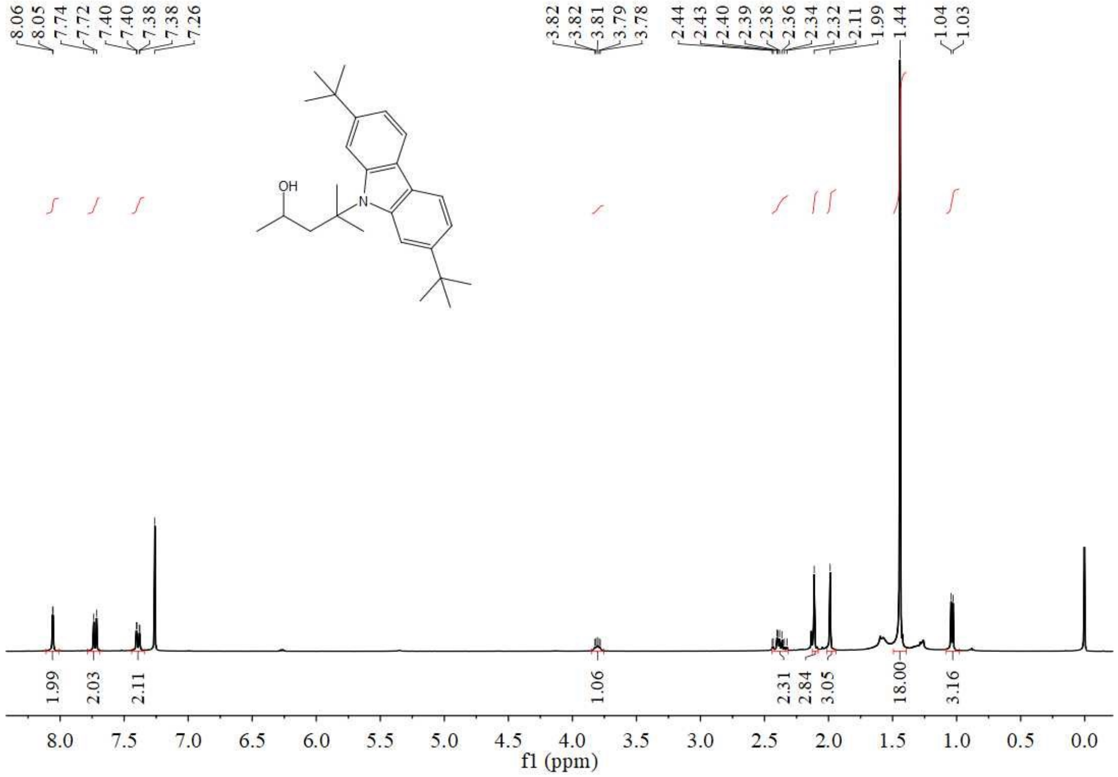

^{13}C NMR (101 MHz, $CDCl_3$) of **5f**

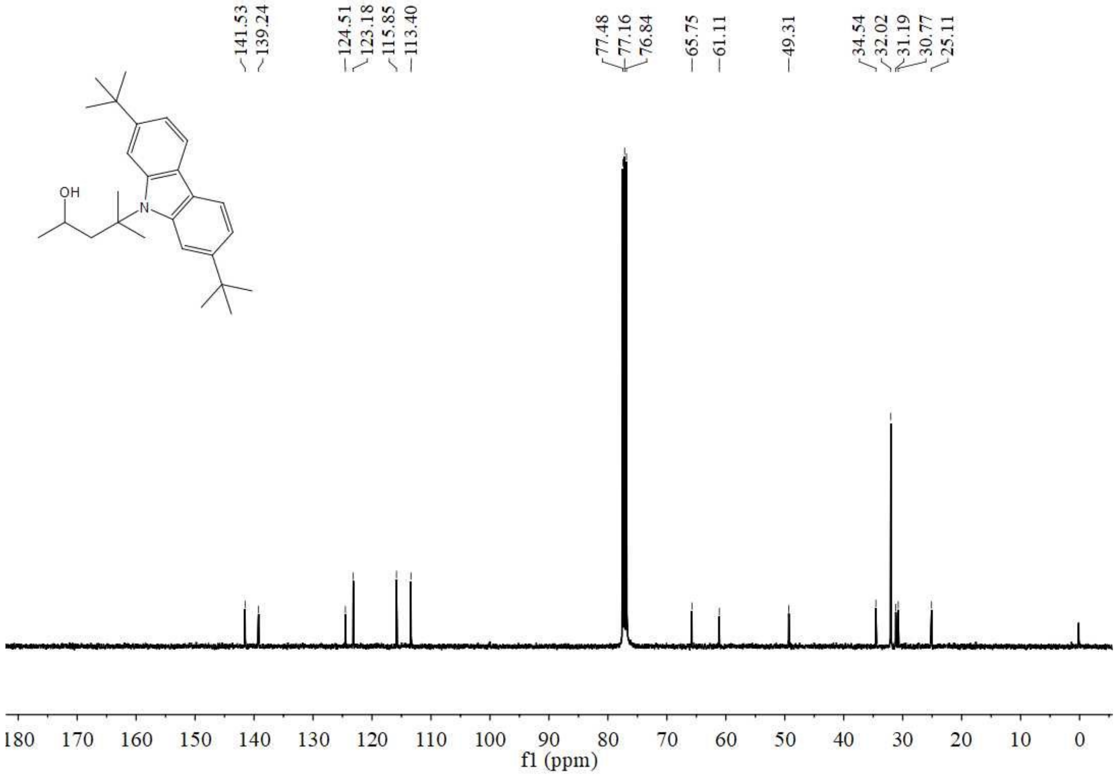

^{1}H NMR (400 MHz, $CDCl_3$) of **7**

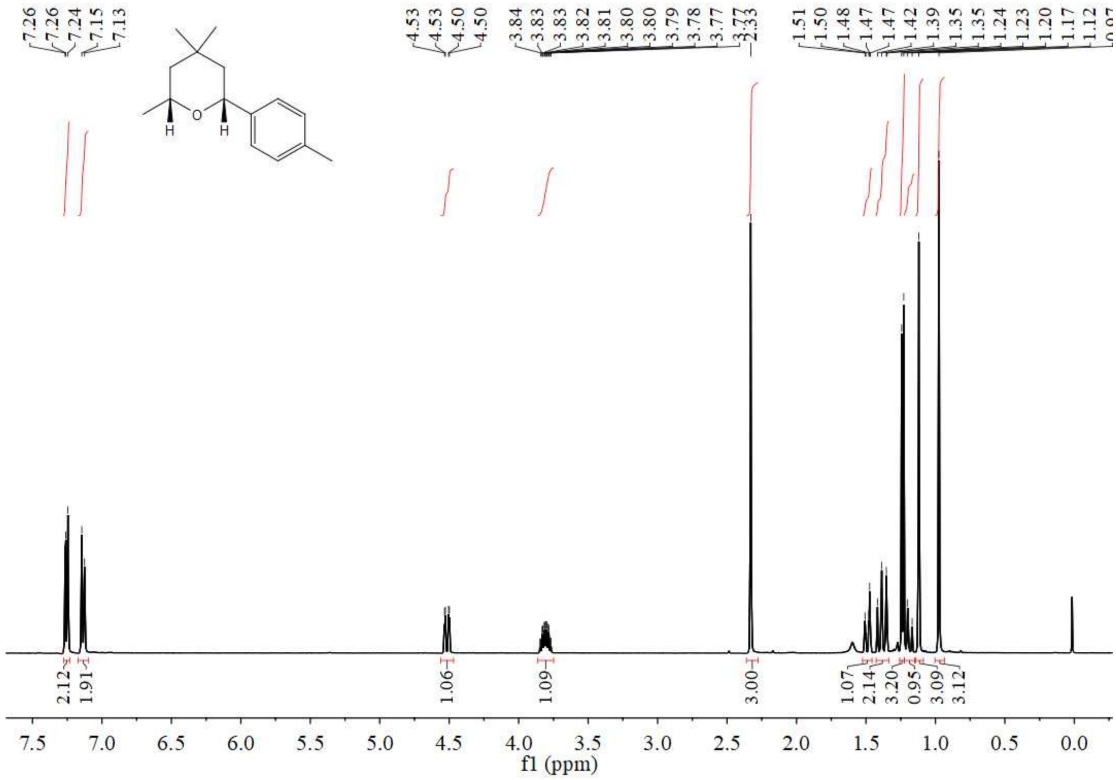

^{13}C NMR (101 MHz, $CDCl_3$) of **7**

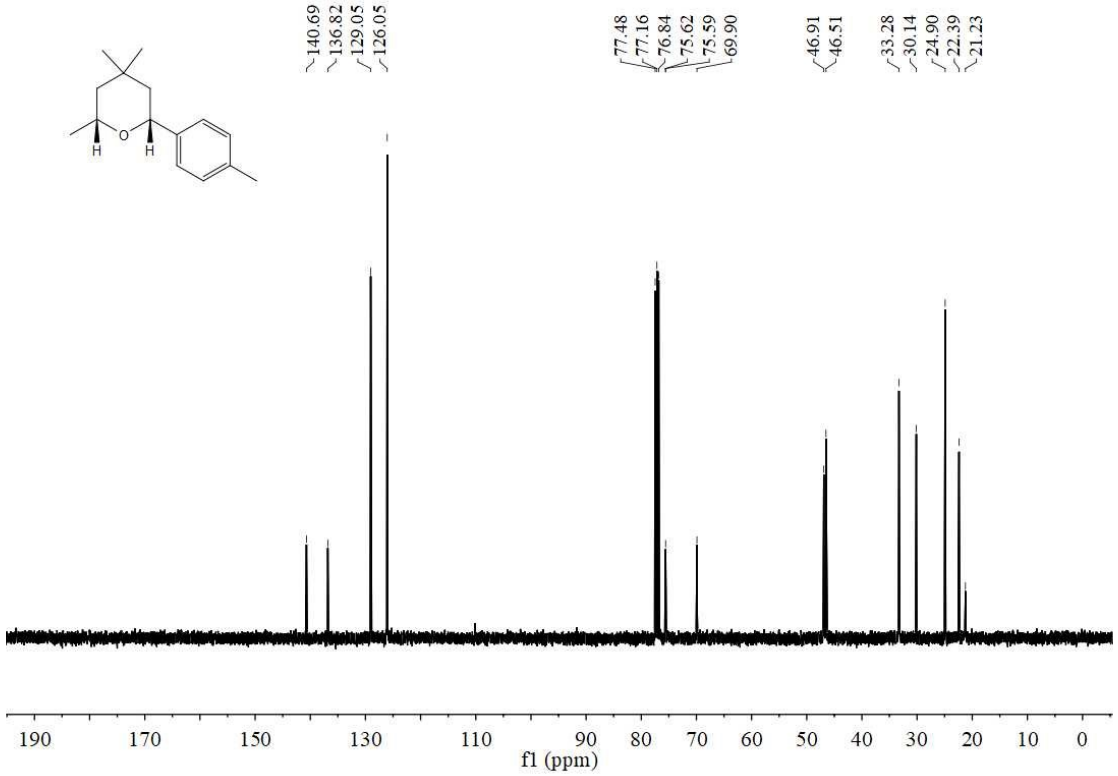

NOESY of **7**

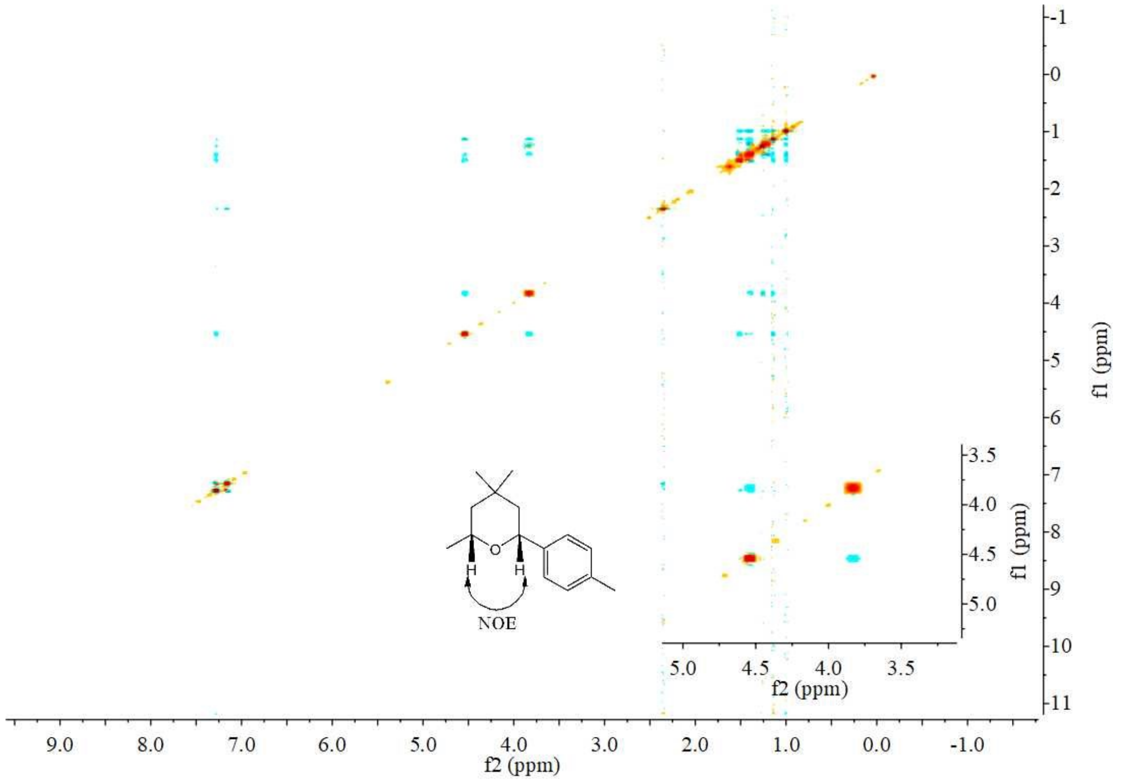

^{1}H NMR (400 MHz, $CDCl_3$) of **8**

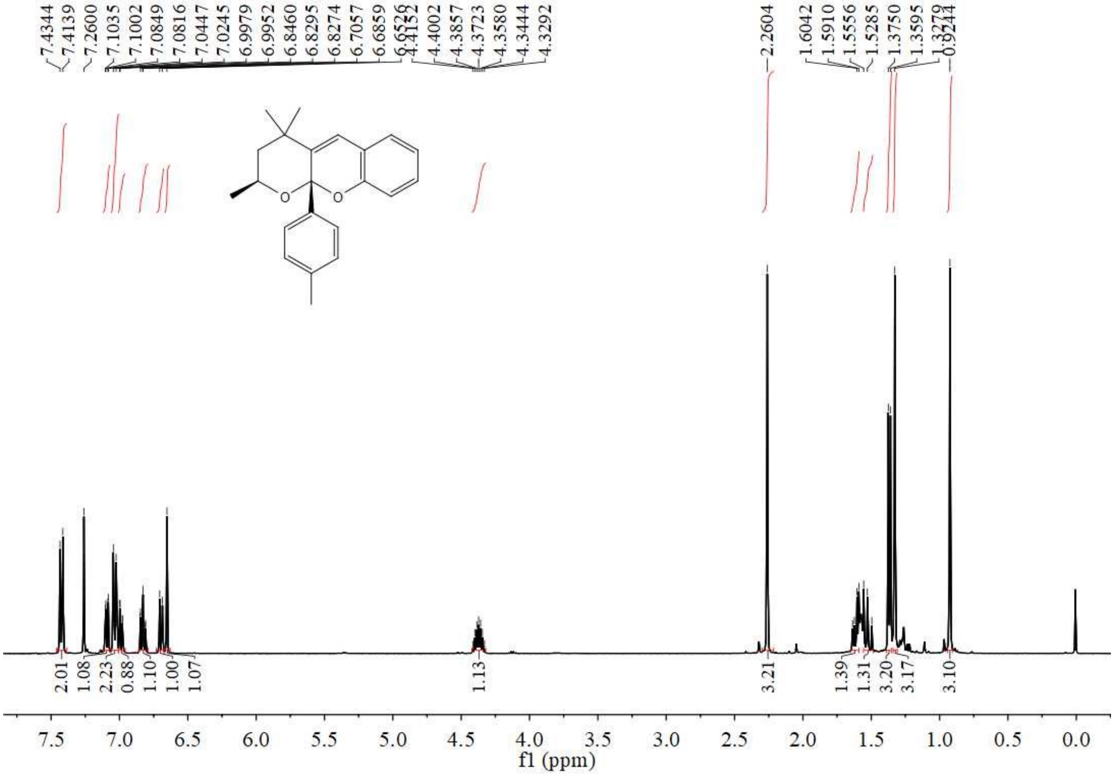

^{13}C NMR (101 MHz, $CDCl_3$) of **8**

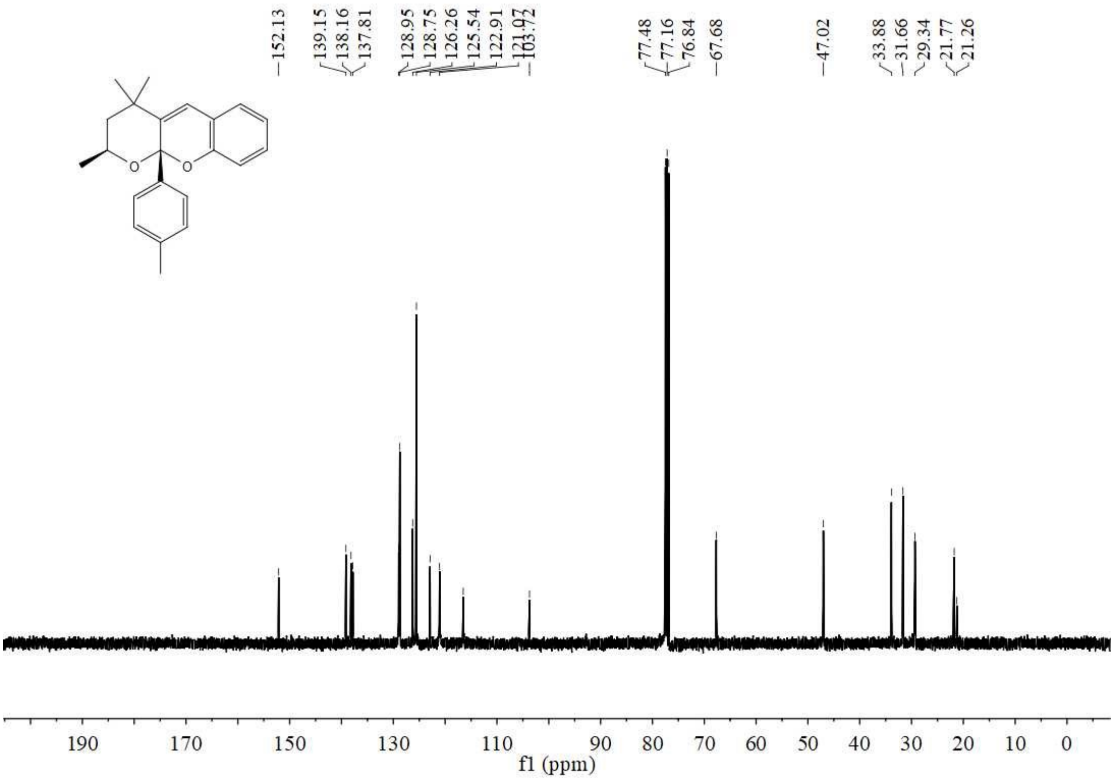

NOESY of **8**

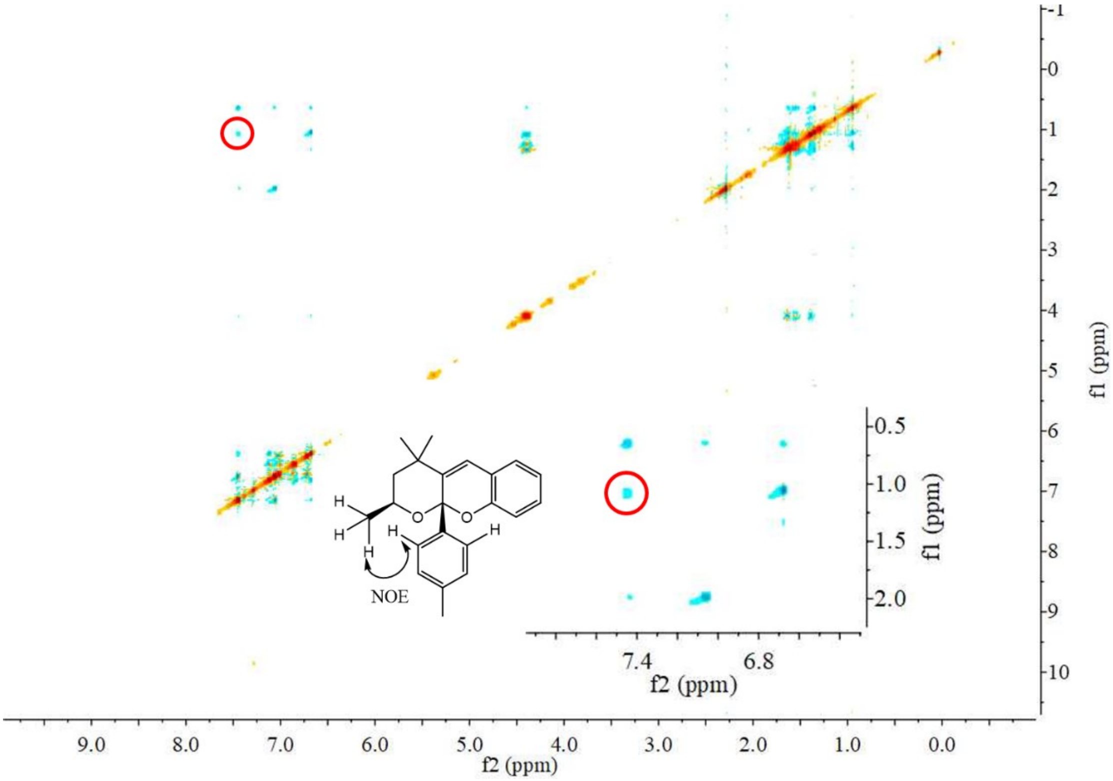

NOE
H
H
H
H
H
O
O
f1 (ppm)
f2 (ppm)
7.4
6.8
0.5
1.0
1.5
2.0
9.0
8.0
7.0
6.0
5.0
4.0
3.0
2.0
1.0
0.0
-1
0
1
2
3
4
5
6
7
8
9
10
